# Phylogenomic evidence supports past endosymbiosis, intracellular and horizontal gene transfer in *Cryptosporidium parvum*

**DOI:** 10.1186/gb-2004-5-11-r88

**Published:** 2004-10-19

**Authors:** Jinling Huang, Nandita Mullapudi, Cheryl A Lancto, Marla Scott, Mitchell S Abrahamsen, Jessica C Kissinger

**Affiliations:** 1 Center for Tropical and Emerging Global Diseases, University of Georgia, Athens, GA 30602, USA; 2 Department of Genetics, University of Georgia, Athens, GA 30602, USA; 3 Veterinary and Biomedical Sciences, University of Minnesota, St Paul, MN 55108, USA

## Abstract

**Background:**

The apicomplexan parasite *Cryptosporidium parvum *is an emerging pathogen capable of causing illness in humans and other animals and death in immunocompromised individuals. No effective treatment is available and the genome sequence has recently been completed. This parasite differs from other apicomplexans in its lack of a plastid organelle, the apicoplast. Gene transfer, either intracellular from an endosymbiont/donor organelle or horizontal from another organism, can provide evidence of a previous endosymbiotic relationship and/or alter the genetic repertoire of the host organism. Given the importance of gene transfers in eukaryotic evolution and the potential implications for chemotherapy, it is important to identify the complement of transferred genes in *Cryptosporidium*.

**Results:**

We have identified 31 genes of likely plastid/endosymbiont (n = 7) or prokaryotic (n = 24) origin using a phylogenomic approach. The findings support the hypothesis that *Cryptosporidium *evolved from a plastid-containing lineage and subsequently lost its apicoplast during evolution. Expression analyses of candidate genes of algal and eubacterial origin show that these genes are expressed and developmentally regulated during the life cycle of *C. parvum*.

**Conclusions:**

*Cryptosporidium *is the recipient of a large number of transferred genes, many of which are not shared by other apicomplexan parasites. Genes transferred from distant phylogenetic sources, such as eubacteria, may be potential targets for therapeutic drugs owing to their phylogenetic distance or the lack of homologs in the host. The successful integration and expression of the transferred genes in this genome has changed the genetic and metabolic repertoire of the parasite.

## Abstract 2

An analysis of *Cryptosporidium parvum* genes of likely endosymbiont or prokaryotic origin supports the hypothesis that *C. arvum* evolved from a plastid-containing lineage.

## Background

*Cryptosporidium *is a member of the Apicomplexa, a eukaryotic phylum that includes several important parasitic pathogens such as *Plasmodium*, *Toxoplasma*, *Eimeria *and *Theileria*. As an emerging pathogen in humans and other animals, *Cryptosporidium *often causes fever, diarrhea, anorexia and other complications. Although cryptosporidial infection is often self-limiting, it can be persistent and fatal for immunocompromised individuals. So far, no effective treatment is available [1]. Furthermore, because of its resistance to standard chlorine disinfection of water, *Cryptosporidium *continues to be a security concern as a potential water-borne bioterrorism agent [2].

*Cryptosporidium *is phylogenetically quite distant from the hemosporidian and coccidian apicomplexans [3] and, depending on the molecule and method used, is either basal to all Apicomplexa examined thus far, or is the sister group to the gregarines [4,5]. It is unusual in several respects, notably for the lack of the apicoplast organelle which is characteristic of all other apicomplexans that have been examined [6,7]. The apicoplast is a relict plastid hypothesized to have been acquired by an ancient secondary endosymbiosis of a pre-alveolate eukaryotic cell with an algal cell [8]. All that remains of the endosymbiont in Coccidia and Haemosporidia is a plastid organelle surrounded by four membranes [9]. The apicoplast retains its own genome, but this is much reduced (27-35 kilobases (kb)), and contains genes primarily involved in the replication of the plastid genome [10,11]. In apicomplexans that have a plastid, many of the original plastid genes appear to have been lost (for example, photosynthesis genes) and some genes have been transferred to the host nuclear genome; their proteins are reimported into the apicoplast where they function [12]. Plastids acquired by secondary endosymbiosis are scattered among eukaryotic lineages, including cryptomonads, haptophytes, alveolates, euglenids and chlorarachnions [13-17]. Among the alveolates, plastids are found in dinoflagellates and most examined apicomplexans but not in ciliates. Recent studies on the nuclear-encoded, plastid-targeted glyceraldehyde-3-phosphate dehydrogenase (GAPDH) gene suggest a common origin of the secondary plastids in apicomplexans, some dinoflagellates, heterokonts, haptophytes and cryptomonads [8,18]. If true, this would indicate that the lineage that gave rise to *Cryptosporidium *contained a plastid, even though many of its descendants (for example, the ciliates) appear to lack a plastid. Although indirect evidence has been noted for the past existence of an apicoplast in *C. parvum *[19,20], no rigorous phylogenomic survey for nuclear-encoded genes of plastid or algal origin has been reported.

Gene transfers, either intracellular (IGT) from an endosymbiont or organelle to the host nucleus or horizontal (HGT) between species, can dramatically alter the biochemical repertoire of host organisms and potentially create structural or functional novelties [21-23]. In parasites, genes transferred from prokaryotes or other sources are potential targets for chemotherapy due to their phylogenetic distance or lack of a homolog in the host [24,25]. The detection of transferred genes in *Cryptosporidium *is thus of evolutionary and practical importance.

In this study, we use a phylogenomic approach to mine the recently sequenced genome of *C. parvum *(IOWA isolate; 9.1 megabases (Mb)) [7] for evidence of the past existence of an endosymbiont or apicoplast organelle and of other independent HGTs into this genome. We have detected genes of cyanobacterial/algal origin and genes acquired from other prokaryotic lineages in *C. parvum*. The fate of several of these transferred genes in *C. parvum *is explored by expression analyses. The significance of our findings and their impact on the genetic makeup of the parasite are discussed.

## Results

### BLAST analyses

From BLAST analyses, the genome of *Cryptosporidium*, like that of *Plasmodium falciparum *[26], is more similar overall to those of the plants *Arabidopsis *and *Oryza *than to any other non-apicomplexan organism currently represented in GenBank. The program Glimmer predicted 5,519 protein-coding sequences in the *C. parvum *genome, 4,320 of which had similarity to other sequences deposited in the GenBank nonredundant protein database. A significant number of these sequences, 936 (E-value < 10^-3^) or 783 (E-value < 10^-7^), had their most significant, non-apicomplexan, similarity to a sequence isolated from plants, algae, eubacteria (including cyanobacteria) or archaea (Table 1). To evaluate these observations further, phylogenetic analyses were performed, when possible, for each predicted protein in the entire genome.

### Phylogenomic analyses

**Figure 1:**
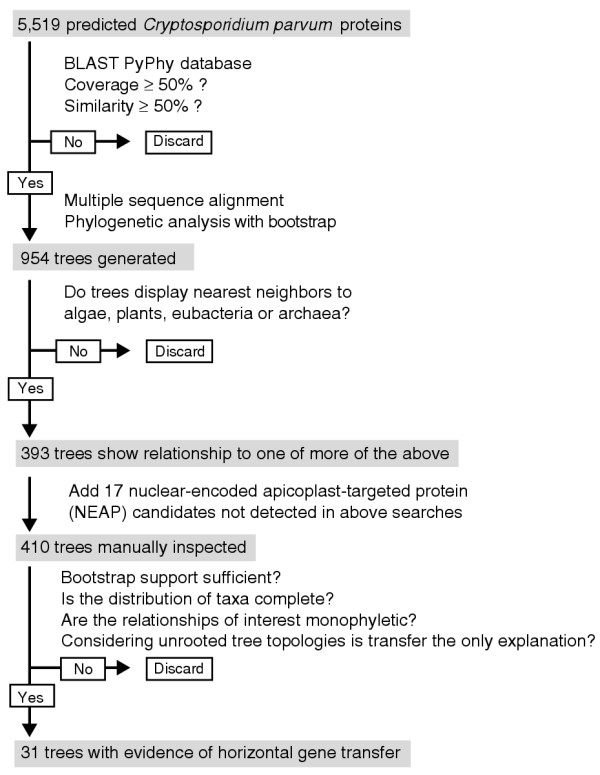
Phylogenomic analysis pipeline. The procedures used to analyze, assess and manipulate the protein-sequence data at each stage of the analysis are diagrammed.

The Glimmer-predicted protein-coding regions of the *C. parvum *genome (5,519 sequences) were used as input for phylogenetic analyses using the PyPhy program [27]. In this program, phylogenetic trees for each input sequence are analyzed to determine the taxonomic identity of the nearest neighbor relative to the input sequence at a variety of taxonomic levels, for example, genus, family, or phylum. Using stringent analysis criteria (see Materials and methods), 954 trees were constructed from the input set of 5,519 predicted protein sequences (Figure 1). Analysis of the nearest non-apicomplexan neighbor on the 954 trees revealed the following nearest neighbor relationships: eubacterial (115 trees), archaeal (30), green plant/algal (204), red algal (8), and glaucocystophyte (4); other alveolate (61) and other eukaryotes made up the remainder. As some input sequences may have more than one nearest neighbor of interest on a tree, a nonredundant total of 393 sequences were identified with nearest neighbors to the above lineages.

Searches of the *C. parvum *predicted gene set with the 551 *P. falciparum *predicted nuclear-encoded apicoplast-targeted proteins (NEAPs) yielded 40 significant hits (E-value < 10^-5^), 23 of which were also identified in the phylogenomic analyses. A combination of these two approaches identified 410 candidates requiring further detailed analyses. Of these candidates, the majority were eliminated after stringent criteria were applied because of ambiguous tree topologies, insufficient taxonomic sampling, lack of bootstrap support or the presence of clear vertical eukaryotic ancestry (see Materials and methods). Thirty-one genes survived the screen and were deemed to be either strong or likely candidates for gene transfer (Table 2).

**Figure 2:**
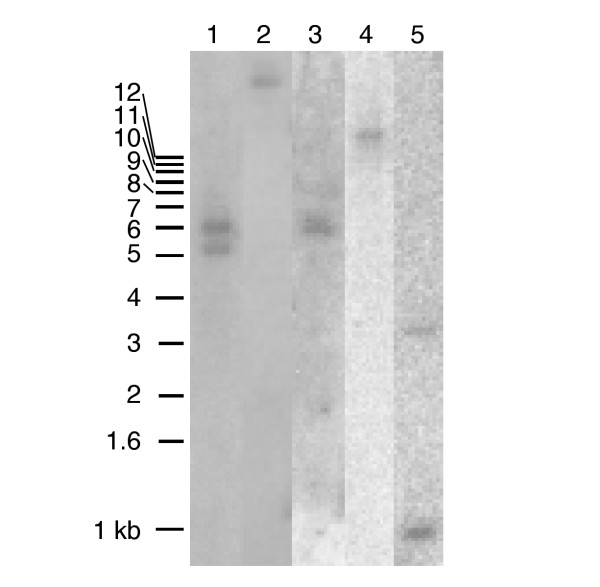
*Cryptosporidium parvum *genomic Southern blot. *C. parvum *genomic DNA, 5 μg per lane. Lanes were probed for the following genes: (1) aminopeptidase N; (2) glucose-6-phosphate isomerase; (3) leucine aminopeptidase; (4) pteridine transporter (BT-1); and (5) glutamine synthetase. Lanes (1-4) were restricted with *Bam*H1 and lane (5) with *Eco*R1. The ladder is shown in 1 kb increments. See Additional data file 1 for probes and methods.

Of the 31 recovered genes, several have been previously published or submitted to the GenBank [20], including those identified as having plant or eubacterial 'likeness' on the basis of similarity searches when the genome sequence was published [7]. The remaining sequences were further tested to rule out the possibility that they were artifacts (*C. parvum *oocysts are purified from cow feces which contain plant and bacterial matter). Two experiments were performed. In the first, nearly complete genomic sequences (generated in a different laboratory) from the closely related species *C. hominis *were screened using BLASTN for the existence of the predicted genes. Twenty out of 21 *C. parvum *sequences were identified in *C. hominis*. The remaining sequence was represented by two independently isolated expressed sequence tag (EST) sequences in the GenBank and CryptoDB databases (data not shown). In the second experiment, genomic Southern analyses of the IOWA isolate were carried out (Figure 2) for several of the genes of bacterial or plant origin. In each case, a band of the predicted size was identified (see Additional data file 1). The genes are not contaminants.

### Genes of cyanobacterial/algal origin

Extant *Cryptosporidium *species do not contain an apicoplast genome or any physical structure thought to represent an algal endosymbiont or the plastid organelle it contained [6,7]. The only possible remaining evidence of the past association of an endosymbiont or its cyanobacterially derived plastid organelle might be genes transferred from these genetic sources to the host genome prior to the physical loss of the endosymbiont or organelle itself. Several such genes were identified.

**Figure 3:**
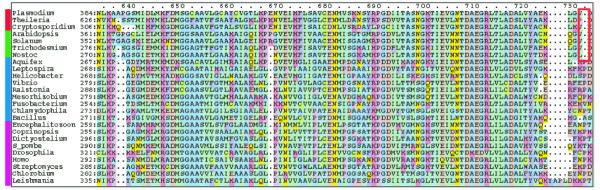
Region of leucine aminopeptidase multiple sequence alignment that illustrates several characters uniting apicomplexan sequences with plant and cyanobacterial sequences. The red box denotes an indel shared between apicomplexans, plants and cyanobacteria. The number preceeding each sequence is the position in the individual sequence at which this stretch of similarity begins. GenBank GI numbers for each sequence are as indicated in Additional data file 1. Colored boxes preceeding the alignment indicate the taxonomic group for the organisms named to the left. Red, apicomplexan; green, plant and cyanobacterial; blue, eubacterial; lavender, other protists and eukaryotes.

**Figure 4:**
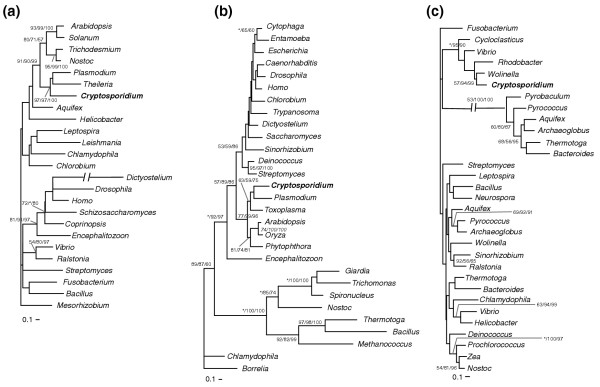
Phylogenetic analyses. **(a) **Leucine aminopeptidase; **(b) **glucose-6-phosphate isomerase; **(c) **tryptophan synthetase β subunit. Numbers above the branches (where space permits) show the puzzle frequency (with TREE-PUZZLE) and bootstrap support for both maximum parsimony and neighbor-joining analyses respectively. Asterisks indicate that support for this branch is below 50%. The scale is as indicated. GI accession numbers and alignments are provided in Additional data file 1.

A leucine aminopeptidase gene of cyanobacterial origin was found in the *C. parvum *nuclear genome. This gene is also present in the nuclear genome of other apicomplexan species (*Plasmodium*, *Toxoplasma *and *Eimeria*), as confirmed by similarity searches against ApiDB (see Materials and methods). In *P. falciparum*, leucine aminopeptidase is a predicted NEAP and possesses an amino-terminal extension with a putative transit peptide. Consistent with the lack of an apicoplast, this gene in *Cryptosporidium *contains no evidence of a signal peptide and the amino-terminal extension is reduced. Similarity searches of the GenBank nonredundant protein database revealed top hits to *Plasmodium*, followed by *Arabidopsis thaliana*, and several cyanobacteria including *Prochlorococcus*, *Nostoc *and *Trichodesmium*, and plant chloroplast precursors in *Lycopersicon esculentum *and *Solanum tuberosum *(data not shown). A multiple sequence alignment of the predicted protein sequences of leucine aminopeptidase reveals overall similarity and a shared indel among apicomplexan, plant and cyanobacterial sequences (Figure 3). Phylogenetic analyses strongly support a monophyletic grouping of *C. parvum *and other apicomplexan leucine aminopeptidase proteins with cyanobacteria and plant chloroplast precursors (Figure 4a). So far, this gene has not been detected in ciliates.

Another *C. parvum *nuclear-encoded gene of putative cyanobacterial origin is a protein of unknown function belonging to the biopterine transporter family (BT-1) (Table 2). Similarity searches with this protein revealed significant hits to other apicomplexans (for example, *P. falciparum*, *Theileria annulata*, *T. gondii*), plants (*Arabidopsis*, *Oryza*), cyanobacteria (*Trichodesmium*, *Nostoc *and *Synechocystis*), a ciliate (*Tetrahymena*) and the kinetoplastids (*Leishmania *and *Trypanosoma*). *Arabidopsis thaliana *apparently contains at least two copies of this gene; the protein of one (accession number NP_565734) is predicted by ChloroP [28] to be chloroplast-targeted, suggestive of its plastid derivation. The taxonomic distribution and sequence similarity of this protein with cyanobacterial and chloroplast homologs are also indicative of its affinity to plastids.

Only one gene of algal nuclear origin, glucose-6-phosphate isomerase (G6PI), was identified by the screen described here. Several other algal-like genes are probable, but their support was weaker (Table 2). A 'plant-like' G6PI has been described in other apicomplexan species (*P. falciparum*, *T. gondii *[29]) and a 'cyanobacterial-like' G6PI has been described in the diplomonads *Giardia intestinalis *and *Spironucleus *and the parabasalid *Trichomonas vaginalis *[30]. Figure 4b illustrates these observations nicely. At the base of the tree, the eukaryotic organisms *Giardia*, *Spironucleus *and *Trichomonas *group with the cyanobacterium *Nostoc*, as previously published. In the midsection of the tree, the G6PI of apicomplexans and ciliates forms a well-supported monophyletic group with the plants and the heterokont *Phytophthora*. The multiple protein sequence alignment of G6PI identifies several conserved positions shared exclusively by apicomplexans, *Tetrahymena*, plants and *Phytophthora*. This gene does not contain a signal or transit peptide and is not predicted to be targeted to the apicoplast in *P. falciparum*. The remainder of the tree shows a weakly supported branch including eubacteria, fungi and several eukaryotes. The eukaryotes are interrupted by the inclusion of G6PI from the eubacterial organisms *Escherichia coli *and *Cytophaga*. This relationship of *E. coli *G6PI and eukaryotic G6PI has been observed before and may represent yet another gene transfer [31].

### Genes of eubacterial (non-cyanobacterial) origin

Our study identified HGTs from several distinct sources, involving a variety of biochemical activities and metabolic pathways (Table 2). Notably, the nucleotide biosynthesis pathway contains at least two previously published, independently transferred genes from eubacteria. Inosine 5' monophosphate dehydrogenase (IMPDH), an enzyme for purine salvage, was transferred from ε-proteobacteria [32]. Another enzyme involved in pyrimidine salvage, thymidine kinase (TK), is of α or γ-proteobacterial ancestry [25].

Another gene of eubacterial origin identified in *C. parvum *is tryptophan synthetase β subunit (*trpB*). This gene has been identified in both *C. parvum *and *C. hominis*, but not in other apicomplexans. The relationship of *C. parvum trpB *to proteobacterial sequences is well-supported as a monophyletic group by two of the three methods used in our analyses (Figure 4c).

**Figure 5:**
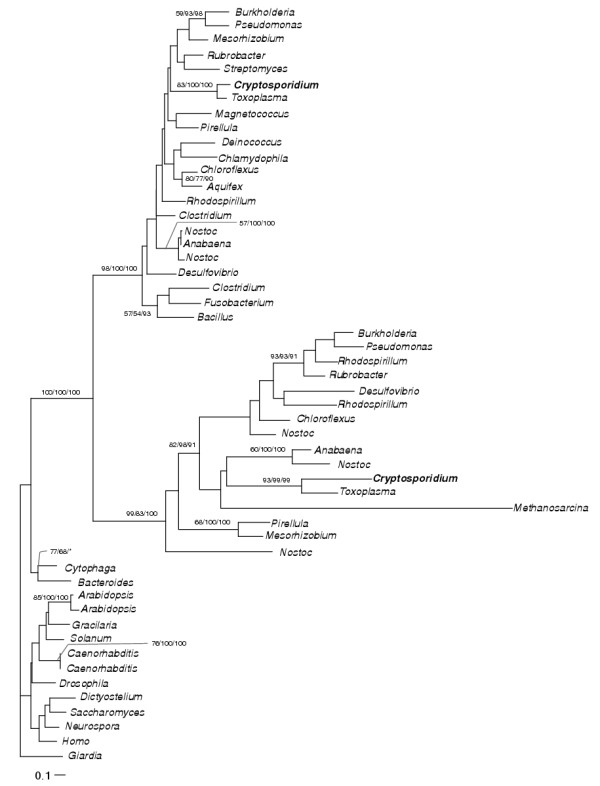
Phylogenetic analyses of 1,4-α-glucan branching enzyme. Numbers above the branches (where space permits) show the puzzle frequency (TREE-PUZZLE) and bootstrap support for both maximum parsimony and neighbor-joining analyses respectively; Asterisks indicate that support for this branch is below 50%. The scale is as indicated. GI accession numbers and alignment are provided in Additional data file 1.

Other HGTs of eubacterial origin include the genes encoding α-amylase and glutamine synthetase and two copies of 1,4-α-glucan branching enzyme, all of which are overwhelmingly similar to eubacterial sequences. α-amylase shows no significant hit to any other apicomplexan or eukaryotic sequence, suggesting a unique HGT from eubacteria to *C. parvum*. Glutamine synthetase is a eubacterial gene found in *C. parvum *and all apicomplexans examined. The eubacterial affinity of the apicomplexan glutamine synthetase is also demonstrated by a well supported (80% with maximum parsimony) monophyletic grouping with eubacterial homologs (data not shown). The eubacterial origin of 1,4-α-glucan branching enzyme is shown in Figure 5. Each copy of the gene is found in a strongly supported monophyletic group of sequences derived only from prokaryotes (including cyanobacteria) and one other apicomplexan organism, *T. gondii*. It is possible that these genes are of plastidic origin and were transferred to the nuclear genome before the divergence of *C. parvum *and *T. gondii*; the phylogenetic analysis provides little direct support for this interpretation, however.

### Mode of acquisition

We examined the transferred genes for evidence of non-independent acquisition, for example, blocks of transferred genes or evidence that genes were acquired together from the same source. Examination of the chromosomal location of the genes listed in Table 2 demonstrates that the genes are currently located on different chromosomes and in most cases do not appear to have been transferred or retained in large blocks. There are two exceptions. The *trpB *gene and the gene for aspartate ammonia ligase are located 4,881 base-pairs (bp) apart on the same strand of a contig for chromosome V; there is no annotated gene between these two genes. Both genes are of eubacterial origin and are not found in other apicomplexan organisms. While it is possible that they have been acquired independently with this positioning, or later came to have this positioning via genome rearrangements, it is interesting to speculate that these genes were acquired together. The origin of *trpB *is proteobacterial. The origin of aspartate ammonia ligase is eubacterial, but not definitively of any particular lineage. In the absence of genome sequences for all organisms, throughout all of time, exact donors are extremely difficult to assess and inferences must be drawn from sequences that appear to be closely related to the actual donor.

In the second case, *C. parvum *encodes two genes for 1,4-α-glucan branching enzymes. Both are eubacterial in origin and both are located on chromosome VI, although not close together. They are approximately 110 kb apart and many intervening genes are present. The evidence that these genes were acquired together comes from the phylogenetic analysis presented in Figure 5. The duplication that gave rise to the two 1,4-α-glucan branching enzymes is old, and is well supported by the tree shown in Figure 5. A number of eubacteria (11), including cyanobacteria, contain this duplication. The 1,4-α-glucan branching enzymes of *C. parvum *and *T. gondii *represent one copy each of this ancient duplication. This suggests that the ancestor of *C. parvum *and *T. gondii *acquired the genes after they had duplicated and diverged in eubacteria.

### Expression of transferred genes

**Figure 6:**
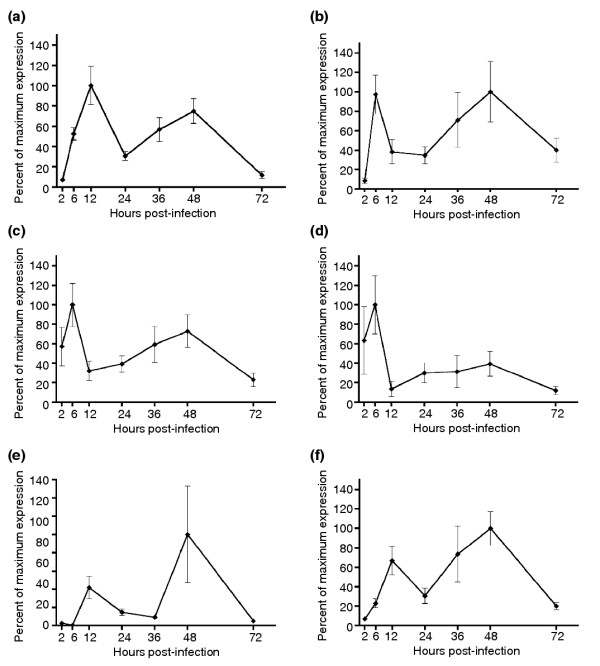
Expression profiles of select genes in *C. parvum*-infected HCT-8 cells. The expression level of each gene is calculated as the ratio of its RT-PCR product to that of *C. parvum *18s rRNA. **(a) **glucose-6-phospate isomerase; **(b) **leucine aminopeptidase; **(c) **pteridine transporter (BT-1); **(d) **tyrosyl-tRNA synthetase; **(e) **calcium-dependent protein kinase; **(f) **dihydrofolate reductase-thymidine synthetase (DHFR-TS). The genes examined in (a-c, e) represent transferred genes of different origins; (d, f) represent non-transferred references. Error bars show the standard deviation of the mean of six independent time-course experiments.

Each of the genes identified in the above analyses (Table 2) appears to be an intact non-pseudogene, suggesting that these genes are functional. To verify the functional status of several of the transferred genes, semi-quantitative reverse transcription PCR (RT-PCR) was carried out to characterize their developmental expression profile. Each of the RNA samples from *C. parvum*-infected HCT-8 cells was shown to be free of contaminating *C. parvum *genomic DNA by the lack of amplification product from a reverse transcriptase reaction sham control. RT-PCR detected no signals in cDNA samples from mock-infected HCT-8 cells. On the other hand, RT-PCR product signals were detected in the *C. parvum*-infected cells of six independent time-course experiments for each of the genes examined (those for G6PI, leucine aminopeptidase, BT-1, a calcium-dependent protein kinase, tyrosyl-tRNA synthetase, dihydrofolate reductase- thymidine synthetase (DHFR-TS)). The expression profiles of the acquired genes show that they are regulated and differentially expressed throughout the life cycle of *C. parvum *in patterns characteristic of other non-transferred genes (Figure 6).

A small published collection of 567 EST sequences for *C. parvum *is also available. These ESTs were searched with each of the 31 candidate genes surviving the phylogenomic screen. Three genes - aspartate ammonia ligase, BT-1 and lactate dehydrogenase - are expressed, as confirmed by the presence of an EST (Table 2).

## Discussion

A genome-wide search for intracellular and horizontal gene transfers in *C. parvum *was carried out. We systematically determined the evolutionary origins of genes in the genome using phylogenetic approaches, and further confirmed the existence and expression of putatively transferred genes with laboratory experiments. The methodology adopted in this study provides a broad picture of the extent and the importance of gene transfer in apicomplexan evolution.

The identification of gene transfers is often subject to errors introduced by methodology, data quality and taxonomic sampling. The phylogenetic approach adopted in this study is preferable to similarity searches [33,34] but several factors, including long-branch attraction, mutational saturation, lineage-specific gene loss and acquisition, and incorrect identification of orthologs, can distort the topology of a gene tree [35,36]. Incompleteness in the taxonomic record may also lead to false positives for IGT and HGT identification. In our study, we have attempted to alleviate these factors, as best as is possible, by sampling the GenBank nonredundant protein database, dbEST and organism-specific databases and by using several phylogenetic methods. Still, these issues remain a concern for this study as the taxonomic diversity of unicellular eukaryotes is vastly undersampled and studies are almost entirely skewed towards parasitic organisms.

The published analysis of the *C. parvum *genome sequence identified 14 bacteria-like and 15 plant-like genes based on similarity searches [7]. Six of these bacterial-like and three plant-like genes were also identified as probable transferred genes in the phylogenomic analyses presented here. We have examined the fate of genes identified by one analysis and not the other to uncover the origin of the discrepancy. First, methodology is the single largest contributing factor. Genes with bacterial-like or plant-like BLAST similarities which, from the phylogenetic analyses, do not appear to be transfers were caused by the fact that PyPhy was unable to generate trees due to an insufficient number of significant hits in the database, or because of the stringent coverage length and similarity requirements adopted in this analysis. Only seven of the previously identified 15 plant-like and 11 of 14 eubacterial-like genes survived the predefined criteria for tree construction. Second, subsequent phylogenetic analyses including additional sequences from non-GenBank databases failed to provide sufficient evidence or significant support for either plant or eubacterial ancestry. Third, searches of dbEST and other organism-specific databases yielded other non-plant or non-eubacterial organisms as nearest neighbors, thus removing the possibility of a transfer.

The limitations of similarity searches and incomplete taxonomic sampling are well evidenced in our phylogenomic analyses. From similarity searches, *C. parvum*, like *P. falciparum *[26], is more similar to the plants *Arabidopsis *and *Oryza *than to any other single organism. Almost 800 predicted genes have best non-apicomplexan BLAST hits of at least 10^-7 ^to plants and eubacteria (Table 1). Yet only 31 can be inferred to be transferred genes at this time with the datasets and methodology available (Table 2). In many cases (for example, phosphoglucomutase) the *C. parvum *gene groups phylogenetically with plant and bacterial homologs, but with only modest support. In other cases, such as pyruvate kinase and the bi-functional dehydrogenase enzyme (AdhE), gene trees obtained from automated PyPhy analyses indicate a strong monophyletic grouping of the *C. parvum *gene with plant or eubacterial homologs, but this topology disappears when sequences from other unicellular eukaryotes, such as *Dictyostelium*, *Entamoeba *and *Trichomonas *are included in the analysis (data not shown).

The list of genes in Table 2 should be considered a current best estimate of the IGTs and HGTs in *C. parvum *instead of a definitive list. As genomic data are obtained from a greater diversity of unicellular eukaryotes and eubacteria, phylogenetic analyses of nearest neighbors are likely to change.

### Did *Cryptosporidium* contain an endosymbiont or plastid organelle?

The *C. parvum *sequences of cyanobacterial and algal origin reported here had to enter the genome at some point during its evolution. Formal possibilities include vertical inheritance from a plastid-containing chromalveolate ancestor, HGT from the cyanobacterial and algal sources (or from a secondary source such as a plastid-containing apicomplexan), or IGT from an endosymbiont/plastid organelle during evolution, followed by loss of the source. *Cryptosporidium *does not harbor an apicoplast organelle or any trace of a plastid genome [7]; thus an IGT scenario would necessitate loss of the organelle in *Cryptosporidium *or the lineage giving rise to it. The exact position of *C. parvum *on the tree of life has been debated, with developmental and morphological considerations placing it within the Apicomplexa, and molecular analyses locating it in various positions, both within and outside the Apicomplexa [3], but primarily within. If we assume that *C. parvum *is an apicomplexan, and if the secondary endosymbiosis which is believed to have given rise to the apicoplast occurred before the formation of the Apicomplexa, as has been suggested [18], *C. parvum *would have evolved from a plastid-containing lineage and would be expected to harbor traces of this relationship in its nuclear genome. Genes of likely cyanobacterial and algal/plant origin are detected in the nuclear genome of *C. parvum *(Table 2) and thus IGT followed by organelle loss cannot be ruled out.

What about other interpretations? While it is formally possible that these genes were acquired independently via HGT in *C. parvum*, their shared presence in other alveolates (including the non-plastidic ciliate *Tetrahymena*) provides the best evidence against this scenario as multiple independent transfers would be required and so far there is no evidence for intra-alveolate gene transfer. Vertical inheritance is more difficult to address as it involves distinguishing between genes acquired via IGT from a primary endosymbiotic event versus a secondary endosymbioic event. Our data, especially the analysis of G6PI and BT-1 are consistent with both primary and secondary endosymbioses, provided that the secondary endosymbiosis is pre-alveolate in origin. As more genome data become available and flanking genes can be examined for each gene in a larger context, positional information will be informative in distinguishing among the alternatives.

The plastidic nature of some genes is particularly apparent. There is a shared indel among leucine aminopeptidase protein sequences in apicomplexans, cyanobacteria and plant chloroplast precursors (Figure 3). The *C. parvum *leucine aminopeptidase does contain an amino-terminal extension of approximately 85-65 amino acids (depending on the alignment) relative to bacterial homologs, but this extension does not contain a signal sequence. The extension in *P. falciparum *is 85 amino acids and the protein is believed to be targeted to the apicoplast [26,37]. No similarity is detected between the *C. parvum *and *P. falciparum *amino-terminal extensions (data not shown).

Other genes were less informative in this analysis. Among these, aldolase was reported in both *P. falciparum *[38] and the kinetoplastid parasite *Trypanosoma *[38] as a plant-like gene. The protein sequences of aldolase are similar in *C. parvum *and *P. falciparum*, with an identity of 60%. In our phylogenetic analyses, *C. parvum *clearly forms a monophyletic group with *Plasmodium*, *Toxoplasma *and *Eimeria*. This branch groups with *Dictyostelium*, Kinetoplastida and cyanobacterial lineages, but bootstrap support is not significant. The sister group to the above organisms are the plants and additional cyanobacteria, but again with no bootstrap support (see Additional data file 1 for phylogenetic tree). Another gene, enolase, contains two indels shared between land plants and apicomplexans (including *C. parvum*) and was suggested to be a plant-like gene [29], but alternative explanations exist [39].

The biochemical activity of the polyamine biosynthetic enzyme arginine decarboxylase (ADC), which is typically found in plants and bacteria, was previously reported in *C. parvum *[19]. However, we were unable to confirm its presence by similarity searches of the two *Cryptosporidium *genome sequences deposited in CryptoDB using plant (*Cucumis sativa*, GenBank accession number AAP36992), cyanobacterial (*Nostoc *sp., NP-487441; *Synechocystis *sp., NP-439907) and other bacterial (*Yersinia pestis*, NP-404547) homologs.

### A plethora of prokaryotic genes

Several HGTs from bacteria have been reported previously in *C. parvum *[25,32,40]. We detected many more in our screen of the completed *C. parvum *genome sequence (Table 2). In most cases, the exact donors of these transferred genes were difficult to determine. However, for those genes whose donors could be more reliably inferred (Table 2), several appear to be from different sources and hence represent independent transfer events. In one compelling case, both the *trpB *and aspartate ammonia ligase genes are located 4,881 bp apart on the same strand of a contig for chromosome V and there is no gene separating them. Both genes are of eubacterial origin and neither gene is detected in other apicomplexans. In addition, the aspartate ammonia ligase gene is expressed, as evidenced by an EST. In another case, copies of a 1,4-α-glucan branching enzyme gene duplication pair that is present in many eubacteria, were detected on the same chromosome in *C. parvum*. *C. parvum *also contains many transferred genes from distinct eubacterial sources that are not present in other apicomplexans (for example, IMPDH, TK (thymidine kinase), *trpB *and the gene for aspartate ammonia ligase).

The endosymbiotic event that gave rise to the mitochondrion occurred very early in eukaryotic evolution and is associated with significant IGT. However, most of these transfer events happened long before the evolutionary time window we explored in this study [41]. Many IGTs from the mitochondrial genome that have been retained are almost universally present in eukaryotes (including *C. parvum *which does not contain a typical mitochondrion [7,42-44]) and thus would not be detected in a PyPhy screen since the 'nearest phylogenetic neighbor' on the tree would be taxonomically correct and not appear as a relationship indicative of a gene transfer.

### The impact of gene transfers on host evolution

Gene transfer is an important evolutionary force [21,22,45,46]. Several of the transferred genes identified in *C. parvum *are known to be expressed. IMPDH has been shown to be essential in *C. parvum *purine metabolism [32] and TK has been shown to be functional in pyrimidine salvage [25]. It is not yet clear whether these genes were acquired independently in this lineage, or have been lost from the rest of the apicomplexan lineage, or whether both these have happened. However, it is clear that their presence has facilitated the remodeling of nucleotide biosynthesis. *C. parvum *no longer possesses the ability to synthesize nucleotides; instead it relies entirely on salvage.

Many apicoplast and algal nuclear genes have been transferred to the host nuclear genome, where they were subsequently translated in the cytosol and their proteins targeted to the apicoplast organelle. However, as there is no apicoplast in *C. parvum*, acquired plastidic proteins are theoretically destined to go elsewhere. In the absence of an apicoplast, it is tempting to suspect that plastid-targeted proteins would have been lost, or would be detected as pseudogenes. No identifiable pseudogenes were detected and at least one gene is still viable. The *C. parvum *leucine aminopeptidase, which still contains an amino-terminal extension (without a signal peptide), is intact and is expressed, as shown in Figure 6. None of the cyanobacterial/algal genes identified in our study contains a canonical presequence for apicoplast targeting. One exception to this is phosphoglucomutase, a gene not present in Table 2 because of its poorly supported relationships in phylogenetic analyses. This gene exists in two copies as a tandem duplication in the *C. parvum *genome. One copy has a long amino-terminal extension (97 amino acids) beginning with a signal peptide. The extension does not contain characteristics of a transit peptide. Expression of a fluorescent reporter construct containing this extension in a related parasite, *T. gondii*, did not reveal apicoplast targeting but instead secretion via dense granules (see Additional data file 1). Exactly how and where intracellularly transferred genes (especially those that normally target the apicoplast) have become incorporated into other metabolic processes remains a fertile area for exploration.

## Conclusions

*Cryptosporidium *is the recipient of a large number (31) of transferred genes, many of which are not shared by other apicomplexan parasites. The genes have been acquired from several different sources including α-, β-, and ε-proteobacteria, cyanobacteria, algae/plants and possibly the Archaea. We have described two cases of two genes that appear to have been acquired together from a eubacterial source: *trpB *and the aspartate ammonia ligase gene are located within 5 kb of each other, while the two copies of 1,4-α-glucan branching enzyme represent copies of an ancient gene duplication also observed in cyanobacteria.

Once thought to be a relatively rare event, reports of gene transfers in eukaryotes are increasingly common. The abundance of available eukaryotic genome sequence is providing the material for analyses that were not possible only a few years ago. Analysis of the *Arabidopsis *genome [47] has revealed potentially thousands of genes that were transferred intracellularly. HGTs are still a relatively rare class of genes among multicellular eukaryotes, most probably because of the segregation of the germ line. By definition, unicellular eukaryotes do not have a separate germ line and are obligated to tolerate the acquisition of foreign genes if they are to survive. Among unicellular eukaryotes, there are now many reports of HGTs: *Giardia *[48,49], *Trypanosoma *[38], *Entamoeba *[21,49], *Euglena *[50], *Cryptosporidium *[25,32,40] and other apicomplexans [51].

As discussed earlier, genes transferred from distant phylogenetic sources such as eubacteria could be potential therapeutic targets. In apicomplexans, transferred genes are already some of the most promising targets of anti-parasitic drugs and vaccines [7,25,52]. We have shown that several transferred genes are differentially expressed in the *C. parvum *genome, and in two cases (IMPDH and TK), the transferred genes have been shown to be functional [25,32]. The successful integration, expression and survival of transferred genes in the *Cryptosporidium *genome has changed the genetic and metabolic repertoire of the parasite.

## Materials and methods

### Cryptosporidium sequence sources

Genomic sequences for *C. parvum *and *C. hominis *were downloaded from CryptoDB [53]. Genes were predicted for the completed *C. parvum *(IOWA) sequence as previously described using the Glimmer program [54] trained on *Cryptosporidium *coding sequences [52]. A few predicted genes that demonstrated apparent sequence incompleteness were reconstructed from genomic sequence by comparison with apicomplexan orthologs. The predicted protein encoding data set contained 5,519 sequences. A comparison of this gene set to the published annotation revealed that the Glimmer-predicted gene set contained all but 40 of the 3,396 annotated protein-encoding sequences deposited in GenBank. These 40 were added to our dataset and analyzed. Glimmer does not predict introns and some introns are present in the genome [7,20]; thus our gene count is artificially inflated. Likewise, the official *C. parvum *annotation did not consider ORFs of less than 100 amino acids that did not have significant BLAST hits and thus may be a slight underestimate [7].

### Database creation

An internal database (ApiDB) containing all available apicomplexan sequence data was created [25]. A second BLAST-searchable database, PyPhynr, was constructed that included SwissProt, TrEMBL and TrEMBL_new, as released in August 2003, predicted genes from *C. parvum*, ORFs of more than 120 amino acids from *Theileria annulata*, and more than 75 amino acids from consensus ESTs for several apicomplexan organisms. Genomic sequences for *T. gondii *(8x coverage) and clustered ESTs were downloaded from ToxoDB [55,56]. Genomic data were provided by The Institute for Genomic Research (TIGR), and by the Sanger Institute. EST sequences were generated by Washington University. In addition, this study used sequence data from several general and species-specific databases. Specifically, the NCBI GenBank nr and dbEST were downloaded [57] and extensively searched. To provide taxonomic completeness, additional genes were obtained via searches of additional databases including: *Entamoeba histolytica *[58], *D. discoideum *[59], the kinetoplastids *Leishmania major *[59], *T. brucei *[59], *T. cruzi *[60], and a ciliate *Tetrahymena thermophila *[61]. Sequence data for *T. annulata*, *E. histolytica*, *D. discoideum*, *L. major *and *T. brucei *were produced by the Pathogen Sequencing Unit of the Sanger Institute and can be obtained from [62]. Preliminary sequence data for *T. thermophila *was obtained from TIGR and can be accessed at [63].

### Phylogenomic analyses and similarity searches

The source code of the phylogenomic software PyPhy [27] was kindly provided by Thomas Sicheritz-Ponten and modified to include analyses of eukaryotic groups, and changes to improve functionality [51]. For initial phylogenomic analyses, a BLAST cutoff of 60% sequence length coverage and 50% sequence similarity was adopted and the neighbor-joining program of PAUP 4.0b10 for Unix [64] was used. A detailed description of our phylogenomic pipeline and PyPhy implementation are described [51] and outlined in Figure 1.

Output gene trees with phylogenetic connections (that is, the nearest non-self neighbors at a distinct taxonomic rank) [27] to prokaryotes and algae-related groups were manually inspected. As the trees are unrooted, several factors were considered in the screen for candidate transferred genes. If the *C. parvum *gene does not form a monophyletic group with prokaryotic or plant-related taxa regardless of rooting, the subject gene was eliminated from further consideration. If the topology of the gene tree is consistent with a phylogenetic anomaly caused by gene transfer, but may also be interpreted differently if the tree is rooted otherwise, it was removed from consideration at this time. If the top hits of both nr and dbEST database searches are predominantly non-plant eukaryotes, and the topology of the tree was poor, the subject gene was considered an unlikely candidate. Finally, all 551 protein sequences predicted to be NEAPs in the malarial parasite *P. falciparum *[26] were used to search the *C. parvum *genome and the results were screened using a BLAST cutoff E-value of 10^-5 ^and a length coverage of 50%. Sequences identified by these searches were added to the candidate list (if not already present) for manual phylogenetic analyses to verify their likely origins. It should be noted that all trees were screened for the existence of a particular phylogenetic relationship. In some cases the proteins utilized to generate a particular tree are capable of resolving relationships among many branches of the tree of life, and in others they are not. Despite these differences in resolving power, the proteins which survive our phylogenetic screen and subsequent detailed analyses described below exhibit significant support for the branches of the tree in which we are interested. Similar procedures were used to characterize the complement of nuclear-encoded genes of plastid origin in the *Arabidopsis *genome [65]. BLAST searches were performed on GenBank releases 138-140 [57].

Detailed phylogenetic analyses of candidate genes identified by phylogenomic screening: candidate genes surviving the PyPhy phylogenomic screen were reanalyzed with careful attention to taxonomic completeness, including representative species from major prokaryotic and eukaryotic lineages when necessary and possible. New multiple sequence alignments were created with ClustalX [66], followed by manual refinement. Only unambiguously aligned sequence segments were used for subsequent analyses (see Additional data file 1). Phylogenetic analyses were performed with a maximum likelihood method using TREE-PUZZLE version 5.1 for Unix [67], a distance method using the program neighbor of PHYLIP version 3.6a package [68], and a maximum parsimony method with random stepwise addition using PAUP* 4.0b10 [64]. Bootstrap support was estimated using 1,000 replicates for both parsimony and distance analyses and quartet puzzling values were obtained using 10,000 puzzling steps for maximum likelihood analyses. Distance calculation used the Jones-Taylor-Thornton (JTT) substitution matrix [69], and site-substitution variation was modeled with a gamma-distribution whose shape parameter was estimated from the data. For maximum likelihood analyses, a mixed model of eight gamma-distributed rates and one invariable rate was used to calculate the pairwise maximum likelihood distances. The unrooted trees presented in Figures 4 and 5 were drawn by supplying TREE-PUZZLE with the maximum parsimony tree and using TREE-PUZZLE distances as described above to calculate the branch lengths. The trees were visualized and prepared for publication with TreeView X Version 0.4.1 [70].

### Genomic Southern analysis

*C. parvum *(IOWA) oocysts (10^8^) were obtained from the Sterling Parasitology Laboratory at the University of Arizona and were lysed using a freeze/thaw method. Genomic DNA was purified using the DNeasy Tissue Kit (Qiagen). Genomic DNA (5 μg) was restricted with *Bam*H1 and *Eco*R1 respectively and electrophoresed on a 0.8% gel in 1x TAE buffer, transferred to a positively charged nylon membrane (Bio-Rad), and fixed using a UVP crosslinker set at 125 mJ as described in [71]. *C. parvum *genomic DNA for the probes (700-1,500 bp) was amplified by PCR (see Additional data file 1).

### Semi-quantitative reverse transcription-PCR

Sterilized *C. parvum *(IOWA isolate) oocysts were used to infect confluent human adenocarcinoma cell monolayers at a concentration of one oocyst per cell as previously described [72]. Total RNA was prepared from mock-infected and *C. parvum*-infected HCT-8 cultures at 2, 6, 12, 24, 36, 48 and 72 h post-inoculation by directly lysing the cells with 4 ml TRIzol reagent (GIBCO-BRL/Life Technologies). Purified RNA was resuspended in RNAse-free water and the integrity of the samples was confirmed by gel electrophoresis.

Primers specific for several transferred genes identified in the study were designed (see Additional data file 1) and a semi-quantitative RT-PCR analysis was carried out as previously described [72]. Primers specific for *C. parvum *18S rRNA were used to normalize the amount of cDNA product of the candidate gene to that of *C. parvum *rRNA in the same sample. PCR products were separated on a 4% non-denaturing polyacrylamide gel and signals from specific products were captured and quantified using a phosphorimaging system (Molecular Dynamics). The expression level of each gene at each time point was calculated as the ratio of its RT-PCR product signal to that of the *C. parvum *18S rRNA. Six independent time-course experiments were used in the analysis.

## Additional data files

Additional data is provided with the online version of this paper, consisting of a PDF file (Additional data file 1) containing: materials and methods for genomic Southern analysis; the amino-acid sequences of genes listed in Table 2; accession numbers for sequences used in Figure 4; accession numbers for sequences used in Figure 5; expression of *C. parvum *phosphoglucomutase in *T. gondii*; table of primers used for RT-PCR experiments; phylogenetic tree of aldolase; alignment files for phylogenetic analyses in Figure 4; and the alignment of 1,4-α-glucan branching enzyme sequences used in Figure 5.

## Supplementary Material

Additional data file 1 This file contains the materials and methods for genomic Southern analysis; the amino-acid sequences of genes listed in Table 2; accession numbers for sequences used in Figure 4; accession numbers for sequences used in Figure 5; expression of *C. parvum *phosphoglucomutase in *T. gondii*; table of primers used for RT-PCR experiments; phylogenetic tree of aldolase; alignment files for phylogenetic analyses in Figure 4; and the alignment of 1,4-α-glucan branching enzyme sequences used in Figure 5 Click here for additional data file

## Figures and Tables

**Table 1:** Distribution of best non-apicomplexan BLAST hits in searches of the GenBank non-redundant protein database

Category	E < 10^-3^	E < 10^-7^
Plants	670	588
Algae	30	21
Non-cyanobacterial eubacteria	188	117
Cyanobacteria	22	16
Archaea	26	11
Total	936	783

**Table 2:** Genes of algal or eubacterial origin in *C. parvum*

Putative gene name	Accession	Location	Expression	Indel	Putative origin	Putative function
Lactate dehydrogenase*	AAG17668	VII	EST	+	α-proteobacteria	Oxidoreductase
Malate dehydrogenase*	AAP87358	VII		+	α-proteobacteria	Oxidoreductase
Thymidine kinase	AAS47699	V	Assay	+	α/γ-proteobacteria	Kinase; nucleotide metabolism
Hypothetical protein A^†^	EAK88787	II			γ-proteobacteria	Unknown
Inosine 5' monophosphate dehydrogenase	AAL83208	VI	Assay	+	ε-proteobacteria	Purine nucleotide biosynthesis
Tryptophan synthetase β chain	EAK87294	V			Proteobacteria	Amino acid biosynthesis
1,4-α-glucan branching enzyme	CAD98370	VI			Eubacteria	Carbohydrate metabolism
1,4-α-glucan branching enzyme	CAD98416	VI			Eubacteria	Carbohydrate metabolism
Acetyltransferase	EAK87438	VIII			Eubacteria	Unknown
α-amylase	EAK88222	V			Eubacteria	Carbohydrate metabolism
DNA-3-methyladenine glycosylase	EAK89739	VIII			Eubacteria	DNA repair
RNA methyltransferase	AY599068	II			Eubacteria	RNA processing and modification
Peroxiredoxin	AY599067	IV			Eubacteria	Oxidoreductase; antioxidant
Glycerophosphodiester phosphodiesterase	AY599066	IV			Eubacteria	Phosphoric ester hydrolase
ATPase of the AAA class	EAK88388	I			Eubacteria	Post-translational modification
Alcohol dehydrogenase	EAK89684	VIII			Eubacteria	Energy production and conversion
Aminopeptidase N	AAK53986	VIII			Eubacteria	Peptide hydrolase
Glutamine synthetase	CAD98273	VI		+	Eubacteria	Amino acid biosynthesis
Conserved hypothetical protein B	CAD98502	VI			Eubacteria	Unknown
Aspartate-ammonia ligase^†^	EAK87293	V	EST		Eubacteria	Amino acid biosynthesis
Asparaginyl tRNA synthetase^†^	EAK87485	VIII			Eubacteria	Translation
Glutamine cyclotransferase^†^	EAK88499	I			Eubacteria	Amido transferase
Leucine aminopeptidase	EAK88215	V	RT-PCR	+	Cyanobacteria	Hydrolase
Biopteridine transporter (BT-1)	CAD98492	VI	RT-PCR /EST	+	Cyanobacteria	Biopterine transport
Hypothetical protein C^† ^(possible Zn-dependent metalloprotease)	EAK89015	III			Archaea	Putative protease
Superoxide dismutase^†^	AY599065	V			Eubacteria /archaea	Oxidoreductase; antioxidant
Glucose-6-phosphate isomerase	EAK88696	II	RT-PCR	+	Algae/plants	Carbohydrate metabolism
Uridine kinase/uracil phosphoribosyltransferase^†^	AAS47700	VIII			Algae/plants	Nucleotide salvage metabolism
Calcium-dependent protein kinases* ^†^	AAS47705	II	RT-PCR		Algae/plants	Kinase; cell signal transduction
	AAS47706	II				
	AAS47707	VII				

*Genes that have been derived from a duplication following transfer; ^†^transferred genes that have less support. GenBank accession numbers are as indicated. Locations are given as chromosome number. The expression status for each gene is indicated by method: EST, RT-PCR or assay. Only 567 EST sequences exist for *C. parvum*. A + in the indel colum indicates the presence of a shared insertion/deletion between the *C. parvum *sequence and other sequences from organisms identified in the putative origin column.
