# Supplementary material for: Phylogenomic evidence supports past endosymbiosis, intracellular and horizontal gene transfer in Cryptosporidium parvum
Source: Genome Biol. 2004 Oct 19;5(11):R88. doi: 10.1186/gb-2004-5-11-r88 (PMC545779; doi:10.1186/gb-2004-5-11-r88)
Supplement: Additional data file 1 — This file contains the materials and methods for genomic Southern analysis; the amino-acid sequences of genes listed in Table 2; accession numbers for sequences used in Figure 4; accession numbers for sequences used in Figure 5; expression of C. parvum phosphoglucomutase in T. gondii; table of primers used for RT-PCR experiments; phylogenetic tree of aldolase; alignment files for phylogenetic analyses in Figure 4; and the alignment of 1,4-α-glucan branching enzyme sequences used in Figure 5 [file gb-2004-5-11-r88-s1.pdf]

DESCRIPTION of ADDITIONAL DATA

Supporting Information Index

1) **Materials and methods for genomic Southern analysis, Figure 1**
2) **Amino acid sequences of genes listed in Table 2**
3) **Accession numbers for sequences used in Figure 4**
4) **Accession numbers for sequences used in Figure 5**
5) **Expression of *C. parvum* phosphoglucomutase in *T. gondii***
6) **Table of primers used for RT-PCR experiments, Figure 6**
7) **Phylogenetic tree of aldolase**
8) **Alignment files for phylogenetic trees in Figure 4**
9) **Alignment of 1,4-alpha-glucan branching enzyme sequences used in Figure 5**

1) Genomic Southern Materials and Methods

Primers used to generate *C. parvum* probes for genomic Southern analysis.

Name	Size	Forward Primer	Reverse Primer
aminopeptidase N	1040	5′ATGGGCAATTTATTCGGAAAATGTG	5′ATTACCTGTGTAGTTATGAAAGTATTC
glucose-6-phosphate isomerase	1040	5′ATGCCAGAACTTTATGAACTTCC	5′GCCTGACAATATGGGAGAACTGCA
glutamine synthetase	1443	5′ATGAATAACTTCAAAGTTTCC	5′GTCATAATACAGTTCAAACTC
leucine aminopeptidase	1572	5′ATGGTAAATCCAATACATCCAG	5′AGTAACTAAAGTCTTGACGCC
pteridine transporter (BT-1)	699	5′GATAAAAAACAGACAGAAGATCTC	5′CAACCTAAAAAAATGAACTTG

The PCR products amplified to be used as probes were purified using a Q-Biogene Gene Clean II Kit and probe DNA was labeled using the Invitrogen Random Primers DNA Labeling System and P32 labeled ATP (Amersham). Hybridization was performed at 42°C in a 50% formamide, 10% 50x Denharts, 25% 20xSSC, 0.5% 20% SDS, 1% boiled salmon sperm hybridizing solution. Five post hybridization washes were applied 15 min each at 42°C, from least to most stringent: 2x SSC, 0.5% SDS; 2xSSC, 0.1% SDS; 1x SSC, 0.1% SDS; 0.5xSSC, 0.1%SDS; 0.1xSSC, 0.1%SDS. Kodak Biomax MS film was used to capture the images.

Gene Name	Expected size (bp) BamHI	Expected size (bp) EcoRI
aminopeptidase N	6118, 5245	NA
glucose-6-phosphate isomerase	21848	NA
glutamine synthetase	NA	2639, 808,83
leucine aminopeptidase	6453	NA
pteridine transporter (BT-1)	16,603	NA

2) Amino acid sequences of genes listed in Table 2

>222_2 tryptophan synthetase beta subunit
MTDNTYLNNFPDSNGYFGKYGGKYVPEVINNAMKEIEDAYNKISKSEDFINELKKIRKEFQGRPTPIYYAK
NLTKKYGGAEIYLKREDLNHTGAHKLNHCMGEALLAKYMGKKKLIAETGAGQHGVALATAAAYFGLECEIH
MGEVDVKKEYPNVIRMKILGAKVVCVEFGDKTLKEAVDSAFEAYIKDIDNTFYAIGSVVGPHPFPKMVRDF
QSIVGIESREQFLEMNGDGILPDIVTACVGGGSNAMGIFSGFISDKQVELICVEPLGKGNKIGEHAASITY
GSEGIMHGFNSIMLKDEEGNPSKVHSIASGLDYPSVGPEIAYLNSIGRTKTVCITDQEAINGFFELSRTEG
IIPAIESSHAIGYVLKIAKEMKGKKILINLSGRGDKDLDFVVQNYGYGNN

>239_15 acetyltransferase EAK87438
MISSFEVRKATIDDYFELRNLICDVTRCTETLSREQAEERFRYNTYHPYCLVDTENGRIVGYAGFYIIPHL
GRKNDSRIEHVIISKEYRNRGLGRLLCKQIIEDAKNKFNCGRIDLTVESHIAKKLYSSLEFEKVNTEVMRN
SFLDLTPKSD

>246_15 alpha-amylase
MVINGNEWYKNCNWYHIYPIGLCGVERINSDKIVRVNKLEELGSKSWIDHLKNLNIGGIYIGPVFESEAHG
YDTTDLLSIDKRLGSNEDFKNLVKIYHSNGIKVIIDAVFNHVGRNFFAFNDIKINGKHSKYCDWFKGLDFS
KKSQKGDNFTYTPWEGYYELVTLNHENYEVKKYLFDAVEFWFKEYEIDGLRLDAADCISIEFWRIFRDFCK
TKFGENFALLAEIIHGDQRIWVRPNPSASIKYEYTNQPFDGVTNYVLWDAIWKSHRFNNLELLANVISQQN
DLFSSGWMYNFVDNHDVTRIASQIEIEDDLLTVYIILFMLNGSPSIYYGSEFRFKGVKGKGRRADFQLRPK
LTIKDLEWLNSDKNDGFLVLIKFISQIRGHPIIGKILTKGNYSLLLNTKTLLVFERRIKEEFIFVAINIDT
KSVEDFEVDWKEKDGQWRDILSPSDTYQSKKGKIKINVLSNWAIIISSCIPKTSYNQYKIKKFKKVLYNIP
TLDRSELIEN

>32398905_Cryptosporidium_parvum 1, 4 glucan branching enzyme
MSTRGVLQKYNMKSLKEQKIDTTLSGKQKTECKVKSSSKFTGEVLITSKNISSPTQSLEKSDSLPVSSSD
NSRIESSVPTAANSPTSYSSSMASIPDERGDIYEALDRVPDDGKILDHDLHLLNKALHPEPFGVLGYQKF
GDDGQYEYYVVRAWVRNARRIQIKAIDYSFSSIGTNNTPVEMEQRYIDGNPSWMFEKAFKTLKEKIPTCR
THFCGYDENLENSNIENKRIGNQSNKDDYSTDKFSDVGSEANNKVNKNTIVSKKKEITADCNSIVEKTYS
NVIVNTLNKSKTNLVPNKESHKGNEMPKLELNQSNLMSSNIPRNKNIRSEACSSDEACNCYVHDNINSQE
CVRKLYYELLVEYNGDDSGKVYAIRDTYSFGLLLSNGDMELFQSGSCWHVDNILGSHIIEYNGVKGVRFS
VWAPHAKYVRVVGDWNSWDGRVNPMRFRHGVGIWELFIPHLGPGEKYGYEIHSQSNDVFVKIDPYSQEYE
VPPRYASIISACDDSYKDETDRFRWEDQEWIKRREQLGSKGEMRRQPMSIYEVHLPSWMRRENGDYLGYR
EIAERLVEHVKNLNFTHVEFLPLAQHPFEGSWGYQVTGQYAPYSRLGTPDDFKYLVNELHKAKIGVFIDF
VPAHFCKDAWGLVYYDGTPTYEYGDPREGEHKQWGTAVFNFRRNEVRSFLLGAAYHWLRRYHIDGLRIDA
VSSMLYRNYLRPNGEWIPNEFGGDANLEAVSLLQELNWVIHKEFPGVFTMAEESTAWQGVTHKDGGLGFD
AKWDLGWMNDTLSYLYTPPDNKSSKHNKLTFRGLYMSHEHWVLPLSHDEVVNGKGSLLDKCGFTGAPYMD
RIRTLKTLFGYQVGMPGRPLLFQGAEIAQGREWKENRSVDWHEGEEDVRKKVCIFLSDLLAVYKNNVSLH
AGDDESWNFQWVDCENSQDCIIAFLRKYKEWYNDVVVVCNFSSRRYNHYPIGVPHGKEWLVMLNSDDWKY
GGAMFGPGNNSTVHASHGGRVGWDYCLWIDIPEFSCMYLKPLFNPNENDS

>32398951_Cryptosporidium_parvum 1,4 glucan branching enzyme
MSELRNEKVEWSQIKYEELPNYIKKQEKKMGAMFYKEFGICIFRLWVVHCKNVWLVNSGTNESFLMENDD
EINIKRVIMKNVNMNERYHFLLETSNGEKITRRDPYARYTDYDTDSSYITDTLGNFNKSIREYLKKHEFN
HLIIYELHVESYIRRFSEYFPLQSEAKFSKNPSFFRQIADYGLDNIKDLNFNCLELMPIVEYCGEWGYNP
RLLLSINSHLGSVDDFIYLVQKVHEKDLFIVVDLVLHHGASKLNSLWNFDGYNHQGGIYFEGGGDTGWGA
KFNFHKKEVQDMLYEACRVLLGEYGVDGIRFDSVHNMPNWLLKNITFRLKEEFPGRFLIAEVVPENPNYL
KECGFDSCWIHSSYYDIISQFRNQKTQQEWNSGYSKSLIQGHSGFDSTSQCILTMLGNHDQAGNRCNGGN
PSGDDRIGRYIVDQFGGRQNWDARAYCRLLYSLSCISFGIPMVFMGTENLQGKWWSAKDKAYNYDWGLIK
NNDQVTQQMRKLVKDINLLKTKEKEVFASNYNNFSHFNVNTSNFVVVFIRKSKDKAFLCVVNMDKTEWLE
NNYQVKIDQNIIQSFGRELKQEFNSQSEEYGGWDGSYTSPKKGQIKYSIQDSAENNVSYMLNVPKYSVTI
YRFCNL

>258_55 superoxide dismutase

MSIRFIFNNNSKKLVNKFCRLFSGMPFELPPLPYDKKALEPVISPETLDYHYGKHHAGYVTKLNSLIKSTE
FENETDLMKVIMKSSGPIYNNASQIWNHTFYWSCLRSPSETNKPTSKVSKLIEESFGSFESFKESFTANAT
GHFGSGWIWVVIDPINNQKLKIVQTHDGDNPEKLGLGKPVLTCDVWEHAYYIDYRNNRGSYVDQFFSIINW
DFVESNLS

>261_4 DNA-3-methyladenine glycosidase
MKFNYTERELEVLKLRDEKMKKIIEEIGYIDREYIDDLFQGLFYIIIGQQVSQKAQVSIWNKAKTTLKSID
PETISNYSLEEIRKVGVSLKKATFIKGIAEKIINKEIDLNLLHEKDDEEVCEELTKLNGIGVWSAEMAMIF
CMNRKNVFSFSDIAIKRALKMIYGHKEITKEIFEHYRELFSPYCSIVSLYLWEISNGDYKDKLSKKNVKLY

>265_16 RNA methyltransferase
MDDIKINKDPKDLPEFYLVLSNISKRQNFGTLLRSACGFGVSEVLVVGEKKLMTFGNKGTLPHLSLTQYEN
IDQVVDIIKEKEMDLVGIEISDESRPIYPHPFKRSTAFLLGNEGTGLSQKYIKLCDYLIHIPLYGNGTASL
NVAIAGSIVFHHFAIWAKFTESSKKGAKYIIQDDSNSNNNNNNNNNHNIDISNVKGKIRHYLFPSELGTFFS
F

>266_37 peroxiredoxin
MSTLVRKLAPNFTAEAVMADGSFKKVSLSDYRGKYVVLFFYPLNFTFVCPSEILAFNQAQKDFEKLGVQLL
AVSVDSQYSHAAWRRTPLEQGGIGPVNFPLISDSSHSISKNYGVLLEEEGIALRGLFIIDKEGVVRSEVIY
DLPLGRSVEETLRVIDALQFTETYGEVCPANWKKGQKGMSATHEGVSSYLKDSF

>267_6 hypothetical protein A EAK88787
MKLLGVNSFALRQFVEGYRGSYIPRMSPYEFLRNVNNYIIENNPTLVDGYADFCKHIFIPNFTEAKQSIVK
ITNENEKYIKTGYISRRDEEIPVLSRWFPKDSPPASQLIKSKYLDIILYSKEQCEKESSIMNCCLQDILDD
REKNPDWYIISIKAQNESFEVPMEPITILRNTLIEEGGSGVPLKREKYLESVEFWKEHAIVSS

>268_40 Leucine aminopeptidase
MVNPIHPECIPMSEFNESLKFEFSVLKDKYDESLKGDCLVLCVPNKDIMQSIPSFYESLDTILNGILKDII
ETANQESSLKDENNTIHVNLPPKYPFKYLMLISFGENKKTSPVFFNKMITQMNEFQKKNKNIQSFTVVLGF
DNVVSSDIMYFCTQFPIDNTIEKRFKGNIVGSEPKKPTKKIERIHFVLRSEDTIKDCNSRVLDAQSVSRGL
LFARDLTSAPPNYCDPVNMAKEVISMAKSVGLEGKILQPKECEELKMGAFLAVAQGSKSPAQFVHLTYKPK
GEIKKRIALVGKGITMDTGGYNIKHQMIHFMKGDMGGAAAVFGTALSVGLIKPENIEVHFISAICDNLVSR
DAYLPGCIITASNGKTIEVGNTDAEGRLTLADALVYACNLKVETVIDLATLTGANYRLFEGRYASVLGNND
ELFQMIQTCGKTVYEKFWQLPLDPEFQEMVKSEIADLNNTSEGKAPISTSAAFLCEFVDKNVKYAHIDIAG
CSGMGTFGNGFGVKTLVTIIKELSSKC

>269_185 G6PI
MPELYELPSYSALKEYANKQKCIHLRDLLKNEVRNSCLTVNFGEIFMDFTRQNLDEEGFELLIKLAAESNL
MEKIKLQLKGGIINSTEKRAVLHTALRSKSNIPITLSSGQNVLNDVNEVNRRIFKFANAIRKGELLGSTGK
ILKDVICIGIGGSYLGPEFVYEALRTTQEGFEASMGRRLRFLANVDPIDIRRATEGLHPETTLVIIVSKTF
TTAETILNAKTIKEWLHKALKSETAVSKHLAAVSTNIKATSDFGIPVDHVFGFWDWVGGRFSVCSAVGLVP
LSIHFGANLVQEFLDGCWDMDQHYETAPVSKNLPVLLGLLSVYNSTFMDKSCVAVLPYCQALCKFPAHVQQ
LLMESNGKSSSIDGNMLHDKIKTGAIFFGEPGTNAQHSFYQLLHQGRNTTNCEFIGFAKSQCDSQILGDPI
SNHDELMCNFFAQPDALAIGKTQRELNLEDCPENLIPHKLFQGNRSSISLLLPICSAYYIGQLLALYEHRT
AVEGFILNINSFDQYGVELGKVLAKDIRNIISLKKTNSSPEIIQNKEDKIPGPTRRLIDLYLRVSKSLTN

>271_217 glycerophosphodiester phosphodiesterase
MPGIYLIIFYMMVRAKASLHLVAHRGASYFVPEESLAAYSLAAKHGVNFIECDVVSTKDGVLILRHSPNLS
ESTNIDDLIEFSHLRRTEILYDNNSNDAITGIFSWDLTYEEIKRIKCTHEKKYNNRNPSLNGIYDILTLDE
FLKFSVSGFDGNFPGLYIEIKYPTLHERKGIDNITDKLLDALDRFGINKKTDAVYIQSFEPSNLEYIRARS
EVNLVQLLTRDSENNFVYAIEDVENGGQMKYVTCDIEYISKFANVVSPYKEDLIDRTSTSNNTLPSSSHFV
SKAHELGLIVVPYTFRPESILSYFKDIYEEYDYFIKTLKADGLFTDDSESIMQYVNNTYTRLYE

>272_300 hypothetical protein C EAK89015
MIFLSLIGAFEPFISIDNLKFIVKNLQILTGKEIKFIKHAETNINLVMKGRCSVGNISDLKDYADLKEQSN
PGNHQQISTNSMLEVLLKMKNKFITENSKNQMDSNFFLIGITSLDIFPTNDHEYVFGQSDPEKGIAIISAY
RLYQNNKNLMNSRILKVIMHEYGHLMKLGHCKHVCLMSVICSISELDNRTLYCCENCLNQIREKSSTSESV
KNLFEF

>273_158 ATPase of AAA class
MKPVIGNGHLEETNLEKNLLNKRLFLDKIKSFGNKQTEKEIIDNLDRLFDSFSKDLFTDTLIDLNTVLSSA
ENKAKKFDQHSRESDFLNTDSSSNIYHDDSVIPLLENLGVQVFLNRDNCDSHRNLWDSLGGYQDVKRQIEE
HILLSFKYPDVLDKIVNGTRAQSNSNNRPKLILFEGPPGTGKTTSAKIIGNSIQVPLIYVSLENIVSKWYG
ESETKLAQIFDIAKKFNEGCIIFIDEIDTLASSRDKTFSMHEGSKKILSVLLRKLDGFDTLNSKTLLICAT
NRRRDIDEAFLNRVDTTVLFNLPNENERELIFKQYAKHLTFEERMVLAKMSKKLSGRSIRHVCLEAEREWA
SMILKKKEKGEYQRDEIELPTIEIYKEALKKRVS

>273_362 glutamine cyclotransferase
MFLLGLFLFSCLIIKWNFSLSQDSLHSVQLPNIYSVKILQEFPHYHIPENHIFSENDSSYKYNKNPFTQGL
MFLNSTILIESAGLIEGSFIKLIEFPSMKNLQHKILPKRNWGEGIATLNGHIYQLTWTSRVMFGYSLDLSV
VKRYSIPLTGWGLTSDNESRIWATSGSDQLFELNVPDFDSSDNKVTIKKVIMLRCLGKPMHFVNEMEYIPE
TKTIWGNIFQSQMIVEFSPETGECISIANLKSIYDPSKSTLLEHFDILNDVLNGIAYHPSYQEKKISNLNG
PVLFVTGKRWPRIYKIELTKIPIRARKNEQINESGDFDKYFEFYSSSIENPSSISNEKFEM

>274_159 alcohol dehydrogenase
MLILTPIIYYNMDSFRDIISDLLDSNVGRAILVFSSAVKCQAMRVESMVKKNGIQIEFSELCESDANALST
ILLQMKSYKPDCIIGMGGGHCMDIAKVLRVLYEDPNTTLRSLAMGTNNNDKEKSSKKAMIHRRGSLIKKLV
CIPTTCGSGSEVTSTAVLRNDDGRQMIVSGVAFLPDISVIDSSFILTIPMFVASITGMRALLHGLESYISN
SSNHYSSCMAIQSLRILFNSLTKAVIEKDISLLQEIHKAASISGVAISATDVGLGTILSRSISEVFTLPHG
LIDSIVITQVLNFNIKKSQTVGPLIANLSVELGISNPEDSNSNKIQALLNRIDEIKKTLLLPDSLSSIHSS
LSNFKSKGWFDPIGVKLSVFSQCIEIDNQEFINHVSGSFSISDKETRAYVTEENALRTIPILSKKSVIDNL
NFNTLQMNLDRMISRALSDEAIHTNPATVTKEDLSTILKETWG

>274_402 aminopeptidase N
MGNLFGKCGCRDLTICNLNVLRPAHSQDRIYRKEYKVPNFLIDHVNLDINIKDDVTVVSSVIIMRRNPNSS
FRGDLSLDGDCLKLVSVKLNGVILEKSLYKGYFQPDGPDGKLVISSNLLPNKDQQFTLETVVEIFPDRNTK
NMGLYYSAGVYSTQCESDGFRRITFFLDRPDVMCKFRVRIEGDKTKSPVLLSNGNLLEKGDVQGSENRHFA
IFVDPFPKPCYLFAVVAGVLGRLEDKFITKSGKTVRLFVYSEPKYVDRLRLAMESLKLAMKWDEDRFGLEY
DLEIFNIVAVESFNFGAMENKSLNIFNCSCLLASENITPDYFFTNILSIVGHEYFHNYTGNRVTCRDWFQL
TLKEGLTVYRDQEFSRDCIDRLSEQLGDIEVLRNYQFQEDSGPLAHPIRPDSIVSTNNLYTSTVYRKGAEV
VRMYETILGREGFRKGMDLYFARHDGQAVTCDDFRKAMEDANNYNFTQFERWYDQAGTPEVEVVSIDHNKA
EGTCSITLRQRCSPTPEQPKKRPFYIPVIVGLIGKDSCKEIRQSETLILKEQQQTFILDGVWETPVASILR
GFSAPVNLRFKTPRSDEELAFLFAFDTDEYNRFDAAQTLYKKILIQASTNSSQEISPSSVIESILSQTILS
CFESFISKIKTSKNPNGTVSPMVASYTLRIPSYSQVLASIAEPNFDFVVESFNALTVAFASRFKTQIVDLF
QVLTEDLSALPSQSPFIHGKGVNVEAIAIRRLLNILLDFIAKLDPSLGTKLAFEQHNRFDYMTSKLGAISA
LQIADHKSSECIQALDSLLLATSDDVSTLNQYFSIQASCLIPDNVERVINIYHSNPQFIKYRENPTIFSSL
VGTFASNFVAFNRKDGLGYSFVADAIILMDKVNPMSAASVSRAFTKVLKLDEGRRNLLKENVVRILQSPGL
SKDTSEILKSIDF

>276_106 glutamine synthetase
MNNFKVSSFVCRSFDELYSAIQTENIEYLNYSFCDTFGSLHHITISSNSFKSGKDIQKGIAFDSSSVRGMQ
FGDFSDMIVVPDFEKVWIDPFFDRKTLHVACFVLFHTGEPSPGCVRSISKRAQEALSLSGIADKCFIGPEI
EFFVFESVSYNNSPNHSYFCLDGDEAYWNSGNNATFYPHNRPNLASRRPLKQAYCAPYPVDRDLSLRSEIL
EELENIGVTVEKHHHEVATCQHEIGVHCSTLVQSADIVESIRYLIKGIAHRNNKTATFMPKPLGNDNGSGM
HINISLWKNEKNIFFDPESSYYNLSNQALYFIGGILSHAKAIMAFTNPTTNSYKRLATGYETPTKLSYGAG
DRSSSIRIPLSGFSCTKTQRIEFRIPDSSACPHLAFSAILCAGIDGILHKIHPAAYLNTIKSQSNEYNLPK
SLTEALQNLEEDYEFLIKDGVFSHEFILNYIKLKRDEVVLVESFPTPKEFELYYD

>276_511 Pteridine transporter (BT-1)
MDDKKQTEDLTAKNQLFSLKEFLKKPIIWGPAIYIFTYTAGPDYDDAMFFYFTNRLGFSPTFMGSLRLTYG
IAGIIGIVLYRIILKKTPFREILLWTTLFSIPIYILPLALVTGLNLNMGISNRMFALSGGFLIEAIAEIQL
LPLLVMTAKFCPKGLEGSVYAVMMSIRSLGIGVSKVISAGLAYSLGITAFNFSNLGLLIWISSAFLLLPLF
FLNLVSYHTFSIFQVHFFRL

>Cpnew84 aspartate ammonia ligase

MEVKKQSQFKTVLADIEFIKITFQQLLKESLDLIRISAPLFLEKESGLNDDLSGYEEKVSFTFNNKVLEIV
QSLAKWKRYALKKYELNGLYADMNAIRKSEELDNLHSIYVDQWDWEMVIDTNKGKTKDEILVDIARIIHNN
IYNLERLYWNMKKEKQDIVNIPNENEMIKKELYIISSEELLNMYPNLSSNDREREICKKYGSVFIKQIGKK
LSNNTVHDLRAPDYDDWEYNGDLIYWSNILNGPIELSSMGVRVNKESLIKQLEICNSTERLKLPYCKMLLN
NELPETIGGGIGQSRLCMLILRKEHISQVQCSYWDEKXDXXRYFIIFLKKIL

>264_49, asparaginyl-tRNA synthetase
MIKPEKKFVTEYSVTGRLRIASILDGEDGGVSYIGKKVTVGGWARTVRKQCSDTLLFISLNDGSTSSNLQC
VVEKTVKGFEEGLKATAGCSFKITGTIVKSPAQGQSVELLLNTGDDELKICGLCDASKYPLAKKHHSKEFL
REVAHLRPRSQFFSSVMRIRNSLAIAIHEYFQKNGFMYIHTPIITAADCEGAGEMFQVTTVLPPESKNNIS
NIPASKIEGSQDMAVDYKKDFFGKASYLTVSGQLALENFACSMSDVYTFGPTFRAENSHTTRHLAEFWMIE
PEMAFADLSDNMKLGEGLLKYTVEYVLINNMPDLLYLDKNIENGLVERLKVICKEEFARISYTEAIEMLKP
HDKEFTVPVSWGMDLGSEHEKYITDVLEKRPCIIYNYPKDIKSFYMKLNEDGNTVAAMDILVPKIGEVIGG
SQREDDIEKLENAIKSRNMDPAPYWWYNELRKYGSVPHSGFGLGFERLIMMVTGVENVRDVIPFPRYPNHC
EF

>gi|18996773|gb|AAL83208.1|AF426177_1 inosine-5-monophosphate
dehydrogenase [Cryptosporidium parvum]
MGTKNIGKGLTFEDILLVPNYSEVLPREVSLETKLTKNVSLKIPLISSAMDTVTEHLMAVGMARLGGIGI
IHKNMDMESQVNEVLKVKNWISNLEKNESTPDQNLDKESTDGKDTKSNNNIDAYSNENLDNKGRLRVGAA
IGVNEIERAKLLVEAGVDVIVLDSAHGHSLNIIRTLKEIKSKMNIDVIVGNVVTEEATKELIENGADGIK
VGIGPGSICTTRIVAGVGVPQITAIEKCSSVASKFGIPIIADGGIRYSGDIGKALAVGASSVMIGSILAG
TEESPGEKELIGDTVYKYYRGMGSVGAMKSGSGDRYFQEKRPENKMVPEGIEGRVKYKGEMEGVVYQLVG
GLRSCMGYLGSASIEELWKKSSYVEITTSGLRESHVHDVEIVKEVMNYSK

>gi|44804693|gb|AAS47699.1| thymidine kinase [Cryptosporidium parvum]
MAKLYFYYSAMNAGKSTVLLQSSFNYQERGMKTILFTISKDCRFEEGSICSRIGLSEKAHTFTPDLKLLD
IINQENNKEKIDCVLVDESQFLTKFQVRELCIVVDKLNIPVLCYGLRTDFKGNLFEGSKYLLAWADKLTE
IKTICRCGKKATMTIRLNSNGEPVFSGEQILIGDNSIYTSVCRKHHIINCEEYNF

>gi|10444017|gb|AAG17668.1|AF274310_1 lactate dehydrogenase
[Cryptosporidium parvum]
MIERRKIAVIGSGQIGGNIAYIVGKDNLADVVLFDIAEGIPQGKALDITHSMVMFGSTSKVIGTNDYADI
SGSDVVIITASIPGRPKDDRSELLFGNARILDSVAEGVKKYCPNAFVICITNPLDVMVSHFQKVSGLPHN
KVCGMAGVLDSSRFRTFIAQHFGVNASDVSANVIGGHGDGMVPVTSSVSVGGVPLSSFIKQGLITQEQID
EIVCHTRIAWKEVADNLKTGTAYFAPAAAAVKMAEAYLKDRKAVVPCSAFCSNHYGVKGIYMGVPTIIGK
NGVEDILELDLTPLKQKLLGESINEVNTISKVLDNAPAAGA

>gi|32765705|gb|AAP87358.1| malate dehydrogenase [Cryptosporidium
parvum]
MRKKISIIGAGQIGSTIALLLGQKDLGDVYMFDIIEGVPQGKALDLNHCMALIGSPAKIFGENNYEYLQN
SDVVIITAGVPRKPNMTRSDLLTVNAKIVGSVAENVGKYCPNAFVICITNPLDAMVYYFKEKSGIPANKV
CGMSGVLDSARFRCNLSRALGVKPSDVSAIVVGGHGDEMIPLTSSVTIGGILLSDFVEQGKITHSQINEI
IKKTAFGGGEIVELLKTGSAFYAPAASAVAMAQAYLKDSKSVLVCSTYLTGQYNVNNLFVGVPVVIGKNG
IEDVVIVNLSDDEKSLFSKSVESIQNLVQDLKSLNL

>hypothetical protein B (276_500), CAD98502.1
MIFQYETQSFVSLVLLCDRIFLMHHARRSSLNADHTAIMVIFPSILMNLIIIDKKDINEDLTVELSSRQS
NHCISVLEINIGSKVNVGVKNSGKGTATVTKIMRKETNKDGITKSESIINLSSIEEIGNKVNNRSRRSIS
EINNLNNFQYAVTIKLDTEIHKEEYKHDYPLIDLLIALPRPKVFEKVLQNAVTIGVGRIIFVCTDKSEKS
YLNSSKLKKESIDEIVQLGLEQASKTLCPDVYVYASWTFFLQHLRNYFFCKESMIGIVADVLGNSKITEI
GLQSHTGPIILAIGPEGGWTKEELTDLITMGFKVVNMGDRILKVETALISLYSKVSFGRTQRN

>gi|44804700|gb|AAS47700.1| uridine kinase uracil
phosphoribosyltransferase [Cryptosporidium parvum]
MSNISLEKLISKDIYYGDQALTPSSNSNVFVIAVAGGSASGKTSVCTRIFSELGDKRVTVIETDSFYKTP
VLEEGQTMADYNFDHPNSVDFELLYNVLLSLKNGEGVHIPNYCFKQHKRLETGRKVSPASIIIVEGIFIL
FHPKIRHLINMSIFVDTDDDIRLVRRIRRDTIERGRQIDDILNQYEKTVKPSYDEFIYPTRRYADIVIPH

YPNEVAVDLVVQHLRYKLKMDDLRKIYSNLHIIPSNCQIRHMHSIIRNKDTSVVDFVFWSDRLIRLVVEN
ALSHLSFTGQTIETPIGELYDGVQFNYKDKLCAVSIVRGGESMEIGLSAVCKDIPIGKILLEFQNPKTEL
DAQFDKPKIIYCKLPDDIASRNVFILDPILGNGFGVFSAIKYLLSKGVLQRKIIVLSLIVAHNAIHRICK
EFPEVTLITTEIDRDVNSDGFVIPGLGCFADRYFGTE

>gi|44804760|gb|AAS47705.1| calcium-dependent protein kinase 1
[Cryptosporidium parvum]
MLNIEQNADECAKRVGANMVPVLHNVTEECNIKAEEKEIVPEVEEAKSESNNCLDSDDYLEGHIYAAMCT
KCAGELQQKLNPTEPYRDPKKVSGKEQISRGLIIESKSFVDANKNIKFSKRSDKNEYAGLCSSPEVTTPN
GERETSTDSNIKNTESTKVSHGIFDRTCLIQEHALVNRNINDFYELNLGNLGRGSYGSVVKAIDKQSGAQ
RAVKIILKPKLENINRLKREILIMKRLDHPNIIKLFEVFEDTNYLYFVMEICTGGELFDRIIKRGHFSER
YAAVIMRQVFSAIAYCHSNEFMHRDLKPENLLFSDSSPNSLLKVIDWGFAAKCPKTHKFTSVVGTPYYVA
PEVLYGSYSKLCDLWSAGVILYILLCGYPPFHGKDNVEILRKVKIGQYSLEHNSWKYVSDSAKDLIKRLL
MTDPNKRISAQDALNHPWIKSQISSPNTADATYFTNDVCNSLLARFRDFQRQSKLKKLALTCVAYHLNDA
DIGALQKLFSTLDRNGDGVLTINEIRSALHKIQNVSQLGDDIDNLLMELDTDGNGRIDYTEFIAASIDHK
LYEQESLCKAAFKVFDLDMDGRISPQELSRVLNITFLQEAFEQSTIDSLLKEVDINQDGYIDFNEFMKMM
MGDKHEQKLETKVQKIEDEGSGNKKLSKGGIISDIFGATSIFKKLNN

>gi|44804774|gb|AAS47706.1| calcium-dependent protein kinase 2
[Cryptosporidium parvum]
MMGQGQNALNGVCSRRYEKDSIEINKNKEVRRKQSNKRGNFPAHNMTHGKPEYLKEAVDRLINHGNIVGN
SPDRYLAKERKSQASCSTSDPTSPLAYRNGNDNYFEQYEICDLIELTSEMALSNESRFESLSEYESIPKL
SPSTTVMGITDVSATNTSNSNSATPTESEKGSGPKLSLTEGKFRREGLIPACKGSIHSDYIIDSGRIGKG
TYGSVKSGTNRLTGCIRAIKTIPLTRVEALDNFMKEINILKNLDHPNIVKLYETYQDKENIYLVMELCSG
GELFDRIISQGSFDEIYAANLMKQVLSTICYCHDHGIVHRDLKPENFLFLNKNYNAPLKIIDFGLAARVN
NEDTSLNTRAGTPYYVAPEVLQGKYDKQCDMWSLGVILYILLCGYPPFHGSNDSIILHKVQKGVYAFKEE
DWKHVSFLAIDLIRKLLTYNPSERITARDALNHPWITRFADDILFLSPRNYFYSNDGFMKTGIVSSDYID
SSINNLKALECQQKPRNCNLMGSRRNGYIKGGLRYCRSKRRSISSSSGLNLLSNFRAFHKYNRFMKVALT
VIAQQMTESQISNLKEAFILLDANCDGTLTPQEIITGLKNSGITELPSDLLAILNDIDSDGSGSIDYTEF
IAATLDSKQYSKEQVCWAAFKVFDQDGNGKITANELLNVFSYNSEQGSAGINDKALSDVKNMIKEVDVDG
DGEIDFQEFLEMFRRSNS

>gi|44804783|gb|AAS47707.1| calcium-dependent protein kinase 3
[Cryptosporidium parvum]
MTVATSSRRNSRRSSLSSESSLTSHTSSTKPKNSYIQKNSSVSGGNGTSHNCYSHKGRSGSGFGSSSSYE
NRAYLVSGGGSCIGNNTSMGNANGSLLAPNVQLDNLPSERVGNGSPNWALSADVCSLKDFKELIECKNEH
AESVLRKYSGIKSVNGGRGIRQVLASIVRGSRTSKNRSSSVDSGSCGMVSSSTSTTTSSAALVSNLNRKN
TTGGDINNTRIKIEDSIDGNGTIINDNNGSSTSNRECNILTGQPIQKAILTQRDIMELRGKIDKVLSTED
SFVRSALQSFQQFDFDGDGNLTIEELLDLLTTLGEHLALPPINRKVVANEISIRLNSKLTVDSSASESKM
LISFPFFLKYYLSVLTTIRQKHFSSVKINGELQQNKAIRKHLVHEDDINDLYTFHYQLGTGTYGEVFLVT
ENYTRQRRVCKIVDKVKCRRKLDEIDHEVEILKQLDHPGLVHIYEVYEDRLNLYFILEFCAGGNLFHSLQ
DAIQSGFRFSEFHVSRIIQQILLAIRYLHLKRVVHKNLKPQNILLTNSFGSGNTSVKLVDFGLAEIFSSD
EINLPELTTSNVSPSHFHSPLKSLPLSSSSSSSYITQDHIKTHNQAQPQAQIQGHTHNTSLSSISNQNRA
SEEGYLFASVDEVTGIHKSNSSHNSSPSSNSSLAQNLHQEESLASYPKYLDFTAPELLKGETFSYSLDVW
SVGVIMYSVITGRHPFSGRSRAETRHNICFGIPAISEITCVSNACKSLLGKLLEKNPRERITVDEALSHD
WFSISHSSYCEVDIGMPLLAHLKVYTRQSDLRHILIHMLSHQLALDTTQINMATSVFKSLDTDNDGVLSI
SELGSGLQKLGISSRESSMIIKAMDIDGNGIISYSEFITACHIWRRNEIRQLKSFFMKIDRNNQGKITRE
EFKQLLKAQKSKLLSNITKSFSSTNDVNYRTILPSSNQGVYTEWDSVLDEIDTNRDGLIDWKEFCTFIVD
YFNTSRESLRACYNNSSISNISSTFSIDPPSVAGLSRRRDSGICPVQEANQHPPPHPSQLD

3) **Genbank or other acession numbers for sequences used in Figure 4.**

Leucine aminopeptidase

1. 23509661 Plasmodium
2. TA09045 Theileria (Sanger GeneDB http://www.genedb.org)
3. 46227265 Cryptosporidium
4. 15235763 Arabidopsis
5. 1703282 Solanum
6. 23042880 Trichodesmium
7. 17227733 Nostoc
8. 15607055 Aquifex
9. 24216140 Leptospira
10. 15611584 Helicobacter
11. 27364860 Vibrio
12. 17547134 Ralstonia
13. 13476527 Mesorhizobium
14. 27887802 Fusobacterium
15. 29840173 Chlamydophila
16. 16080258 Bacillus
17. 19074788 Encephalitozoon
18. 19909972 Coprinopsis
19. AY581147 Dictyostelium
20. 19113905 Schizosaccharomyces
21. 21355725 Drosophila
22. 7705688 Homo
23. 29832567 Streptomyces
24. 21674006 Chlorobium
25. 5870602 Leishmania

G6PI

1. 15595075 Borrelia
2. 16752000 Chlamydophila
3. 46227265 Cryptosporidium
4. 4835820 Toxoplasma
5. 23509563 Plasmodium
6. 15239045 Arabidopsis
7. 1169797 Oryza
8. 37960111 Phytophthora
9. 15804618 Escherichia
10. 23136626 Cytophaga
11. 17510665 Caenorhabditis
12. 17737445 Drosophila
13. 18201905 Homo
14. AY581147 Entamoeba

15. AY581146 Dictyostelium
16. 6319673 Saccharomyces
17. 7437359 Trypanosoma'
18. 21673816 Chlorobium
19. 15964236 Sinorhizobium
20. 15806745 Deinococcus'
21. 29832844 Streptomyces
22. 19173604 Encephalitozoon
23. 23125809 Nostoc
24. 18030019 Spironucleus
25. 13924524 Giardia
26. 18030022 Trichomonas
27. 15644137 Thermotoga
28. 16080187 Bacillus
29. 15669801 Methanococcus

Tryptophan synthetase beta subunit
1. 34557127 Wolinella
2. 46226438 Cryptosporidium
3. 22958844 Rhodobacter
4. 28900440 Vibrio
5. 33456993 Cycloclasticus
6. 16079321 Bacillus
7. 14520675 Pyrococcus
8. 11499193 Archaeoglobus
9. 15606106 Aquifex
10. 32417954 Neurospora
11. 24215988 Leptospira
12. 15642912 Thermotoga
13. 29345943 Bacteroides
14. 34557707 Wolinella
15. 15963781 Sinorhizobium
16. 17546702 Ralstonia
17. 1174780 Zea
18. 17231286 Nostoc
19. 33239640 Prochlorococcus
20. 15805965 Deinococcus
21. 29832719 Streptomyces
22. 28898734 Vibrio
23. 15612264 Helicobacter
24. 19703662 Fusobacterium
25. 29840324 Chlamydophila
26. 14520795 Pyrococcus
27. 18313363 Pyrobaculum
28. 15606594 Aquifex
29. 11498839 Archaeoglobus
30. 15643305 Thermotoga
31. 29350072 Bacteroides

4) GI accession numbers for the sequences used in Figure 5, 1,4-alpha-glucan branching enzyme sequences are listed in the order in which they appear on the tree.

45548447 Rubrobacter
29829347 Streptomyces
45916434 Mesorhizobium
22986676 Burkholderia
23468472 Pseudomonas
22968855 Rhodospirillum
15806848 Deinococcus
15618386 Chlamydophila
22971471 Chloroflexus
15606119 Aquifex
22999765 Magnetococcus
32398905 Cryptosporidium
995313 T_gondii
32472075 Pirellula
23021493 Clostridium
17228208 Nostoc
45509261 Anabaena
23126496 Nostoc
23474508 Desulfovibrio
18309045 Clostridium
19704191 Fusobacterium
16080150 Bacillus
32470978 Pirellula
45916029 Mesorhizobium
45548449 Rubrobacter
22968918 Rhodospirillum
22986678 Burkholderia
23471845 Pseudomonas
23475793 Desulfovibrio
22968087 Rhodospirillum
22971470 Chloroflexus
23124761 Nostoc
46135186 Anabaena
23130645 Nostoc
32398951 Cryptosporidium
XXXXXXXT_gondii
23050680 Methanosarcina
23123689 Nostoc
23135626 Cytophaga
29346181 Bacteroides
28829250 Dictyostelium
171569 Saccharomyces
16416077 Neurospora

4557619 Homo
17554896 Caenorhabditis
32564391 Caenorhabditis
28573410 Drosophila
30680140 Arabidopsis
15227650 Arabidopsis
2801805 Gracilaria
1169912 Solanum
29245217 Giardia

5) **Expression of a *C. parvum* GFP reporter construct containing the N-terminal extention region of phosphoglucomutate in *T. gondii***

The *C. parvum* phosphoglucomutase N-terminal extension targets a GFP reporter to a dense granule secretory pathway in *T. gondii*. The secreted protein is retained in the vacuolar space surrounding the parasites. A) Fluorescent image illustrating the location of the tagged protein in a single vacuole containing 4 *T. gondii* parasites. B) Phase contrast image of the same vacuole.

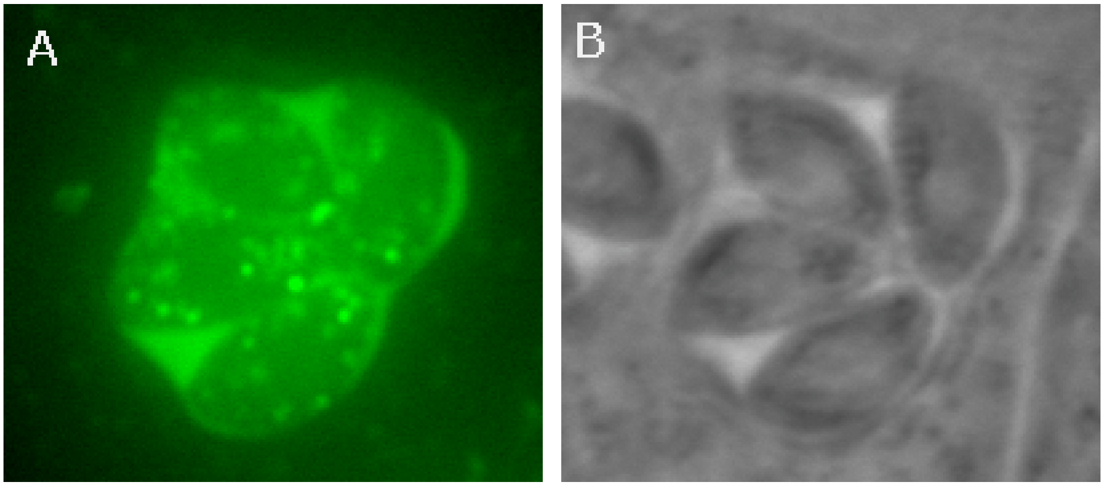

6) **Primers used for semi-quantitative RT-PCR experiments**

Gene Name	Forward Primer	Reverse Primer
glucose-6-phosphate isomerase	5′GCACTAATGCTCAACACAGTTTTTAT	5′GTTGGCCAATATAATATGCACTACAG
leucine aminopeptidase	5′ATTATCACTGCCTCTAATGGAAAAAC	5′ATTTCACATTCTTGTCTACAAACTCG
biopterin transporter (BT-1) family protein	5′TTGCAGGTATAATTGGAATTGTTCTA	5′CAAGACTTCGTATTGACATCACAACC
calcium dependent protein kinase	5′GAAATTAGCTCTTACATGTGTTGCAT	5′ACTTCCTTTAACAGCGAGTCTATTGT
dihydrofolate reductase-thymidine synthetase	5′AACCTTTATCAAAGGAGTTGTGACTT	5′ATCTCCATTTTAATAGTGGGATATGG
tyrosyl-tRNA synthetase	5′TCTGCAATATTTGTTGAAGATACTCC	5′GTCAGTATTAAAATGGTCACGAATTG

7) Phylogentice tree of Aldolase

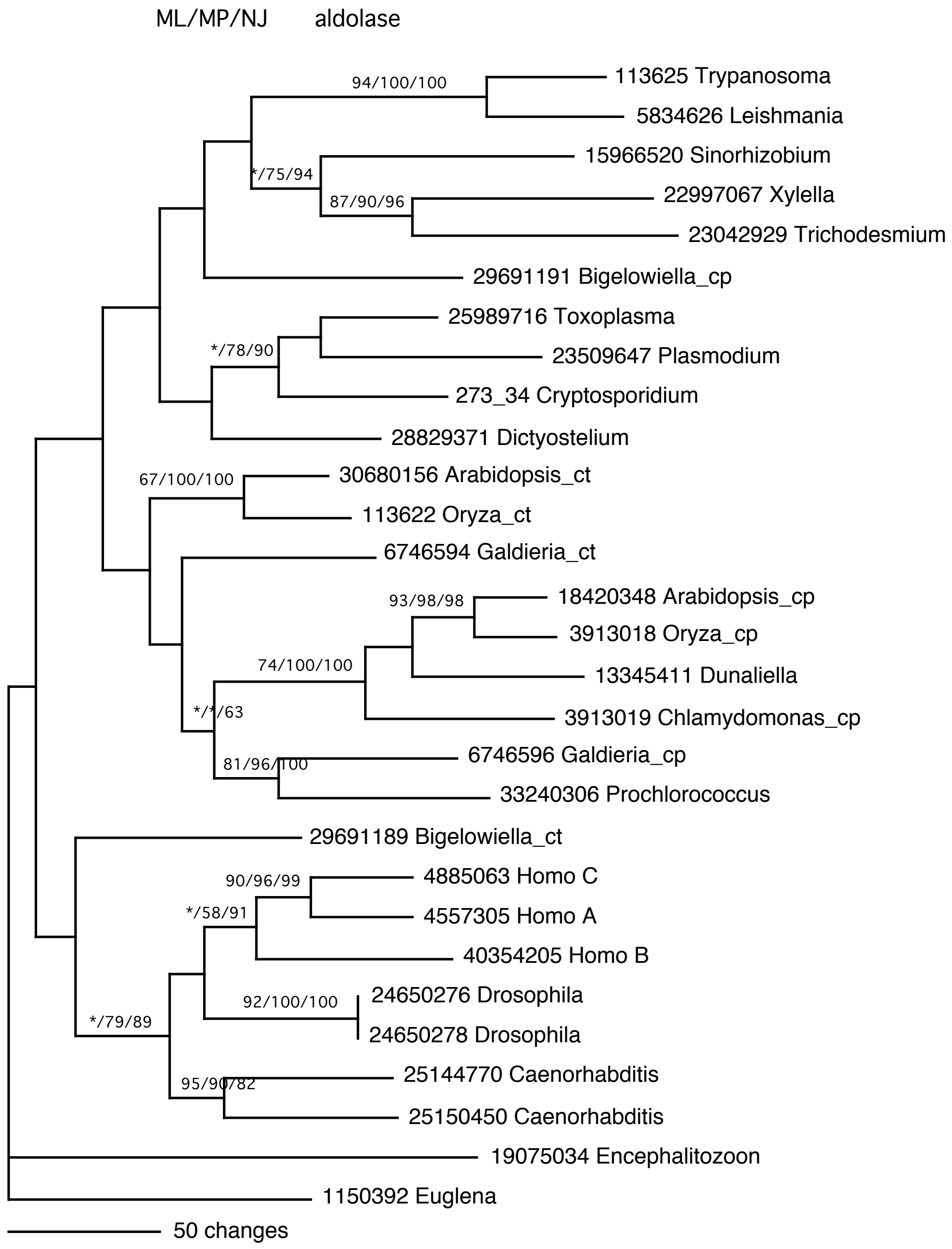

8) **Multiple sequence alignments used to create phylogenetic trees in Figure 4**

Leucine aminopeptidase (Fig. 4a)

```
CLUSTAL X (1.81) multiple sequence alignment


_23509661_Plasmodium_falcip         ASQLIAAPSNYCNPVSLSNAAVELAQKLNLEYKILGVKELEELKMGAYLS
Theileria_annulata_sanger           AKELTTAPPNYANTVTVSEFLKKMLSSVGLKVKVLDYDDCKKLKMFCYLS
268_40_Cryptosporidium_par          ARDLTSAPPNYCDPVNMAKEVISMAKSVGLEGKILQPKECEELKMGAFLA
_15235763_Arabidopsis_thalia        GKELVNSPANVLTPAVLAEEALNLASMYSMTANILNEEQCKELKMGSYLA
1703282_Solanum_cp                  GRELVNAPANIVTPGALAEEAKKIASTYSITVNILDAEQCKELKMGAYLG
23042880_Trichodesmium_erythr       ARELVAAPANSCTPITMAETAQTLAKEFGLTLEILEKEDCEKLGMGAFLG
17227733_Nostoc_sp                  ARQLVAAPANSVTPITMAETAQQIAQDYGLQIEILEQEDCEKLGMGAFLG
15607055_Aquifex_aeoli              ARNLVNEPGNVINPITLAEEAKKLAEEFGLECKVYDEKQIQEMGMMALYS
24216140_Leptospira_interro         VRHIAHLPANHFTPEEFVSRSKEIAKDNGLKITVFDEPQLKKEKMGGILS
15611584_Helicobacter_pylori        VRDLVNTPPMIGTPVYMAEVAQKVAKENHLEIHVHDEKFLEEKKMNAFLA
27364860_[Vibrio_vulni]             CKDLGNMPPNIANPAYLASQARRLADDYEITTKIIGEQEMEKLGMSSYLA
17547134_Ralstonia_solan            TRDLGNLPSNICTPTYLANTARQIAKDFKLKVEVLGRKQIEALKMGAFLA
13476527_Mesorhizobium_loti         ARDLVNEPANILGPVEFAARAKELE-TLGVKVEILTEKEMKKLGMGSLLG
27887802_Fusobacterium_nuclea       VKDLVNEQAEVLNPKELADRATKLGKEFGFNVEILDEKKAQKLGMNAYLA
29840173_Chlamydophila_caviae       TRDLVNGNADEVTPQKLANIAKGMAKEFPMDVKILNKDAILKEKMGLLAA
16080258_Bacillus_subt              ARTLVNMPGNMLTATDLASYAEELAAKYDFECEILEKSEMEELGMGGILA
19074788_Encephalitozoon_cuni       ARFLGDTPANLMNPTLFVEYATKYLQDKKVTFEVFDKSFMERKSMNLLLG
19909972_Coprinopsis_cinere         ARTLMEYPANMMTPTLFTERVKQEFAGIPVEIIVRDEAWAAEKGMNVFLS
Dictyostelium_discoid               ARVLMETPANLLTPTNFVQHVSSQMKELIVEMIVREEQWVKDQKMGMFWG
19113905_Schizosaccharomyces_p      ARSLMECPANYMTSLQFCHFAQELFQNSSVKVFVHDEKWIDEQKMNGLLT
_21355725_Drosophila_melano         TRQLQEMPSNLLTPTAFAQNVVEVLCKSGVNVEVKVEGWAESQSMHAFLA
_7705688_Homo_sapien                ARQLMETPANEMTPTRFAEIIEKNLKSASTEVHIRPKSWIEEQAMGSFLS
29832567_Streptomyces_averm         ARDLINTPPNDLNPESFAAVAQAAAKEHGIKVQVLDEKALTKGGYGGILG
21674006_Chlorobium_tepid           ARDLVNLPGNHLSAEDLAEAAIEAGKRGGEVT-VFDKKKIVELGMGGLLA
_5870602_Leishmania_major]          ARNLGNLREDEGVPEFYAEWAKKYMIPEGKVRKVLRGQQLEEAGLHLMYN
                                       :         .                   :

_23509661_Plasmodium_falcip         VGKGSMYPNKFIHLTYKSGDVKKKIALVGKGITFDSGGYNLKAMIDLMKF
Theileria_annulata_sanger           VCQGSKFPPKFLHAVYKPGEVKKTLAFVGKGITFDAGGYNVKNMIHYMKL
268_40_Cryptosporidium_par          VAQGSKSPAQFVHLTYKPGEIKKRIALVGKGITMDTGGYNIKHMIHFMKG
_15235763_Arabidopsis_thalia        VAAASANPPHFIHLIYKPGPVKTKLALVGKGLTFDSGGYNIKTLIELMKF
1703282_Solanum_cp                  VAAAAENPPYFIHLCFKTRERKTKIALVGKGLTFDSGGYNLKTKIELMKN
23042880_Trichodesmium_erythr       VAQGSDLPPKFIHVTYKPTPRR-KLAIVGKGLTFDSGGLNLKVGIEMMKI
17227733_Nostoc_sp                  VALASDLPPKFIHLTYKPTPKR-KLAIVGKGLTFDSGGLNIKGGIETMKI
15607055_Aquifex_aeoli              VGKGSATPPRFIHLIYKPGKPKEKIALVGKGLTFDSGGLNIKPYMRTMKM
24216140_Leptospira_interro         VCEGSDKKAKMILLEYTPPITKKKLAIIGKGLTFDSGGISIKPDMHEMKY
15611584_Helicobacter_pylori        VNKASLAVNPPRLIHLVYKKAKKKIALVGKGLTYDCGGLSLKPYMVTMKA
27364860_[Vibrio_vulni]             VGRGSKNESMMSIIEYKGDSEAKPIVLVGKGLTFDSGGISLKPGMDEMKY
17547134_Ralstonia_solan            VTKGSQEPPQFIVLRYEGPAKQAPVVLVGKGITFDTGGISLKPGMDEMKF
13476527_Mesorhizobium_loti         VAQGSPRGARMAVMQWNGKPKDGPIAFVGKGVTFDTGGNSMKPGMEDMKG
27887802_Fusobacterium_nuclea       VARAAYHRPYVIIMRYKGKSKYT-YGLVGKGLTYDTGGLSLKPSMLTMRC
29840173_Chlamydophila_caviae       VAKGSAVDPCFIVLTYQGKSKDH-TVLIGKGVTFDSGGLDLKPAMLTMKE
16080258_Bacillus_subt              VNQGSTEPPKMIVLKYQGKEWEDVVGLVGKGITFDTGGYSIKTGIVGMKS
19074788_Encephalitozoon_cuni       VSQGSAQEPKLLVARYRGDAVD--IALVGKGVCFDSGGISLKPRMHRMKG
19909972_Coprinopsis_cinere         VTRGTSEPAKFLEIHYKGDKNAQPLAFVGKGITFDTGGISLKPGMKLMRG
Dictyostelium_discoid               VAKGSDEPLKFLELHYRGADGKDSIVYVGKGITFDSGGISIKPNMGLMKG
19113905_Schizosaccharomyces_p      VNAGSDIPPRFLEVQYIGSKDDGWLGLVGKGVTFDSGGISIKPNMKEMRA
_21355725_Drosophila_melano         VGKASCEPPIFLELSYYGAEER-PIVLVGQGITYDAGGLCLKKELFNMRG
_7705688_Homo_sapien                VAKGSDEPPVFLEIHYKGANEP-PLVFVGKGITFDSGGISIKANMDLMRA
29832567_Streptomyces_averm         VGAGSAAGPRLVKLSYTSSKAKKHLAFVGKGITYDSGGISLKPHNETMKC
21674006_Chlorobium_tepid           VNKGSEQPPTFVILDYKPGKAKKTIALVGKGVTFDSGGISLKPGMDEMKS
_5870602_Leishmania_major]          VGRGSRYEPYLVVLEYIGPRSSATTAIVGKGVTFDCGGLNIKPSMETMHS
                                    *  .:                      :*:*:  * **  :*     *:

_23509661_Plasmodium_falcip         DMSGCAAVLGCAYCVGTLKPNVEIHFLSAVCENMVSKNSYRPGDIITASN
Theileria_annulata_sanger           DMGGFATVFSAAYALGKLKPNVELHFVGGLCENMLDANSYRPSDVVTASN
268_40_Cryptosporidium_par          DMGGAAAVFGTALSVGLIKPNIEVHFISAICDNLVSRDAYLPGCIITASN
_15235763_Arabidopsis_thalia        DMGGSAAVLGAAKAIGQIKPGVEVHFIVAACENMISGTGMRPGDVLTASN
1703282_Solanum_cp                  DMGGAAAVLGAAKALGEIKPGVEVHFIVAACENMISGAGMRPGDIVTASN
23042880_Trichodesmium_erythr       DMGGAGTTFGTIKAIAQLKPDVEVHFISAVTENMVSGHAIHPGDFLTASN
17227733_Nostoc_sp                  DMGGAAATLGAAKAIAQIKPNVEVHFISAVTENMISGKAMHPGDILTASN
15607055_Aquifex_aeoli              DKSGACAVLGIMRAIAQLKPDVEVHGLIGAAENMPDGNAYRPDDVIKAKN
```

```
24216140_Leptospira_interro     DMCGAATAIHAIGAIAELGLGVPVIAAIGVAENMPDAAAIKPGDVYTAYN
15611584_Helicobacter_pylori    DKGGGSAVIGLLNALAKLGVEAEVHGIIGATENMIGPAAYKPDDILISKE
27364860_[Vibrio_vulni]         DMCGAASVFGTMKALAKLNLPVNVIGVLAGCENMPGSNAYRPGDILTTMS
17547134_Ralstonia_solan        DMCGAASVLGTLRAVAEMGLKLNVIAVVPTCENMPSGIATKPGDVVTSMS
13476527_Mesorhizobium_loti     DMGGAAAVTGLMHALAARKAKANVVGIIGLVENAVDGHAQRPGDIVTSMS
27887802_Fusobacterium_nuclea   DMGGAATMIGTMCAVAKMKIKKNVTCVVAACENSIGPNAYRPGDILTAMN
29840173_Chlamydophila_caviae   DMAGAATVLGILSGIAALELPVNVTAIVPATENAIDAAAYKMGDVYTGMS
16080258_Bacillus_subt          DMGGAAAVLGAMETIGELRPEQNVLCVIPSTDNMISGGAMKPDDVIVSLS
19074788_Encephalitozoon_cuni   DMLGAASVLSVFGLAADMGIKINMNLVIPLVENLPSGTATKPGDVHVGMN
19909972_Coprinopsis_cinere     DMGGAATVVSAALAIAKLQLPINLVVTTPLTENMPGPSATKPGDIIYAMN
Dictyostelium_discoid           DMGGAATAVSAMFGVASLGLVNLITITPLCENMPSGKATKPGDILTAANG
19113905_Schizosaccharomyces_p  DMGGAAVMLSSIYALEQLSIPVNAVFVTPLTENLPSGSAAKPGDVIFMRN
_21355725_Drosophila_melano     DMTGAAVVVATCRAVAGLRLPVNIRGLIPLCENVVGCNSFRPGDMVKSMN
_7705688_Homo_sapien            DMGGAATICSAIVSAAKLNLPINIIGLAPLCENMPSGKANKPGDVVRAKN
29832567_Streptomyces_averm     DMSGAAAVFAAVVAAARLGLEVNVTGWLALAENMPSGTATRPGDVLRMYS
21674006_Chlorobium_tepid       DMSGAAVVIAAIEAAASLGLPLRVVGLVPATDNMPGGSAQKPGDVITTMS
_5870602_Leishmania_major]      DMMGAATAFCTLKAIAKLQLPINVVAAVGLVENAIGPESYHPSSIITSLK
                                *  *                           :   .      . .

_23509661_Plasmodium_falcip     GKTIEVGNTDAEGRLTLADALVYAEKLGVDYIVDIATLTGAMLYSLGTSY
Theileria_annulata_sanger       GKTVEVLNTDAEGRVTLADSLYYAAKLDPDYLVDYATLTGAAMTALGVKC
268_40_Cryptosporidium_par      GKTIEVGNTDAEGRLTLADALVYACNLKVETVIDLATLTGANYRLFEGRY
_15235763_Arabidopsis_thalia    GKTIEVNNTDAEGRLTLADALVYACNQGVDKVVDLATLTGACIIALGTSM
1703282_Solanum_cp              GKTIEVNNTDAEGRLTLADALIYACNQGVEKIIDLATLTGAIVTALGPSV
23042880_Trichodesmium_erythr   GKIIEVNNTDAEGRLTLADALVFAEKLGVDAIIDLATLTGACVVALGNDI
17227733_Nostoc_sp              GKTIEVNNTDAEGRLTLADALVYTDKLGLDAIVDLATLTGANVIALGDDI
15607055_Aquifex_aeoli          GKYIEIDNTDAEGRVTLADVLSYASEL-KDKIIDMATLTGACMVALGEYT
24216140_Leptospira_interro     GITVEVQNTDAEGRLVLGDVLSYVGKKFKDYMLDLATLTGAIIISLGHEA
15611584_Helicobacter_pylori    GKSIEVRNTDAEGRLVLADCLSYA-QDLSDVIVDFATLTGACVVGLGEFT
27364860_[Vibrio_vulni]         GQTVEVLNTDAEGRLVLCDALTYV-ERFEDCVVDVATLTGACVIALGHHI
17547134_Ralstonia_solan        GQTIEILNTDAEGRLILCDALTYV-ERFRAAVIDVATLTGAVIIALGHIN
13476527_Mesorhizobium_loti     GQTIEVLNTDAEGRLVLADALWYCNDRFQKFMVNLATLTGAIMVALGLHY
27887802_Fusobacterium_nuclea   GKTIEVTNTDAEGRLTLADALTYIVRKEKNEVIDAATLTGAVMVALGEDV
29840173_Chlamydophila_caviae   GLSVEIGSTDAEGRLILADAITYALKYCNTRIIDFATLTGAMVVSLGEDV
16080258_Bacillus_subt          GKTIEILNTDAEGRLVLADGITYAKQHG-SVLVDVATLTGGVVVALGTET
19074788_Encephalitozoon_cuni   GKSVEINNTDAEGRLILADALVYAQ-EANTYIVDVATLTGAMMIALGDAF
19909972_Coprinopsis_cinere     GKSVEVDNTDAEGRLVLSDAIYYTSTEYKHTLIDVATLTGAMVIALGEVY
Dictyostelium_discoid           KTVEVDNTDAEGRLILGDALHYACS-FKTHIIDIATLTGAIDVALGQHYA
19113905_Schizosaccharomyces_p  GLSVEIDNTDAEGRLILADAVHYVSSQYKKAVIEASTLTGAMLVALGNVF
_21355725_Drosophila_melano     GKTIEVQCTDHEDVLVLADALLYAQN-FCKCIIDIGTCSGYMRQSLDEAA
_7705688_Homo_sapien            GKTIQVDNTDAEGRLILADALCYAHT-FNKVILNAATLTGAMDVALGSGA
29832567_Streptomyces_averm     GKTVEVLNTDAEGRLVLADALWAASE-EKDAIVDVATLTGAMMLALGNRL
21674006_Chlorobium_tepid       GITVEVGNTDAEGRLILADALFYAKKEYNDVIIDLATLTGACIVALGNSV
_5870602_Leishmania_major]      GLTVEVLNTDAEGRLVLADVLTYVQKDKKTRIIDLATLTGAIIVGLGSRR
                                              :                :       .

_23509661_Plasmodium_falcip     AGVFGNNEELINKILNSSKTSNEPVWWLPIINEYRATLNSKYADINNISS
Theileria_annulata_sanger       AAFYSNDEDLAELYTNSARNSGELMWRMPLLKEYKDYLNSKVADYANVYN
268_40_Cryptosporidium_par      ASVLGNNDELFQMIQTCGKTVYEKFWQLPLDPEFQEMVKSEIADLNNTSE
_15235763_Arabidopsis_thalia    AGIYTPSDKLAKEVIAASERSGEKLWRMPMEESYWEMMKSGVADMVNTGG
1703282_Solanum_cp              AGAFTPSDGLAREVVVAAEASGEKLWRMPMEESYWESMKSGVADMINTGP
23042880_Trichodesmium_erythr   AGLWSPNDNLAAEITAAAEKAGEKMWRMPLEEKYFEGLKAMHADMKNTGP
17227733_Nostoc_sp              AGLYTPDDALAGQLEQAASESGEKIWRMPLEEKYFEGLKSGIADMKNTGP
15607055_Aquifex_aeoli          AGLFTNAPDFAEEIKKTAKRTGERVWELPMDDELRKKIKNTVADVLNTGG
24216140_Leptospira_interro     AGVMSNSDVLTNLLKEASISSDERIWEMPLWEEYSEDLKSDIADIRNVAG
15611584_Helicobacter_pylori    SAIMGHNEELKNLFETSGLESGELLAKLPFNRHLKKLIESKIADVCNISS
27364860_[Vibrio_vulni]         TGVLSNHNPLAHELVNASEQSSDRAWRLPMADEYHEQLKSPFADMANIGG
17547134_Ralstonia_solan        TGVYARSDALANALLAAGKQSLDTGWRMPLDEEYQELLKSNFADMGNIGG
13476527_Mesorhizobium_loti     AGLFSNNDELAERLAAAGQATQERLWRMPLGAEYDKLIDSKNADMKNIGG
27887802_Fusobacterium_nuclea   TGVFTNDDKMARKVIDASENWNEYFWQMPMFDIYKKNLKSSYADMQNTGV
29840173_Chlamydophila_caviae   AGFFSNNDVLAQDLSEASAETSEALWRLPLVEKYNKALHSDIADMKNIGS
16080258_Bacillus_subt          TGAMTNDQSFYQQVADAAQECGEAIWQLPITEKDKKRVKSSMADLSNSPG
19074788_Encephalitozoon_cuni   IGYFTADDDLSKIIHQSGIDANDPVWRMPLSQLYLPSMKSNVADLKNAEG
19909972_Coprinopsis_cinere     SGVFASSDELWQQLYEAGQIEHDRMWRMPLDDEFGPQIHSSNADLQNTGG
Dictyostelium_discoid           GCFTTTDSLWDQLNECGNISGERLWRMPLIPEYRKQMETSVADLINSAGR
19113905_Schizosaccharomyces_p  TGAFVQGEELWKNLETASHDAGDLFWRMPFHEAYLKQLTSSNADLCNVSR
_21355725_Drosophila_melano     CGVFTNSEILWQQIKHASMHTGDRVWRFPLWNFYSKAVRAGRSDVQNYGI
_7705688_Homo_sapien            TGVFTNSSWLWNKLFEASIETGDRVWRMPLFEHYTRQVVDCLADVNNIGK
29832567_Streptomyces_averm     FGVMANDDAFRTAVVEAAEEVGEDSWPMPLPEHLRKGMDSPTADIANMGE
21674006_Chlorobium_tepid       AGLFSNDEKLAESIFEAGQSSGEKVWRLPLWDEYDELIKSDVADVHNTGG
_5870602_Leishmania_major]      AGLFSPSAMLANSLMAAGTQCGEELWPMPIGDEHKDMMKGGIADLINAAP
                                                             :                .
```

```
_23509661_Plasmodium_falcip           SVASSIVASLFLKEFVQNTAWAHIDIAGVSWKPKGFGVRLLTEFVLN
Theileria_annulata_sanger             GKCGSINGALFLKEFVDD-KWLHVDFAGPAFKGTGFGVLTIVNFVLT
268_40_Cryptosporidium_par            GKAPISTSAAFLCEFVDKVKYAHIDIAGCSGFGNGFGVKTLVTIIKE
_15235763_Arabidopsis_thalia          RAGGSITAALFLKQFVSEVEWMHIDMAGPVWAATGFGVATLVEWVQN
1703282_Solanum_cp                    RDGGAITGALFLKQFVDEVQWLHLDIAGPVWNATGYGVSTLVEWVLR
23042880_Trichodesmium_erythr         RPGGAITAALFLKQFVKNTPWAHLDIAGPVWGATGYGVRTLVNWILS
17227733_Nostoc_sp                    RPGGSITAALFLKQFVKDTAWAHLDIAGPVWGATGYGVRLLVDWVLS
15607055_Aquifex_aeoli                RYGGAITAAMFLEEFVGEIKWVHLDIAGPAWGGTGFGVRTCLEYIMK
24216140_Leptospira_interro           RAGGSLSAAKFLERFVEPIAWAHIDIAGTAWGPTGYGVRLLVDLVEK
15611584_Helicobacter_pylori          SRGGAITAGLFLNEFIRDDKWLHIDIAGPAYGASGAGVRACTAFVEE
27364860_[Vibrio_vulni]               RPGGTITAACFLSKFAKK-NWAHLDIAGTAWGSTGRPVSMLVQFLLN
17547134_Ralstonia_solan              RPAASVTAACFLARFTEK-DWAHLDIAGTAWGATGRPVPLLTRFLMD
13476527_Mesorhizobium_loti           RYGGAIIAAQFLQRFVKDTPWAHLDIAGTAMWGSGFGVRLLDRLVRD
27887802_Fusobacterium_nuclea         RWGGSTNAAKFLEEFIDDTKWVHLDIAGTAWGATGQVFRTVYSYIKD
29840173_Chlamydophila_caviae         NRAGAITAALFLKRFLEDVAWAHLDIAGTAYYASGFGVRCLIYYIEK
16080258_Bacillus_subt                REGHAIMAGTFLGEFAESTPWVHLDIAGTATGATGVMARTLATLAER
19074788_Encephalitozoon_cuni         GHGGSATAAIFLSEFVG-FKWAHFDIAGVMDGATGCGVPVLIEMIEK
19909972_Coprinopsis_cinere           RPAGSATAALFLKPFVNGIKWAHLDIAGSMEGMTGRPVRALVEFTRR
Dictyostelium_discoid                 SGGACCAAGFLKEFITAQSWSHLDIAGVMSGMTGKPTRTLIEFAKK-
19113905_Schizosaccharomyces_p        AGGGCCTAAAFIKCFLAQLSFAHLDIAGVMDGMSGRPVRTIIEVARK
_21355725_Drosophila_melano           GRGRPCKAAAFLREFVP-GQWMHIDATNVMVGMAGRPTRTLIEFIAQ
_7705688_Homo_sapien                  YRAGACTAAAFLKEFVT-PKWAHLDIAGVMTGMTGRPTRTLIEFLLR
29832567_Streptomyces_averm           RMGGGLVAGLFLREFVGEITWAHLDIAGPAFGGTGTAVRTLVRLAEL
21674006_Chlorobium_tepid             RGAGTITAAKFLEKFIDGKHWAHIDIAGPAFGATGFGVRLLLDLLKG
_5870602_Leishmania_major]            GRAGSCTAAAFLSNFVEPVQWAHLDIAGVADGVTGFGVQLLVDFLRH
                                              .. :   :
```

Glucose-6-phosphate isomerase (Fig. 4B)

CLUSTAL X (1.81) multiple sequence alignment

```
15595075_Borrelia               ETHLKIFQNLSDEANLIEKYKEVLSENRKVLHHLTRGQGKDVIDNKENMR
16752000_Chlamydophila          DAILAALISLAEERGLHESMLAMQSEMRPALHTATRAWTDSSFGEAEDIA
269_185_Cryptosporidium         EEGFELLIKLAAESNLMEKIKLQLTEKRAVLHTALRSKNIPITSSGQNVL
4835820_Toxoplasma              LETLQHLVNLAHERQVPAMVKRMFTENRAVLHVALRMPGSEPVVDGKNVL
23509563_Plasmodium             EKTLNKLVEYAEEVELKKKVEKTFTENRSVLHTALRIPNTHKIIDNKNVL
15239045_Arabidopsis            VETMDKLLNLAKASQLTEKISRMFTENRSVLHVALRAPKDAVIADGMNVV
1169797_Oryza                   GETMEKLFKLAEAAKLKEKIEKMFTENRSVLHVALRAPRDEVISNGVNVV
1169798_Oryza                   GETMEKLFKLAEAAKLKEKIEKMFTENRSVLHVALRAPRDEVISDGVNVV
1346073_Zea                     GETMEKLLKLADAAKLKEKIEKMFTENRSVLHVALRAPRDAVISDGVNVV
37960111_Phytophthora           SKTLDLLFELAETAELKKKLQAIATEDRAVMHMALRAPKTEKIVDGHDVV
16273471_Haemophilus            QTTLSHLRQLAQECALDSAKEAMFTENRAVLHTALRNRNTPVLVDGKDVM
15804618_Escherichia            EETLAKLQDLAKECDLAGAIKSMFTENRAVLHVALRNRNTPILVDGKDVM
23136626_Cytophaga              DETISLLIALAEEAGLKQAIEDMFTEKRSVLHVALRNRNTPILSDGKDVM
17510665_Caenorhabditis         DESFQLLMRLAKSRGVEESRNAMFTENRAVLHVALRNRNRPILVDGKDVM
17737445_Drosophila             DEVWDLLLTLAKVRRVNAARDAMFTENRAVLHTALRNRTDPVLVDDKDVM
18201905_Homo                   EDVMRMLVDLAKSRGVEAARERMFTEGRAVLHVALRNRNTPILVDGKDVM
Entamoeba                       EETMKLLIKLCEAVHLKEKIEAEFTEKRAVLHTALRNRNNPVLVDGKDVM
Dictyostelium                   KETMDLLFELARASKVEEMRNSMFTEDRAVLHTALRNRPSAVIVDGENVI
28923732_Neurospora             EETLDKLVRLAEEAGVEKKRDAMFTEDRAVYHVALRNVNQEMKVDGVDVM
6319673_Saccharomyces           DEIIAALIELAKEANVTGLRDAMFTEDRAVYHVALRNRNKPMYVDGVNVA
7437359_Trypanosoma             DEIKDVLFKLVEERGIRAFMRALFAENRSVLHIALRNRNRPIFVNGHDVM
15677249_Neisseria              DETMSLLFELAREAGVPERMRQMFTENRAVLHVALRNRNSPIVVDGEDVM
15612158_Helicobacter           DTTLKLLFELAKDCSLKEKIEAMFTEKRAVLHTALRSLDAEILLDNMEVL
21673816_Chlorobium             ARTMELLMDLVRRSGIEKKRRQMFTEHRSVLHTALRRPGYTMTIDGNDVA
15887753_Agrobacterium          EEIVTLLEQLAREGGVEAKREEMFTEDRAVLHTALRNRNTPVLVDDKDVM
15964236_Sinorhizobium          DRIIDGLEALAKAAKVEEKRDAMFTEERAVLHTALRNRNRPVLVDGKDVM
15806745_Deinococcus            DETLRLLRELAQATGVEARRDAMFTEGRAVLHTALRAPDAVIEVDGKNVV
37521261_Gloeobacter            DETLRLLSVLAEESDLRGRIEAMFTEQRSVLHTALRAPGATVIEDGENVV
29832844_Streptomyces           DETLRLLRELAAATDVFGLRDAMFTEDRAVLHTALRAPDAVVEVDGENVV
29828312_Streptomyces           DETLALLQELATATDVFGLRDAMFTENRAVLHTALRAPDAVIEVDGENVV
15608086_Mycobacterium          RETLALLIDLARTAHLEERRDQMFSEDRAVLHTALRLPDAELVVDGQDVV
17546438_Ralstonia              PETLALLLQLADEAGVLTLRDAMLTEHRSVLHAALRGHEDDYRANGAAVM
15676249_Neisseria              EDTLQLLCNLADAADLEGKMRALRSEGRAALHTALRLPGADAVVDGRDVL
27573133_Neisseria              EDTLQLLCRLAETADLEGKMRALRSEGRAALHTALRLPGADAVADGRDVL
19173604_Encephalitozoon        EEIVDGYLEKMKD--FGEKIDGMFTENRKVLHVALRDKVLRMVGHGDAKL
16330760_Synechocystis          DLQPKFAKAFQDMVALEKGAIANPDEQRMVGHYWLRNP----AAPNDGIR
23125809_Nostoc                 SLQPKFDKAFADMAELEKGAIANPDENRMVGHYWLRNP----DAPTPELT
18030019_Spironucleus           -YAKNFEIALAEFAAIEKGEIVNKSENRPVDHYNHRLT----QLVPGKSL
13924524_Giardia                LYKQAHAAAFDLMDRIEKLENVNVSEDRPVDHYNLRME----CVK-GRSL
18030022_Trichomonas            EYKPKVAQALELIKRMENGEIVNHSEDRMVDHYNLRME----KLVKGKSL
```

```
15644137_Thermotoga           LFEPNISGGLTDEDVKSVEEKVTS-------AVRNFVE----NPDFAKLD
16080187_Bacillus             EHELTYLRDFVKTAHHNIHEKTGA------SDFLGWVD----LEHYDKEE
15669801_Methanococcus        EISLEDINKVDFANAYSNLMEKLD-------------------VGFRDVI
23473348_Desulfovibrio        SNSAFLNWTHARHGRISGTDPASLGEAANLLGLELAAGRLPFIMPYADG-


15595075_Borrelia             EFFQSELEKIYNFAKQIHSGNIKSSNGKKFKNVVQIGIGGSSLGPKALYS
16752000_Chlamydophila        VRSRVEAQRLKDFLTKVRS---------QFTTIVQIGIGGSELGPKALYR
269_185_Cryptosporidium       NDVNEVNRRIFKFANAIRKGELLGSTGKILKDVICIGIGGSYLGPEFVYE
4835820_Toxoplasma            DEVHAVLRRIRVFSEKVRSGEIRGHTGKKLVNVISIGIGGSYLGTEFVHL
23509563_Plasmodium           EDVHGVLKKIEKYSDDIRNGVIKTCKNTKFKNVICIGIGGSYLGTEFVYE
15239045_Arabidopsis          PEVWNVLDKIKEFSDKIRSGSWVGATGKPLKDVIAIGIGGSFLGPLFVHT
1169797_Oryza                 PEVWGVKDKIKQFSETFRSGSWVGATGKALTNVVSVGIGGSFLGPLFVHA
1169798_Oryza                 PEVWGVKDKIKQFSETFRSGSWVGATGKALTNVVSVGIGGSFLGPLFVHA
1346073_Zea                   PEVWSVKDKIKQFSETFRSGSWVGATGKPLTNVVSVGIGGSFLGPLFVHT
37960111_Phytophthora         PDVHEVLDAIRAFTSNVRDGKLVGATGKQLKNVISIGIGGSYLGPEFVFE
16273471_Haemophilus          PEVNAVLAKMKDFCQRIISGEWKGYTGKAITDVVNIGIGGSDLGPYMVTE
15804618_Escherichia          PEVNAVLEKMKTFSEAIISGEWKGYTGKAITDVVNIGIGGSDLGPYMVTE
23136626_Cytophaga            PEVNAVLEKMKGFCEKVRSGAWKGYTGKSITDIVNIGIGGSDLGPVMVTE
17510665_Caenorhabditis       PDVNRVLAHMKEFCNEIISGSWTGYTGKKITDVVNIGIGGSDLGPLMVTE
17737445_Drosophila           PDVRAELAHMKEFTNMVISGVWRGCTGKQITDVVNIGIGGSDLGPLMVTE
18201905_Homo                 PEVNKVLDKMKSFCQRVRSGDWKGYTGKTITDVINIGIGGSDLGPLMVTE
Entamoeba                     PGVNAVLNKMGKFAEGVRNGSIKGYTGKEFTDIVNIGIGGSDLGPVMVTE
Dictyostelium                 PSVRKVLDKMRSFSERVRSGQWKGYTGKTITDVVNIGIGGSDLGPVMVTQ
28923732_Neurospora           NTVNEVLQHMKEFSEQVRSGEWKGYTGKKLTNIINIGIGGSDLGPVMVTE
6319673_Saccharomyces         PEVDSVLKHMKEFSEQVRSGEWKGYTGKKITDVVNIGIGGSDLGPVMVTE
7437359_Trypanosoma           PLVNKVLEQMKKLSEKVRRGEWKGQSGKPIRHVVNIGIGGSDLGPMMACE
15677249_Neisseria            PKVNRVLQRMGEFAHEVRSGSWLGYTNQVITDVVNIGIGGSDLGPLMMCT
15612158_Helicobacter         KSVRSVLKRMRAFSDSVRSGKRLGYTNQVITDIVNIGIGGSDLGALMVCT
21673816_Chlorobium           SEVSDVLDQMKAFCKKVISGEWKGYTGKRITDVVNIGIGGSDLGPFMVTE
15887753_Agrobacterium        PDVNGVLAAMGKFADAIRSGSLKGATGKKITDVVNIGIGGSDLGPVMATL
15964236_Sinorhizobium        PDVNAVLEAMGKFADDIRSGALKGATGKKITDVVNIGIGGSDLGPVMATL
15806745_Deinococcus          PEVHEVLDRMATFADAVRSGEWLGYTGKPIKNIVNIGIGGSDLGPVMAYE
37521261_Gloeobacter          PEVHAVLDRMAEFADRVRGGEWRGYTGRRIRTVVNIGIGGSYLGPDMAYD
29832844_Streptomyces         PAVHAVLDKMSTFAERVRTGEWTGHTGKPIKNIVNVGIGGSDLGPAMAYE
29828312_Streptomyces         PAVHAVLDRMADFADRVRSGEWTGHTGSRIRTVVNIGIGGSDLGPAMAYE
15608086_Mycobacterium        TDVHAVLDAMGAFTDRLRSGEWTGATGKRISTVVNIGIGGSDLGPVMVYQ
17546438_Ralstonia            PDVLRVRAQMRDFAQRVHSGTWTGHAGQRITDVVNIGIGGSDLGPRMVCR
15676249_Neisseria            PEIRRELNRALKFAHSLDDGSYQGITGKRITDFVHIGIGGSDLGPAMCVQ
27573133_Neisseria            PEIRRELNRALKFAHSLDDGLYQGITGKRIADFVHIGIGGSDLGPAMCVQ
19173604_Encephalitozoon      DEVYDELMKIKAFVEDFDSGRVCGVTGKKLEIVVNIGIGGSDLGPRMVCD
16330760_Synechocystis        AEITEPLRQIKAFVADVHQGNIKPPTAPKFTDLLAIGIGGSALGPQFVAQ
23125809_Nostoc               QEIVQTLEQIEAFAEKVQTGAIHPPRASRFTDIISIGIGGSALGPQFVAE
18030019_Spironucleus         EHSLKLWEKVNTFVSSIRN---------DYKHIIFNGIGGSYLGPYMLIQ
13924524_Giardia              AKSIALWNTVKAFAKKCIDA-------KDYDHVIYNGIGGSYLGPYLLLT
18030022_Trichomonas          AHTLAMWEEAKKFAEDVMTGVIKTSAGKKYESIIFNGIGGSYLGPLMLII
15644137_Thermotoga           RSWIDSVKSLEDWII-------------NFDTVVVLGIGGSGLGNLALHY
16080187_Bacillus             FARIQ--KSAEKIQS-------------DSDVLLVVGIGGSYLGARAAIE
15669801_Methanococcus        YD--ENLDKYKSLNG--------------YENVVVIGMGGSILGTMAIYY
23473348_Desulfovibrio        --MTARLQELEPLLR-------------RFEHMVVLGIGGSALGARALQK
                                                              .:  *:*** **

15595075_Borrelia             SIKNGYFISNIDPDESEEVLSSINVDETLFIIVSKSGNTLETKANMQFLI
16752000_Chlamydophila        ALRHVHFISNIDPDNGAEVLDTIDCAKALVVVVSKSGTTIETAVNEAFFA
269_185_Cryptosporidium       ALRRLRFLANVDPIDIRRATEGLHPETTLVIIVSKTFTTAETILNAKTIK
4835820_Toxoplasma            ALAQIHFLANVDPVDVWLAERGFDPEETLVVVISKTFTTAETMMNARSVR
23509563_Plasmodium           AMKNVRFLANVDPNDVNRAIQNLDQYDTLVIIISKTFTTAETMLNARSIK
15239045_Arabidopsis          ALQQLRFLANIDPVDVARNISGLNPETTLVVVVSKTFTTAETMLNARTLR
1169797_Oryza                 ALQQLRFLANVDPVDVARSIKDLDPETTLVVVVSKTFTTAETMLNARTLK
1169798_Oryza                 ALQQLRFLANVDPVDVARSIKDLDPETTLVVVVSKTFTTAETMLNARTLQ
1346073_Zea                   ALQQLRFLANVDPVDVARSIKDLDPETTLVVVVSKTFTTAETMLNARTLK
37960111_Phytophthora         SLRNLRFLANVDPVDVARATSGLNPEETLVVVVSKTFTTAETMLNARTLR
16273471_Haemophilus          ALRNMHFVSNVDGTHIAETLKKVNPETTLFLVASKTFTTQETMTNAQSAR
15804618_Escherichia          ALRNMHFVSNVDGTHIAEVLKKVNPETTLFLVASKTFTTQETMTNAHSAR
23136626_Cytophaga            ALKHVHFVSNVDGTHIVETTKKLNPETTLFIIASKTFTTQETITNAESAK
17510665_Caenorhabditis       SLKNVHFVSNVDGTHVAEVTKKLNAETTLFIIASKTFTTQETITNAETAK
17737445_Drosophila           ALKHSHFVSNIDGTHLAEVLKKVNYETTLFIVASKTFTTQETITNATSAK
18201905_Homo                 ALKRVWYVSNIDGTHIAKTLAQLNPESSLFIIASKTFTTQETITNAETAK
Entamoeba                     ALKTLHYVSNVDGTHMVEALKKCNPETTLFVICSKTFTTAETLMNAHSAR
Dictyostelium                 ALKRAHFVSNIDGTHLAETVKHLCPETTLFIVASKTFTTQETITNAQSAR
28923732_Neurospora           ALKTLRFVSNVDGTHIAEALAASDPETTLFLIASKTFTTAETITNANTAK
6319673_Saccharomyces         ALKDVHFVSNIDGTHIAETLKVVDPETTLFLIASKTFTTAETITNANTAK
7437359_Trypanosoma           ALRSMHFVSNIDGTHLSEVLNLVDLESTLFIIASKTFTTQETITNALSAR
```

```
15677249_Neisseria            ALKNMHFVSNVDGSQLRDVLSKVHPETTLFIIASKTFTTQETLTNALTAR
15612158_Helicobacter         ALKKMHFVSNVDGTQILDVLEKINPASTLFIVASKTFSTQETLTNALTAR
21673816_Chlorobium           ALKKVHFVSNVDGSHLVETLRGLNPETTLFIIASKTFTTQETLANAVSAR
15887753_Agrobacterium        ALARAHFVSNIDGAHIADTLKLLDPETSLFIIASKTFTTIETMTNAATAR
15964236_Sinorhizobium        ALARLHFVSNVDGAHIADTLTLLDPETSLFIVASKTFTTIETMTNAATAR
15806745_Deinococcus          ALKTVRFVSNVDGTDLTEKTRDLDPEVTLFIVSSKTFTTQETMTNARSAR
37521261_Gloeobacter          ALKKVRFAANVDGSNFAEVIHDLEPDETLFIVCSKTFTTLETMTNAHSAR
29832844_Streptomyces         VLRTVRFVSNVDGADLHEAVRDLDPAETLFVIASKTFTTIETITNATSAR
29828312_Streptomyces         ALRTVRFVSNVDGADLHEAVRDLDPAETLFVIASKTFTTIETITNATSAR
15608086_Mycobacterium        ALRSARFVSNVDPADLIATLADLDPATTLFIVASKTFSTLETLTNATAAR
17546438_Ralstonia            ALARVHFVSNVDGTDLAETLAGLNPDTTLAIVCSKTFTTLETMANAHSMR
15676249_Neisseria            ALETVHFAANADPACLDAVLCRLNPETTVFCVASKSFKTPETLLNAQAVK
27573133_Neisseria            ALESVHFVSNADPACLDEVLCRLNPETTMFCVASKSFKTPETLLNAEAVK
19173604_Encephalitozoon      ALGETYFISNIDATDTIRVFEKIDPERALFIVVSKTFTTLETIKNAELAM
16330760_Synechocystis        ALAAIHFIDNSDPDGIDRVLNCLKAKSTLVVTTSKSGGTPEPRNGLAETK
23125809_Nostoc               ALAKIHFIDNNDPAGIDRVLNHLR-ASTLVLVISKSGGTPEPRNGMIEVK
18030019_Spironucleus         AML-LHFLANTDSDSFSTLFDLLD-KKTLMVTISKSGSTAETATNTQCYL
13924524_Giardia              AKY-AHFVANTDPASFAQVIELVTEERTLMVVISKSGGTAETATNCATYL
18030022_Trichomonas          AKYKIYFILQHRFRHVSTRSHPTS-MLPSWSTFPSLAPHPRQLVTHSTWM
15644137_Thermotoga           SLRRVFVVDNVDPDLMSSVLDRIDPKTTLFNVISKSGSTAEVMATYSIAR
16080187_Bacillus             ALNVIFIGNNISSSYMRDVMDLLEDVDFSINVISKSGTTTEPAIAFRIFR
15669801_Methanococcus        AIS-AYFIDNSDPEKTLSILKKVDLNESIIYIISKSGNTLETLVNYYLIK
23473348_Desulfovibrio        AFFWLWIADNVDADSLEAWLQSLPAEKTVVVVISKSGGTIETIGQYFLIR
                                       :                       ..     .

15595075_Borrelia             NKLKLNGIYKKQMVIITLKDSMLAIEEKGYLEYFFMHDSIGGRFSPTSAV
16752000_Chlamydophila        DYFAKKGLFKDHFIAVTCEGSPMDDTGK-YLEVFHLWESIGGRFSSTSMV
269_185_Cryptosporidium       EWLHKALKVSKHLAAVSTNIKATSDFGIPVDHVFGFWDWVGGRFSVCSAV
4835820_Toxoplasma            DWYLHHYKLGAHFCAVSTNLDGTSKFGIQSDRVFGFWDWVGGRYSVTSAV
23509563_Plasmodium           KWLSLKIKLSKHMVAVSTNLKLTDEFGISRDNVFEFWDWVGGRFSVTSSV
15239045_Arabidopsis          EWITAALGVAKHMVAVSTNLALVEKFGIDPNNAFAFWDWVGGRYSVCSAV
1169797_Oryza                 EWIVSSLGVAKHMIAVSTNLELVEKFGIDPKNAFAFWDWVGGRYSVCSAV
1169798_Oryza                 WSGLSLLLVAKHMIAVSTNLELVEKFGIDPKNAFAFWDWVGGRYSVCSAV
1346073_Zea                   EWIVSSLGVAKHMIAVSTNLKLVKEFGIDPNNAFAFWDWVGGRYSVCSAV
37960111_Phytophthora         KWLVDGLTVAKHMVAASSAVPLVQQFGIDRANIFGFWDWVGGRYSVTSAV
16273471_Haemophilus          DWLLKAAKVAKHFAALSTNAKDVEKFGIDTNNMFEFWDWVGGRYSLWSAI
15804618_Escherichia          DWFLKAAGVAKHFAALSTNAKAVGEFGIDTANMFEFWDWVGGRYSLWSAI
23136626_Cytophaga            AWFLQTAKVAKHFVALSTNAKSVAAFGIDTENMFEFWDWVGGRYSLWSAI
17510665_Caenorhabditis       EWFLAKAGVAKHFVALSTNVTKAVEFGIDEKNMFEFWDWVGGRYSLWSAI
17737445_Drosophila           TWLLEHSKVAKHFVALSTNKEKVTEFGIDSTNMFGFWDWVGGRYSLWSAI
18201905_Homo                 EWFLQAAKVAKHFVALSTNTTKVKEFGIDPQNMFEFWDWVGGRYSLWSAI
Entamoeba                     KWFVEKTGVSKHFVAVSTNATEVSKFGINTDNMFEFWDWVGGRYSLWSAI
Dictyostelium                 SWFLEKIGIGQHFVALSTNEQEVTKFGILKENMFEFWDWVGGRYSVWSAI
28923732_Neurospora           SWFLEKTGITKHFVALSTNEAEVTKFGIDAKNMFGFESWVGGRYSVWSAI
6319673_Saccharomyces         NWFLSKTGIAKHFAALSTNETEVAKFGIDTKNMFGFESWVGGRYSVWSAI
7437359_Trypanosoma           NEFLKFLSVAKHFVALSTNAEKVKEFGIDEENMFQFWDWVGGRYSLWSAI
15677249_Neisseria            EWFLNHAGVAKHFAAVSTNQKAVAEFGIDTANMFEFWDWVGGRYSLWSAI
15612158_Helicobacter         KWFVERSGIAKHFVAVSTNKEAVQQFGIDEHNMFEFWDFVGGRYSLWSAI
21673816_Chlorobium           AWFLVKAGVAKHFVAVSTNREKVEEFGIDPDNMFRFWDWVGGRYSLWSAI
15887753_Agrobacterium        RFIAEKLGVKHHFAAVSTALDKVAAFGIESDRIFGFWDWVGGRYSIWSAI
15964236_Sinorhizobium        AFIAGKLGVGHHFAAVSTALDKVGAFGIDAARVFGFWDWVGGRYSIWSAI
15806745_Deinococcus          KWLLGSLKVTRHFVAVSTNAEEVQKFGIDTANMFGFWDWVGGRYSMDSAI
37521261_Gloeobacter          QWCLAALGIAKHFVAVSTNAAEVEKFGIDTAHMFGFWDWVGGRYSMDSAI
29832844_Streptomyces         DWLLTELKVAKHFVALSTNAEKVSEFGIDTANMFEFWDWVGGRYSFDSAI
29828312_Streptomyces         SWLLAGLDVAKHFVALSTNAGKVSDFGIDTANMFEFWDWVGGRYSFDSAI
15608086_Mycobacterium        RWLTDALGVSRHFVAVSTNKRLVDDFGINTDNMFGFWDWVGGRYSVDSAI
17546438_Ralstonia            RWFI-EHGLKQHFVAVSTNRDAVVAFGIDPDNMFTFWDWVGGRFSLWSAV
15676249_Neisseria            AWYRGAGFTACHFCAVSADTAAAAAFGIAAERVFAMYDWVGGRYSVWSPV
27573133_Neisseria            AWYRGAGFTARHFCAVSADTEAAQSFGIAAERVFAMYDWVGGRYSVWSPV
19173604_Encephalitozoon      KLLERKLGASKHFVAVSSNVQEVGKHNIS--RVFSMWDFVGGRFSLWSAV
16330760_Synechocystis        AVFEAQGLYAVAVTMPGSK-LSQQAQTEQWLQAFPMQDWVGGRTSELSAV
23125809_Nostoc               KAYAGHNLYAVAITSVDSN-LDKLAKDEGWLARFPMYDWVGGRTSEMSAV
18030019_Spironucleus         NLLTKASLHCIAITIPGSN-LHKLAAS--WLQTFEMCEPTGGRTSICSAV
13924524_Giardia              TLLKDLRVSFVAVTTEGSK-LDKQANEQQFRETFHMNEATGGRTSVCSAV
18030022_Trichomonas          KLCRDRNLHNAAVTIKDSL-LDKIAHENKFIRTWHMEIDTGGRTSVCSAI
15644137_Thermotoga           GILEAYGLEHMLITTDPEKGFLRKLVKEEGFRSLEVPPGVGGRFSVLTPV
16080187_Bacillus             KLLEEKYGARIYATTDKERGALKTLSNEEGFESFVIPDDVGGRYSVLTAV
15669801_Methanococcus        KRIEKLNSFKGKLVFITNGGKLKREAEKNNYDIFSIPENVPGRFSVFTAV
23473348_Desulfovibrio        DWLRTARGNHVIAVTDERSGFLREEADTHGLASMPVPDHLGGRYSVLSAV
                                                                 .     ** *  : :

15595075_Borrelia             GLLTLCFTEKVAKEILKGANEADKKSLNKNVKNASLLAALISIYERNVLN
16752000_Chlamydophila        GVLGFAYGFEVFLQLLQGASAMDQIALQPNARNLPMLSALISIWNRNFLG
```

```
269_185_Cryptosporidium       GPLSIHFGANLVQEFLDGCWDMDQHYETAPVSNLPVLLGLLSVYNSTFMD
4835820_Toxoplasma            GPLALQYGYDVAQEFLNGAHAMDVHFKTAELANLPMLMGLISVWNATFFG
23509563_Plasmodium           GPLSIAFGYKNMRNFLNGCHDMDEHFLHADLKNIPVLLALTSFYNSHFFD
15239045_Arabidopsis          GPLSLQYGFSMVEKFLKGASSIDQHFQSTPFENIPVLLGLLSVWNVSFLG
1169797_Oryza                 GPLSLQYGFPIVQKFLEGAASIDKHFRSSSFENIPVLLGLLSVWNVSFLG
1169798_Oryza                 GPLSLQYGFPIVQKFLEGAASIDKHFRSSSFENIPVLLGLLSVWNVSFLG
1346073_Zea                   GPLSLQYGFPIVQKFLEGASSIDNHFYSSSFENIPVLLGLLSVWNVSFLG
37960111_Phytophthora         GPLALQYGFDITEKFLAGAHAMDKHLLETPLRNLPVIMGLLGVWNSSFLG
16273471_Haemophilus          GSIALSIGFENFEALLNGAHEMDKHFLSTPIENIPTTLALVGLWNTNFLG
15804618_Escherichia          GSIVLSIGFDNFVELLSGAHAMDKHFSTTPAENLPVLLALIGIWYNNFFG
23136626_Cytophaga            GSIALYIGYDNYIELLSGAHAMDKHFKETPFANIPVLLALIGIWYNNFYK
17510665_Caenorhabditis       GSIAVHIGFDNYEKLLDGAFSVDEHFVNTPLENIPVILAMIGVLYNNIYG
17737445_Drosophila           GSICLSIGFENFEQLLDGAHFMDNHFKTTPFENAPVILALLGVWYSNFFK
18201905_Homo                 GSIALHVGFDNFEQLLSGAHWMDQHFRTTPLENAPVLLALLGIWYINCFG
Entamoeba                     GTIMISIGEQGFLDLLSGAHAMDKHFRNTEFANIPVILAVLGVWYNNLYQ
Dictyostelium                 GSVALYVGMDHFESFLEGAYHMDQHFLNTPLENLPVIMGLLGVWYNNFFG
28923732_Neurospora           GSVALYVGYENFHKFLAGAHAMDNHFRTAPLKNIPVLGGILSVWYSNFYN
6319673_Saccharomyces         GSVALYIGYDNFEAFLKGAEAVDNHFTQTPLENIPLLGGLLSVWYNNFFG
7437359_Trypanosoma           GSVMISIGYDNFVELLTGAHIMDEHFINAPTENLPIILALVGIWYNNFFG
15677249_Neisseria            GPIMLYLGEENFIEMLNGAHLMDQHFINTPLENLPVILALIGIWYINYYG
15612158_Helicobacter         GSIMIYLGKKNFNALLKGAYLMDEHFRNAPFENLPVLMGLIGVWYINFFQ
21673816_Chlorobium           GSIALYLGFDRFRELLAGAHAMDEHFLNAPLENMPMILAMLGIWYNNFFG
15887753_Agrobacterium        GPLMIAIGPDNFGKFLDGAHAMDRHFREAPIRNLPMLLGLIGFYNRNVLD
15964236_Sinorhizobium        GPLMIAIGKENFGRFLDGGHAIDEHFRSAPLRNIPMLLGLIGFYNRNVLG
15806745_Deinococcus          GSLMVAVGPEHFREMLAGFHDMDEHFRTAPAENLPMLMGLLGVWYGDFFG
37521261_Gloeobacter          GSTMIAVGPEHFRAMLAGFHAMDEHFRTAPFENLPVLMALIGLWYNNFFG
29832844_Streptomyces         GSLMIAIGPERFREMLDGFRIVDEHFESAPAENVPLLLGLLGIWYGNFHD
29828312_Streptomyces         GSLMIAIGPERFRELLDGFRIVDEHFRTAPAENAPLLLGLLGVWYGSFFG
15608086_Mycobacterium        GSLMTVIGRDAFADFLAGFHIIDRHFATAPLENAPVLLGLIGLWYSNFFG
17546438_Ralstonia            GSIVLAIGPEQFETMLDGARAMDRHFASAAPRNMPLILGLLSVWYRGFFG
15676249_Neisseria            GPVMVAVGGARFRELLAGAHAMDRHFFSTPTRNIPVLMALIAVWYNNFQH
27573133_Neisseria            GPVMVAVGGARFRELLAGAHAMDSHFFHTPPRNIPVLMALIAVWYNNFQH
19173604_Encephalitozoon      GSIALYVGFNNFMRLLKGASAVDEDFRRNRGRNAEMIHAIAELFYS-ENG
16330760_Synechocystis        GLLPAALQGIDIQAMLDGAKTMDEATRVRELRQNPAALLALAWYYAGDGQ
23125809_Nostoc               GLVPAALQGIDVRAMLDGAKEMDDATRVPDVKNNPAALLALSWYFAGNGK
18030019_Spironucleus         AMVPCAFAGIKFDEFLKGMAQMDCETRKAEGN-PALEIAAMIDSLIKKFA
13924524_Giardia              GMVPAAFAGVDFESFLIGMSEMDKLTRSHRENNPAIAYATLLDWMLKDKS
18030022_Trichomonas          GFVPYAFARCDFGEFIKGMSYMDTLTRAEGEN-PAALLATAIDANN-HKV
15644137_Thermotoga           GLLSAMAEGIDIDELHEGAKDAFEKSMKENIL-ENPAAMIALTHYLYLNK
16080187_Bacillus             GLLPIAVSGVNIDDMMKGALDASKDFATSELE-DNPAYQYAVVRNVLYNK
15669801_Methanococcus        GLAPLYSLGVDISKILEGAREMDKICQNEDILKNPALLNGVIHYLYDKRG
23473348_Desulfovibrio        GLIPAAFTGMDWQALVRGAKTVGAPLCSSPDAGAHPAWHLAAWNHALMQA
                              .             :  *

15595075_Borrelia             YSSNCIIAYSKAMENFYLHLQQLEMESNGKSVNRFNETINTVRIIWGGIG
16752000_Chlamydophila        YPTEAVIPYSSGLEFFPAHLQQCCMESNGKSIVQDGRRVGTSPVIWGEPG
269_185_Cryptosporidium       KSCVAVLPYCQALCKFPAHVQQLLMESNGKSSSIDGNMLHTGAIFFGEPG
4835820_Toxoplasma            YSNVAVLPYAQALLRFPAHIQQLTMESNGKRVTMDGKTLDVGEIFFGEPG
23509563_Plasmodium           YKNVAILPYFQNLLKFSAHIQQLSMESNGKSVDRNNQPIHTCQVYFGEPG
15239045_Arabidopsis          YPARAILPYSQALEKFAPHIQQVSMESNGKGVSIDGLPLPTGEIDFGEPG
1169797_Oryza                 YPARAILPYSQALEKFAPHIQQLSMESNGKGVSIDGVQLSTGEIDFGEPG
1169798_Oryza                 YPARAILPYSQALEKFAPHIQQLSMESNGKGVSIDGVQLPSGEIDFGEPG
1346073_Zea                   YPARAILPYSQALEKLAPHIQQLSMESNGKGVSIDGAQLSTGEIDFGEPG
37960111_Phytophthora         HSSRALLPYSQALLRFAAHIQQVDMESNGKRVTVEGVDLPAGEVNFGEPG
16273471_Haemophilus          AQTEAILPYDQYLHRFAAYFQQGNMESNGKYVDRDGNVIKTGPIIWGEPG
15804618_Escherichia          AETEAILPYDQYMHRFAAYFQQGNMESNGKYVDRNGNVVDTGPIIWGEPG
23136626_Cytophaga            AESHAILPYDQYMHRFAAYFQQGDMESNGKRVTKDGTAVTTGPIIWGEPG
17510665_Caenorhabditis       AETHALLPYDQYMHRFAAYFQQGDMESNGKFVTRHGQRVDTGPIVWGEPG
17737445_Drosophila           AETHALLPYDQYLHRFAAYFQQGDMESNGKFVSKSGKPVKTGPIVWGEPG
18201905_Homo                 CETHAMLPYDQYLHRFAAYFQQGDMESNGKYITKSGTRVDTGPIVWGEPG
Entamoeba                     AQSHAILPYDQYLHRFAAYFQQGDMESNGKRITKDGKVVNTGPIIWGEPG
Dictyostelium                 CQTQAILPYDQYLSRFPAYFQQGDMESNGKSVQRNGERVTTGPIIWGEPG
28923732_Neurospora           AQTHLIAPFDQYLHRFPAYLQQLSMESNGKSITSDGSAAKTGPIVFGEPC
6319673_Saccharomyces         AQTHLVAPFDQYLHRFPAYLQQLSMESNGKSVTRGNVFTDTGSILFGEPA
7437359_Trypanosoma           SETQAILPYDQYLWRLPAYLQQLDMESNGKGATKNGRMVSTGPIIFGEAG
15677249_Neisseria            GGSHVIAPYDQHLHRLPKFIQQLDMESNGKQVTLDGKAVGTSPIIWGETG
15612158_Helicobacter         SKSHLIAPYDQYLRHFPKFIQQLDMESNGKRISKKGEIIPTCPVVWGDMG
21673816_Chlorobium           AHSQAIIPYDQYLHRFPAYLQQLDMESNGKRVDRAGHEVDTGPVIWGEPG
15887753_Agrobacterium        YPTRAILPYDQRLSRFPAYLQQLDMESNGKGVTIDGTPVESGPVVWGEPG
15964236_Sinorhizobium        YPSRAILPYDQRLTRFPAYLQQLDMESNGKGVTLDSQPVETGPVVWGEPG
15806745_Deinococcus          AESLAVLPYDQYLASFPAYLQQLDMESNGKHVTLGGEPVDTGPIVWGQAG
37521261_Gloeobacter          AQTLAVLPYDYYLGKLPAYLQQLDMESNGKHVDIDGQPVTTGPIIWGQPG
29832844_Streptomyces         AQSHAVLPYSHYLSKFTAYLQQLDMESNGKSVQRDGNPVETGPVVWGTPG
```

```
29828312_Streptomyces        AQSHAVLPYSHYLSKFTAYLQQLDMESNGKSVDRDGHPVETGPVVWGTPG
15608086_Mycobacterium       AQSRTVLPYSNDLSRFPAYLQQLTMESNGKSTRADGSPVSTGEIFWGEPG
17546438_Ralstonia           AASACTVPYCAPLELLTDFMQQLEMESNGKSVQRNGAAIGTGPIVWGTAG
15676249_Neisseria           ADGQTAVPYSHNLRLLPAWLNQLDMESLGKSRASDGSPAVTGGIVFGGEG
27573133_Neisseria           ADGQTAVPYSHNLRLLPAWLNQLDMESLGKSRASDGSPAATGGIVFGGEG
19173604_Encephalitozoon     FNNKCIVCYDQYMEKFYLYLQQAEMESNGKQSERG-----TGLIVWGGLG
16330760_Synechocystis       GKKDVILPYKDRLLLFSRYLQQLVMESLGKERDLDGNVVHQGIAVYGNKG
23125809_Nostoc              GEKDVVLPYKDSLFLFSRYLQQLVMESLGKEKDLDGNVVHQGIAVYGNKG
18030019_Spironucleus        APKNIILGYSESLKQYAHYCQQLYMESLGKEYCKDGRIQPSGLSVYGGIG
13924524_Giardia             TPFNILLAYSDPLKHLSHYCQQLFMESLGKNYTREALPLKTGLTILGGTG
18030022_Trichomonas         GHRNIVLCYNEFMREYAHYLQQLYMESLGKQYKVDGTEARQGQTVFGGVG
15644137_Thermotoga          GKSIVMMAYSNRMIYLVDWYRQLWAESLGKRYNLKGEEVFTGQTPVKALG
16080187_Bacillus            GKTIMLINYEPALQYFAEWWKQLFGESEGKDE--------KGIYPSSANY
15669801_Methanococcus       KDISVIMSYVESLKYFGDWYKQLIGESLGKNKHGITPLLS--------IG
23473348_Desulfovibrio       GYSQIFFSYMPLWAHFGFWFAQLWAESLGKEG--------KGSMPLPAVG
                                     :            *   ** **

15595075_Borrelia            TDVQHSFFQMLHQGTIVPMDFIGFDKLKANLIAQIIAFSKGKENSNKNKN
16752000_Chlamydophila       TNGQHSFFQCLHQGTIIPVEFIGFQKLFANMIAQAIALACGSENTNPNKN
269_185_Cryptosporidium      TNAQHSFYQLLHQGRTTNCEFIGFDELMCNFFAQPDALAIGKENLIPHKL
4835820_Toxoplasma           TNGQHSFYQLIHQGRVIPAEFIGFDELMSNFFAQPDALAFGKEKLVPHKT
23509563_Plasmodium          TNGQHSFYQLIHQGQVIPVELIGFDELMTNFFAQADALAIGKPELLTHKV
15239045_Arabidopsis         TNGQHSFYQLIHQGRVIPCDFIGIDELMSNFFAQPDALAYGKENLIPHKT
1169797_Oryza                TNGQHSFYQLIHQGRVIPCDFIGVDELMSNFFAQPDALAYGKEHLIPHKT
1169798_Oryza                TNGQHSFYQLIHQGRVIPCDFIGVDELMSNFFAQPDALAYGKEHLISHKT
1346073_Zea                  TNGQHSFYQLIHQGRVIPCDFIGVDELMSNFFAQPDALAYGKENLIPHKT
37960111_Phytophthora        TNGQHSFYQLIHQGRVVPCDFLGFDELMSNFFAQPDALARGKEKLQNHKL
16273471_Haemophilus         TNGQHAFYQLIHQGTLIPCDFIAPNKLLSNFFAQTEALAFGKKNIVPFKV
15804618_Escherichia         TNGQHAFYQLIHQGTMVPCDFIAPQKLLSNFFAQTEALAFGKDYVVPFKV
23136626_Cytophaga           TNGQHAFYQLIHQGTLIPCDFLAPPKLLANFFAQTEALMKGKAALVNHKV
17510665_Caenorhabditis      TNGQHAFYQLIHQGTLIPADFIAPQILLANFLAQTEALMKGKAKILPHKV
17737445_Drosophila          TNGQHAFYQLIHQGTLIPCDFIAPKILLSNFLAQTEALMAGKDNLLPHKV
18201905_Homo                TNGQHAFYQLIHQGTMIPCDFLIPKILLANFLAQTEALMRGKERLLPHKV
Entamoeba                    TNGQHAFYQLIHQGTLIPADFIIPTVLLANFIAQTEALMMGKKELLPHKL
Dictyostelium                TNGQHAFYQLLHQGQIIPCDFIASQILLSNFFAQTEALMKGKKQLLPHKV
28923732_Neurospora          TNAQHSFFQLVHQGTLIPADFILAKMLASNYLAQAEALMVGKEHLVPHKV
6319673_Saccharomyces        TNAQHSFFQLVHQGTLIPSDFILAKMLASNFFAQAEALMVGKGGLVPHKV
7437359_Trypanosoma          TNGQHAFYQLIHQGTLIPCDFIGARILMSNFFAQTEALMIGKQLLIPQKT
15677249_Neisseria           INGQHAFFQLLHQGTITPIDLIASEILLANVFAQAEAFMRGKEELVPHKT
15612158_Helicobacter        INAQHAFFQLLHQGTLIPIDFIASEILFSNVLAQAQAFMKGKKDLAHHRV
21673816_Chlorobium          TNAQHAFFQLLHQGTIVPVDFIVSDMLVANCFAQSEALMKGKEKLLPHKL
15887753_Agrobacterium       TNGQHAFYQLIHQGTVIPAEFMIAQLLIANCLAQSEALMKGRDFIAPHRV
15964236_Sinorhizobium       TNGQHAFYQLIHQGTVIPAEFMIAQLLMANCLAQSEALMKGRDKIAPHRV
15806745_Deinococcus         TNGQHAFYQLIHQGTLIPCDFIGFDLLMANVFAQTEALAFGKPEVAPHRV
37521261_Gloeobacter         TDGQHSFYQLIHQGTLIPCDFIGFDQLMANFFAQTEALAFGKDWLLPHRV
29832844_Streptomyces        TNGQHAYYQLIHQGTLIPADFIGFDLLMANFFAQTQALAFGKEQLVPHKT
29828312_Streptomyces        TNGQHAYYQLLHQGTVIPADLIGFDLLMANLFAQGQALAFGKEEQVPHRT
15608086_Mycobacterium       TNGQHAFYQLLHQGTLVPADFIGFDLLMSNFFAQTQVLAFGKAHVVAHKV
17546438_Ralstonia           TNGQHAYFQLIHQGSIVPVDFITTTKLLANCFAQGEALLRGRAALVPHMV
15676249_Neisseria           VNCQHAYFQLLHQGTLIPCDFIVPRFTVANAFAQAEALMKGKERLAPHKE
27573133_Neisseria           VNCQHAYFQLLHQGTLIPCDFIVPRFTVANAFAQAEALMKGKERLAPHKE
19173604_Encephalitozoon     TNTQHSFFQLLHQGTDVLVELLMPNMVVSNCLAQSRGLMVGK------ES
16330760_Synechocystis       STDQHAYVQQLRDGVNFFATFIEVHDRQGPSLELEPGVTSGDLQGTRQAL
23125809_Nostoc              STDQHAYVQQLREGVNFFATFVEVEDRQGPSTEIDPGVTSGDLQGTRQAL
18030019_Spironucleus        TGEQHAFMQQIQKGVDSFVKFIQFERKHEDYEDSKAGSMG-RLKGTELAL
13924524_Giardia             TGEQHAFMQQIQKGCDAAVQMIRCKR-AADYPDEAAGSMG-RVKGTEMAL
18030022_Trichomonas         TGEQHSFMQQVQKGIDNFVRMIYFKR-EHDYVNEQAGSMG-RLMGTQKAL
15644137_Thermotoga          ATDQHSQIQLYNEGPDKVITFLRVNFDREIVIPETG--RAELQTGTEEAL
16080187_Bacillus            STDLHSLGQYVQEGRDLFETVLNVKPKHELTIEEADNDLDGLFQGTMLAH
15669801_Methanococcus       AKDQHSLLQLYMDGKDKIITFMVAEIEFEDINDEKISCRYSD-KATEIAL
23473348_Desulfovibrio       VTDQHSTQQMFLDGPDKACLFLTCGRTFPGDMPPKWDFLRGRALGTRMAL
                                 *:  *   .*      .:

15595075_Borrelia            FQGERPSALIYSKELTPYAIGAILSHYENKVMFEGFLLNINSFDQEGVQL
16752000_Chlamydophila       FDGNRPSSVLVSSQLNPYSLGELLSYYENKIVFQGFCWGINSFDQEGVSL
269_185_Cryptosporidium      FQGNRSSISLLLPICSAYYIGQLLALYEHRTAVEGFILNINSFDQYGVEL
4835820_Toxoplasma           FPGDRPSCMLLFPEISPFHIGQLLALYEHRVAVEGWLWGINSFDQWGVEL
23509563_Plasmodium          FNGNRPSTLLLFDELNFYTCGLLLSLYESRIVAEGFLLNINSFDQWGVEL
15239045_Arabidopsis         FSGNRPSLSLLLPELTAYNVGQLLAIYEHRVAVQGFVWGINSFDQWGVEL
1169797_Oryza                FQGNRPSLSLLLPSLSAYEIGQLLAIYEHRIAVQGFLWGINSFDQWGVEL
1169798_Oryza                FQGNRPSLSLLLPSLSAYEIGQLLSIYEHRIAVQGFLWGINSFDQWGVEL
1346073_Zea                  FKGNRPSLSLLLPTLSAYEVGQLLSIYEHRIAVQGFIWGINSFDQWGVEL
37960111_Phytophthora        FPGNRPSISLLFKKLDAFSTGQLLALYEHRTVVQGAIWGINSFDQWGVEL
```

```
16273471_Haemophilus          FTGNKPTNSILVQKITPFTLGALIAMYEHKIFVQGVIFNIFSFDQWGVEL
15804618_Escherichia          FEGNRPTNSILLREITPFSLGALIALYEHKIFTQGVILNIFTFDQWGVEL
23136626_Cytophaga            FEGNRPTNSIFFKKLTPFTLGSLIAMYEHKIYTQSVVWQINAFDQWGVEL
17510665_Caenorhabditis       FEGNKPTTSIVLPVVTPFTLGALIAFYEHKIFVQGIIWDICSYDQWGVEL
17737445_Drosophila           FVGNRPTNSIVVKKVSPFTLGALIALYEHKIFVQGIIWDINSFDQWGVEL
18201905_Homo                 FEGNRPTNSIVFTKLTPFMLGALVAMYEHKIFVQGIIWDINSFDQWGVEL
Entamoeba                     FPGNRPTNSIVFKKMTPYVLGSLIAMYEHKIFVQGCIWNVNSFDQWGVEL
Dictyostelium                 FEGNRPTNSIFLHKLTPHSLGALIALYEHKIFVQGIIWNINSFDQWGVEL
28923732_Neurospora           FLGNRPTTSILVGHIGPAELGALIVYYEHLTFTEGAIWDINSFDQWGVEL
6319673_Saccharomyces         FSGNRPTTSILAQKITPATLGALIAYYEHVTFTEGAIWNINSFDQWGVEL
7437359_Trypanosoma           FTGGRPSNSLLVKALTPRALGAIIAMYEHKVLVQGAIWGINSYDQWGVEL
15677249_Neisseria            FSGNRPTNLILMDKVNPRNMGSLIAMYEHKTFVQGIIWGINSFDQWGVEL
15612158_Helicobacter         FFGNRPSNILLLEKISPSNIGALVALYEHKVFVQGVIWDINSFDQWGVEL
21673816_Chlorobium           FPGNRPTNTIVLDELNPFNLGSLIALYEHKVFVQGVVWNINSFDQWGVEL
15887753_Agrobacterium        FTGNRPSITFVYDKLTPFALGRLIALYEHRVFVEGVLFRINSFDQWGVEL
15964236_Sinorhizobium        FTGNRPSLTIVYDQLDPFALGRLIALYEHRVFVEGALFNINSFDQWGVEL
15806745_Deinococcus          FEGNRPTSTILADRLTPRTLGALIALYEHKVFVQGAVWDINSFDQWGVEL
37521261_Gloeobacter          FEGNRPTNTLLAERLTPEVLGKLIALYEHKVFTQGVIWNLDSFDQWGVEL
29832844_Streptomyces         FKGNHPTTTILAKELTPSVLGQLIALYEHKVFVQGAVWNIDSFDQWGVEL
29828312_Streptomyces         FRGNHPTTTILAAELTPSVLGQLIALYEHKVFVQGAIWNIDSFDQWGVEL
15608086_Mycobacterium        MPGNRPSTSILASRLTPSVLGQLIALYEHQVFTEGVVWGIDSFDQWGVEL
17546438_Ralstonia            FEGNRPSNTILMQRLDAASLGALIACAEHRTFVQGAVWNINSFDQWGVEL
15676249_Neisseria            FPGNRPSNSILIDRLTPYNLGMLMAAYEHKTFVQGAIWNVNPFDQWGVEY
27573133_Neisseria            FPGNRPSNSILLDRLTPYNLGMLMAAYEHKTFVQGAIWNVNPFDQWGVEY
19173604_Encephalitozoon      FRGNRPTITVCYSKLTPETLGAMIAHYEHKIFILGLYWGINSFDQPGVTL
16330760_Synechocystis        FENQRDSITVTIPEVDATSVGALIALYERAVSFYGSLVNVNAYHQPGVEA
23125809_Nostoc               YENHRDSITVTIPQVNPRTVGALIALYERAVGLYASLVNVNAYHQPGVEA
18030019_Spironucleus         SQNGREFMTITVKKCSEFCIGQLIALEERIVSTLGAFWGINAYDQPGVQD
13924524_Giardia              YQNERAFVCITIDGFCERCIGQLIAFEERVVTILSAFWNINAFDQPGVQD
18030022_Trichomonas          IQNKRSFATSTFRERNEFTFGMMIALEERVVTFLASFFGINAYDQPGVQD
15644137_Thermotoga           RENNRPNMRVTFDGLTPYNVGQFFAYYEAATAFMGYLLEINPFDQPGVEL
16080187_Bacillus             TDGNVPNLIVNIPELNAYTFGYLVYFFEKACAMSGYLLGVNPFDQPGVEA
15669801_Methanococcus        TNNGVPNVRITLDEINEMAMGALLYMYEMQVGFMGELYNINAYNQPAVEE
23473348_Desulfovibrio        HKTGTPLVEIGMAQPSEEQAGRLMALLEITTLLTGWLLDINPLDQPAVEL
                                                  * :.   *      .    : . .* .*

15595075_Borrelia             GKIIANQILKNDN
16752000_Chlamydophila        GKALANRVLELLE
269_185_Cryptosporidium       GKVLAKDIRNIIS
4835820_Toxoplasma            GKVLAKGVRGILQ
23509563_Plasmodium           GKVLAKEVRNYFN
15239045_Arabidopsis          GKVLATQVRKQLH
1169797_Oryza                 GKSLASQVRKSLH
1169798_Oryza                 GKSLASQVRKSLH
1346073_Zea                   GKSLASQVRKQLH
37960111_Phytophthora         GKVLAKQVRAQLS
16273471_Haemophilus          GKQLANRILPELT
15804618_Escherichia          GKQLANRILPELK
23136626_Cytophaga            GKQLANAILPELK
17510665_Caenorhabditis       GKQLAKVIQPELA
17737445_Drosophila           GKQLAKAIEPELD
18201905_Homo                 GKQLAKKIEPELD
Entamoeba                     GKQLAKAVEKDLE
Dictyostelium                 GKQLAKSILPELS
28923732_Neurospora           GKVLAKKILKEID
6319673_Saccharomyces         GKVLAKVIGKELD
7437359_Trypanosoma           GKLLAKSILLQLQ
15677249_Neisseria            GKQLAKTILGELT
15612158_Helicobacter         GKELAVPILQELE
21673816_Chlorobium           GKQLAKAILPEFD
15887753_Agrobacterium        GKELATGLLPVVE
15964236_Sinorhizobium        GKELATGLLPVIE
15806745_Deinococcus          GKVLAKKIDGELQ
37521261_Gloeobacter          GKVLAGRIIPELE
29832844_Streptomyces         GKVLAKRVEPALT
29828312_Streptomyces         GKVLAKRVEPALT
15608086_Mycobacterium        GKTQAKALLPVIT
17546438_Ralstonia            GKKLAKPILEELE
15676249_Neisseria            GKQLAKTIIGELE
27573133_Neisseria            GKQLAKTIIGELE
19173604_Encephalitozoon      GKKIATEVLETLE
16330760_Synechocystis        GKKAAASILELQK
23125809_Nostoc               GKKAAASILDLQT
```

```
18030019_Spironucleus         GKKAATRVNELSM
13924524_Giardia              GKLACLVCNKLSK
18030022_Trichomonas          GKKAATGVNATSK
15644137_Thermotoga           GKKITFALMGR--
16080187_Bacillus             YKVNMFALLGK--
15669801_Methanococcus        EKKICWRLIKQ--
23473348_Desulfovibrio        GKRLANARLGAPG
                               *
```

Tryptophan synthetase β- chain (Fig. 4c)

CLUSTAL X (1.81) multiple sequence alignment

```
34557127_Wolinella_succin          FGKYGGAYLPPLLIEHFKEIGEAYLKISQSFDFIQELKSIRKHYQGRPTP
222_2_Cryptosporidium_parvum       FGKYGGKYVPEVINNAMKEIEDAYNKISKSEDFINELKKIRKEFQGRPTP
22958844_Rhodobacter_sphaer        FGPYGGAMLPPPLEPHFAAIREAYGRISKSADFIAELRYIRKHFQGRPTP
28900440_Vibrio_paraha             FGEYGGSFVPPELEQIMRDINAAYEECCQDPEFKDELARLYKHFVGRPSP
33456993_Cycloclasticus_sp         FGEYGGQVIPPDLVAIMNDINRAYQEISKTPSFKDELNDLYTHYVGRPSP
15614226_Bacillus_halod            FGTFGGKYVPETLMSAIEELELALNDAMNDEAFIHDYKEHLREYAGRPTA
16079321_Bacillus_subti            YGDFGGKFVPETLMQPLDEIQTAFKQIKDDPAFREEYYKLLKDYSGRPTA
14520675_Pyrococcus_abyssi         FGKFGGQYVPETLMEPLRELEKAYKRLKNDEEFNRQLDYYLRTWAGRPTP
11499193_Archaeoglobus_b2          FGEFGGRFVPEVLIPPLEELEKAYDRFKDDEEFKARLEYYLKSYAGRPTP
15606106_Aquifex_trpB1             FGPFGGKFVPETLMYALEELEEKYRELKSDPEFQKELDYYLREYAGRPTP
20808007_Thermoanaerobacter_te     FGRFGGQYVPETVMNALIELEREFEEAKKDEKFMEEYRYYLKEYSGRPTP
15673445_Lactococcus_lactis        YGQFGGQFVPETLMTAVKQLEEAYVDSKKDPLFQAELKELLKDYVGRENP
32417954_Neurospora_crassa         FGEFGGQYVPEALMDCLSELEEGFNKIKDDPAFWEEYRSYYP-WMGRPGQ
24215988_Leptospira_interr         FGEFGGRYSPEILHDALVELETTYKKLKKNKHFKKELEYYRKNYIGRPSP
2822116_Coprinopsis_cinerea        FGQFGGQYVPESLVDALAELEEAHKSAIEDPAFWEEVRSLYT-YSNRPSN
15642912_Thermotoga_maritima       FGPYGGQYVPEILMPALEELEAAYEEIMKDESFWKEFNDLLRDYAGRPTP
29345943_Bacteroides               YGEFGGAYVPEILHKCVEELKNTYLGVLESEDFKKEFDQLLRDYVGRPSP
34557707_Wolinella_succin          FGKFGGRFVPETLMPALLELESAYNELRFDREFWSEVDYYLKEYVGRPSP
15963781_Sinorhizobium_melilot     FGIFGGRFVAETLMPLILDLQDEWARAKNDPAFKAELENLGTHYIGRPSP
23502958_Brucella                  FGIFGGRFVAETLMPLILELQQAYETAKNDPEFKAELNALSTFYAGRPSK
22957700_Rhodobacter_sphaer        FGIFGGRFVSETLMPLILDLEARYEHAKTDPDFWAEMDDLWKNYVGRPSP
464937_Pseudomonas_syringae        FGAFGGRYVAETLMPLILDLAREYEAAKEDPAFKEELAYFQRDYVGRPSP
23053570_Geobacter_b1              FGQFGGRYVPETLMPALLELEKAYNHYKNDREFKEEFDYYLRQYVGRPNP
17546702_Ralstonia_solana          FGPYGGTFVAETLSHALDELRDAYARYQHDPEFIKEYEYELKHFVGRPSP
Leishmania_sanger_major            -----------------------YANLRTDAEFEAEFRRELKHFVGRPSP
1174780_Zea_cp2                    FGRFGGKYVPETLMHALTELESAFHALATDDEFQKELDGILKDYVGRESP
1174778_Zea_cp1                    -GRFGGKYVPETLMHALTELENAFHALATDDEFQKELDGILKDYVGRESP
7437014_Oryza_sativa               FGTFQGKYVGETLMHALSELESAFKKLATDDDFQRELAGILKDYVGRLSP
15236977_Arabidopsis_b2            FGKFGGKYVPETLMHALSELETAFYSLATDEDFQRELAEILKDYVGRESP
15239755_Arabidopsis_b1            FGKFGGKYVPETLMHALSELESAFYALATDDDFQRELAGILKDYVGRESP
3915165_Camptotheca_cp_b2          FGKFGGKYVPETLMYALTELESAFRSLSGDQVFQKELDGILKDYVGRESP
17231286_Nostoc                    FGRFGGKYVPETLMPALAELETAYQQYRNDPGFQAELQQLLRDYVGRATP
17227906_Nostoc_sp                 FGKFGGKYVPETLMPALGELETAYQKYRDDASYQTELQNLLRDYVGRPSP
33239640_Prochlorococcus           FGRFGGQYVPETLIPALTELEQAAATAWKDESFTKELTQLLKTYVGRETP
7437011_Chlamydomonas              YGQFGGKYVPETLIPALEQLEKDYNEAIADPAFKAEMEAILKDYVGRETP
4325276_Gracilaria_cp              FGRFGGQYVPETLMSCLQELEASYHRIKEDPEFLQELGGLLRDYVGRPSP
136282_Thermus_thermo              FGPYGGRYVPETLIPALEELEAAYREAKKDPAFLEELDHYLRQFAGRPTP
15805965_Deinococcus_radio         YGRFGGRYVPETLIPALDELEQAYEAAKQDPEFLDELDRLLREFVGRPNS
29832719_Streptomyces_avermi       FGAFGGKFIPEALVAAVDEVAVEYDKAKADPEFARELDDLLVHYTGRPSA
21673356_Chlorobium_tepid          FGTFGGKFIPETLVKNAADLEEEYLKAKNDPEFHQTLDNLLRHYVGRPTP
22974734_Chloroflexus_aurant       FGPYGGRYVPETLMPAVSALEEAYTAAIADPAFWAELNRLHRTYTGRPTP
28898734_Vibrio_paraha             FGEYGGQYVPQILVPALKQLEQAFIDAQEDPEFRSEFMTLLQEYAGRPTA
924784_E_coli                      FGEFGGMYVPQILMPALRQLEEAFVSAQKDPEFQAQFNDLLKNYAGRPTA
16273336_Haemophilus_influ         FGEFGGMYVPEILVPVLKQLEQAFVEAQNDPTFQAEFADLLKNYAGRPTA
15612264_Helicobacter_pylori       FGEFGGSFVSELLVPALRELEQAFDTCLKDEEFQKEYFRLLKDFVGRPSP
23473438_Desulfovibrio_desulf      FGDFGGQFVPELLMPPLKELENALETVVPSPEFQNEFHMLLREYVGRETP
23473141_Desulfovibrio_desulf      --------MPDSLKPILDELAATFERYRNDPGFLEEFNYYLTRYSGRETP
19703662_Fusobacterium_nuclea      FGEFGGSYVPEVVQKALDKLEEAYNKYKDDEEFLKEYHHYLKNYSGRETP
29840324_Chlamydophila_caviae      PYPFGGQFMPEILMAPVQDLSNSWKSLQNHSDFKNELDSVLKNYAGRPTP
29840318_Chlamydophila_caviae      LETYGGQYVPLELVKPLEDLDRSYEQLKKDPQFRETFHHILKNYAGRPTP
14520795_Pyrococcus_abyssi         DPETEEPIDIEKLKRIFAEELVKQEISRERYIEIPGELRKLYSKIGRPTP
14520795_Pyrococcus_b2             DPETEEPIDIEKLKRIFAEELVKQEISRERYIEIPGELRKLYSKIGRPTP
18313363_Pyrobaculum               DPDEGE-SRIGLLSKILPSALIDQEFTAERWVSIPEEVREAYRRVGRPTP
15606594_Aquifex_trpB2             DPETMEPVKPEKLLAIFPEPLVEQEVSDKEWIDIPEEVLDIY-SLWRPTP
11498839_Archaeoglobus_b1          HPATQEPVKPEDLEPIFPKGLIQQEMSGERWIRIPEDVREIY-RIWRPTP
15643305_Thermotoga_maritima       DPETKQPISPEKLSVIFPMSLIEQEVSEERFIEIPEPVLKEY-AVYRPTP
23053561_Geobacter_b2              NPRTLQPVTPDDLLPIFPMAIIEQEVSGERWIPIPEEVREIY-RLWRPSP
21673033_Chlorobium_tepid          G-LDGNPIGPDALAKVFPMNLIEQEVSTERWIDIPEEILGIL-KLWRPSP
29350072_Bacteroides               HPGTKQPLKAEDLYPIFAKELCHQELNQDAWIEIPEDVREMY-KYYRSTP
```

```
                                                                                  *

34557127_Wolinella_succin           LYYARRLSQKAGAIYLKREDLNHTGAHKLNHCMAEALLAKHLGKKKLIAE
222_2_Cryptosporidium_parvum        IYYAKNLTKKYGEIYLKREDLNHTGAHKLNHCMGEALLAKYMGKKKLIAE
22958844_Rhodobacter_sphaer         VSHLKNLSALCGQIYAKREDLNHTGAHKLNHCMAEGLLARFMGKTKLMAE
28900440_Vibrio_paraha              IFHAANLSKKYGDIYLKREDLNHTGAHKINHCLGEAILAKKMGKKKLIAE
33456993_Cycloclasticus_sp          IFYAKRLTEKLGKIFLKREDLNHTGAHKINHCLGEALLARHMGKTKVIAE
15614226_Bacillus_halod             LTYAKNMSETLGKIYLKREDLLHTGAHKLNNAMGQALLAKRMGKKKIVAE
16079321_Bacillus_subti             LTYADRVTEYLGKIYLKREDLNHTGSHKINNALGQALLAKKMGKTKIIAE
14520675_Pyrococcus_abyssi          LYYAERLTKKVGKIYLKREDLLHGGAHKTNNAIGQALLAKFMGKTRLIAE
11499193_Archaeoglobus_b2           LYFAENLSRELGKIYLKREDLLHGGAHKINNTIGQALLAKFMGKKRVIAE
15606106_Aquifex_trpB1              LYFAEKLTKYVGKIYLKREDLLHTGAHKINNTIGQCLLTKRMGKKRVIAE
20808007_Thermoanaerobacter_te      LYYAENLTKRLGKIYLKREDLNHTGAHKINNVLGQVLLAKRMGKKRIIAE
15673445_Lactococcus_lactis         LYYAKRLTEYAGKIYLKREDLNHTGAHKINNALGQVLLAKKMGKNKVIAE
32417954_Neurospora_crassa          LHKAERLTEYAGNIWLKREDLNHTGSHKINNALGQLLLARRLGKKKIIAE
24215988_Leptospira_interr          LTYAERLSKVWGKIWLKREDLNHTGAHKINNTIGQVLIAKAMGKTRIIAE
2822116_Coprinopsis_cinerea         LYLAENLTKEAGNIWLKREDLNHTGSHKINNALGQILLAKRIGKTRIIAE
15642912_Thermotoga_maritima        LYFARRLSEKYGRIYLKREDLLHTGAHKINNAIGQVLLAKKMGKTRIIAE
29345943_Bacteroides                LYLARRLSEKYGKMYLKREDLNHTGAHKINNTIGQILLARRMGKKRIIAE
34557707_Wolinella_succin           LYYAERLSDELGKIYLKREDLNHTGAHKINNTVIQGLLAKRLGKKKIIAE
15963781_Sinorhizobium_melilot      LYFAERLTAELGKIYFKREELNHTGSHKINNCIGQILLAKRMGKTRIIAE
23502958_Brucella                   LYYAEGLSKHLGKIYFKREDLNHTGSHKINNCLGQILLAKRMGKTRIIAE
22957700_Rhodobacter_sphaer         LYFAPRLTEHLGKIYLKRDELNHTGAHKINNVLGQIILARRMGKTRIIAE
464937_Pseudomonas_syringae         LYFAERLTEFCGKIYLKREELNHTGAHKINNCIGQILLARRMGKKRIIAE
23053570_Geobacter_b1               LYFAEKLTQKLGKIYLKREDLNHTGAHKVNNTIGQGLLAKRMGKKRVIAE
17546702_Ralstonia_solana           IYHARRLTEHCGQIYLKREDLNHTGAHKVNNVIGQALLARRMGKPRVIAE
Leishmania_sanger_major             IYHAQRWSETLGQIYFKREDLNHTGAHKINNVIGQALLARKMGKPRVIAE
1174780_Zea_cp2                     LYFAERLTEHYKLIYLKREDLNHTGAHKINNAVAQALLAKRLGKQRIIAE
1174778_Zea_cp1                     LYFAERLTEHYKLIYLKREDLNHRGAHKINNAVAQALLAKRLGKQRIIAE
7437014_Oryza_sativa                LYFVESLTEHYRLIYLKREDNNHTGAHKINNAVAQALSAKRLGKKRIIAE
15236977_Arabidopsis_b2             LYFAERLTEHYRLIYLKREDLNHTGAHKINNAVAQALLAKRLGKKRIIAE
15239755_Arabidopsis_b1             LYFAERLTEHYRLIYLKREDLNHTGAHKINNAVAQALLAKRLGKKRIIAE
3915165_Camptotheca_cp_b2           LYFAERLTLHYKEIYLKREDLNHTGAHKINNAVAQALLAKRLGKKRIIAE
17231286_Nostoc                     LYFAERLTAHYAQIYLKREDLNHTGAHKINNALGQVLLAKRMGKQRIIAE
17227906_Nostoc_sp                  LYFAERLTEYYAQIYLKREDLNHTGAHKINNALAQVLLAKRIGKQRVIAE
33239640_Prochlorococcus            LYEATRLTKHYERIWLKREDLNHTGAHKINNALGQALLAIRMGKKRIIAE
7437011_Chlamydomonas               LYHAERLSAHYKEIYLKREDLNHTGAHKINNSLGQALLCKRLNKQRIIAE
4325276_Gracilaria_cp               LYFAERLTSRYAQIYLKREDLNHTGAHKINNSLAQVLLAKRLGKTRIIAE
136282_Thermus_thermo               LYHAKRLSEYWGQVFLKREDLLHTGAHKINNTLGQALLARRMGKRRVIAE
15805965_Deinococcus_radio          LYLAERLTEHAGKIYLKREDQNFTGAHKINNCLAQALLAKRMGKQKIIAE
29832719_Streptomyces_avermi        LTEVSRFAEHAGRVFLKREDLNHTGSHKINNVLGQALLTRRMGKTRVIAE
21673356_Chlorobium_tepid           LYHASRLSEKQGQIWLKREDLCHTGAHKINNALGQVLLAKRMGKKRIIAE
22974734_Chloroflexus_aurant        LSFAARLTAHCGQIYLKREDLAHTGAHKINNALGQGLLAKRMGKRRVIAE
28898734_Vibrio_paraha              LTLTRNLTKGTKKLYLKREDLLHGGAHKTNQVLGQALLAKRMGKHEIIAE
924784_E_coli                       LTKCQNITAGTNTLYLKREDLLHGGAHKTNQVLGQALLAKRMGKTEIIAE
16273336_Haemophilus_influ          LTLCRNLTKGTKKLYLKREDLLHGGAHKTNQVLGQILLAKRMGKTRIIAE
15612264_Helicobacter_pylori        LTLCQNIVSNPKKLYLKREDLIHGGAHKTNQALGQALLAKKMGKTRIIAE
23473438_Desulfovibrio_desulf       LTLCRRLSAELGNLWLKREDLAHSGSHKINNTLGQALLTKHMGKPAMLAE
23473141_Desulfovibrio_desulf       LYLCANLTRRLGKIYLKREDLNHLGAHKVNNTIGQILLAKRMGKKKIIAE
19703662_Fusobacterium_nuclea       LYFAESLTNYLGKIYLKREDLNHLGAHKLNNVIGQILLAKRMGKKKVIAE
29840324_Chlamydophila_caviae       LTEIKNFAKAIHRIFLKREDLLHTGAHKINNALGQCLLAKHLGKTRIIAE
29840318_Chlamydophila_caviae       LTEVKNFSRAINRIFLKREDLLHTGAHKINNVLGQCLIAKFQGKTRVVAE
14520795_Pyrococcus_abyssi          LFRATNLEKLLGRIYFKYEGATVTGSHKINTALAQAYYAKKQGIERLVTE
14520795_Pyrococcus_b2              LFRATNLEKLLGRIYFKYEGATVTGSHKINTALAQAYYAKKQGIERLVTE
18313363_Pyrobaculum                LFRAEGLEKALGRIYYKYEGVLPVGSHKLNTALAQAYYAKADGAVEVATE
15606594_Aquifex_trpB2              LHRAKNLEEFLGKIFYKNESVSPPGSHKPNTAVAQAYYNKISGVKRLTTE
11498839_Archaeoglobus_b1           LVRAERLEKALKRIYFKYEGASPPGSHKPNTAVAQAYYNAKEGVERLTTE
15643305_Thermotoga_maritima        LIRATFLEEYLQRIYYKYEGVSPTGSHKPNTAIAQAYYNKIEGVKRLVTE
23053561_Geobacter_b2               LYRARRLEQALGKIYYKYEGVSPAGSHKPNSAIPQAFYNKQAGIRRLATE
21673033_Chlorobium_tepid           LYRARRLEAALGKIYYKNEGVSPAGSHKPNTAVAQAWYNREFGIKYLTTE
29350072_Bacteroides                LVRAYGLEKALGHIYFKNESVSPIGSHKLNSALAQAYYCKEEGVTNVTTE
                                    :            :: * :     *:** *  : :       .   : :*

34557127_Wolinella_succin           TGAGQHGVALATAAAYFGMECEIHMGEVDIAKEHPNVIRMKMLGAKVVPV
222_2_Cryptosporidium_parvum        TGAGQHGVALATAAAYFGLECEIHMGEVDVKKEYPNVIRMKILGAKVVCV
22958844_Rhodobacter_sphaer         TGAGQHGVALATAAAYFGMECEIHMGEIDIAKEAPNVTRMKLLGATVVPV
28900440_Vibrio_paraha              TGAGQHGVALATAAALVGLECDIYMGEVDIAKEHPNVVRMRILGANVIPA
33456993_Cycloclasticus_sp          TGAGQHGVALATACALVGIDCEIHMGEVDIEKERPNVTKMKILGCTLVPV
15614226_Bacillus_halod             TGAGQHGVATATIAARFGLECKVFMGEEDMERQALNVFRMELLGAEVVPA
16079321_Bacillus_subti             TGAGQHGVAPATVAAKFGFSCTVFMGEEDVARQSLNVFRMKLLGAEVVPV
14520675_Pyrococcus_abyssi          TGAGQHGVATAMAGALLGMKVDIYMGAEDVERQKMNVFRMKLLGANVIPV
11499193_Archaeoglobus_b2           TGAGQHGVATAMAAALLGLEAEIYMGAEDYERQKMNVFRMELLGAKVTAV
15606106_Aquifex_trpB1              TGAGQHGVATATASALFGLECVVYMGEEDAERQALNVFRMKLLGAKVEIV
```

```
20808007_Thermoanaerobacter_te      TGAGQHGVATATAAAMFGMECEIFMGEEDIKRQALNVFRMKLLGAKVTAV
15673445_Lactococcus_lactis         TGAGQHGVASATAAALFGMECTIYMGEEDVKRQSLNVFRMELLGAKVHSV
32417954_Neurospora_crassa          TGAGQHGVATATVCAKFGMECTVFMGAEDVRRQALNVFRMKLLGAKVVAV
24215988_Leptospira_interr          TGAGQHGVATATVGAMFQMETVVYMGEEDLRRQELNAIRMRMLGAKVVGV
2822116_Coprinopsis_cinerea         TGAGQHGVATATVCAKFGLECVIYMGAEDVRRQALKLFRIEMLGAWVIPV
15642912_Thermotoga_maritima        TGAGQHGVATATAAALFGMECVIYMGEEDTIRQKPNVERMKLLGAKVVPV
29345943_Bacteroides                TGAGQHGVATATVCALMDMECIVYMGKTDVERQHINVEKMKMLGATVIPV
34557707_Wolinella_succin           TGAGQHGVATATIAALLGLECEVFMGSKDTARQELNVFRMKLLSSKVQSV
15963781_Sinorhizobium_melilot      TGAGQHGVASATVAARFGLPCVVYMGATDVERQAPNVFRMKLLGAEVKPV
23502958_Brucella                   TGAGQHGVASATVAARFGLPCIVYMGATDVERQKPNVFRMKLLGAEVKPV
22957700_Rhodobacter_sphaer         TGAGQHGVATATVCAKFGLKCVVYMGAHDVERQAPNVFRMRLLGAEVVPV
464937_Pseudomonas_syringae         TGAGMHGVATATVAARFGLQCVIYMGTTDIERQQANVFRMKLLGAEVIPV
23053570_Geobacter_b1               TGAGQHGVATATIAALFGMECEVFMGEEDIRRQALNVFRMKLLGAKVTPV
17546702_Ralstonia_solana           TGAGQHGVATATIAARYGMECVVYMGSEDVRRQAANVYRMKLLGATVVPV
Leishmania_sanger_major             TGAGQHGVATATIAARFGMECVVYMGAEDVKRQAANVYRMKLLGATVVPV
1174780_Zea_cp2                     TGAGQHGVATATVCRRFGLQCIIYMGAQDMERQALNVFRMRLLGAEVRAV
1174778_Zea_cp1                     TGAGQHGVATATVCARFGLQCIIYMGAQDMERQALNVFRMKLLGAEVRAV
7437014_Oryza_sativa                TGAGQHGVATATVCASFGLECIIYMGAQDMERQALNVFRMKLLGAEVREV
15236977_Arabidopsis_b2             TGAGQHGVATATVCARFGLQCIIYMGAQDMERQALNVFRMRLLGAEVRGV
15239755_Arabidopsis_b1             TGAGQHGVATATVCARFGLECIIYMGAQDMERQALNVFRMRLLGAEVRGV
3915165_Camptotheca_cp_b2           TGAGQHGVATATVCARFGLQCVIYMGAQDMERQALNVFRMRLLGAEVRAV
17231286_Nostoc                     TGAGQHGVATATVCARFGLECVIYMGVHDMERQALNVFRMRLMGAEVRPV
17227906_Nostoc_sp                  TGAGQHGVATATVCARFGLDCVIYMGIHDMERQALNVFRMKLMGAEVRPV
33239640_Prochlorococcus            TGAGQHGVATATVCARFGLECIIYMGQEDMRRQSLNVFRMKLLGAKVRPV
7437011_Chlamydomonas               TGAGQHGVATATICARLGLKCIVYMGAKDMERQALNVFRMRLCGAEVRPV
4325276_Gracilaria_cp               TGAGQHGVATATVCARFGLQCIVYMGAKDMERQALNVFRMRLLGAEVRPV
136282_Thermus_thermo               TGAGQHGVSVATVAALFGLECVVYMGEEDVRRQALNVFRMKLLGAEVRPV
15805965_Deinococcus_radio          TGAGQHGVASATAAALLGLSCVVYMGEEDMRRQSLNVFRMQLLGAEVRPV
29832719_Streptomyces_avermi        TGAGQHGVATATACALFGLDCTIYMGEIDTQRQALNVARMRMLGAEVIAV
21673356_Chlorobium_tepid           TGAGQHGVATATVCALFGLDCIVYMGEEDIRRQAPNVARMKLLGTEVRPV
22974734_Chloroflexus_aurant        TGAGQHGVATATVCALLGLECVVYMGVDDMARQQPNVFRMRLLGAEVRGV
28898734_Vibrio_paraha              TGAGQHGVATALACALLGLKCRVYMGAKDVERQSPNVFRMKLMGAEVIPV
924784_E_coli                       TGAGQHGVASALASALLGLKCRIYMGAKDVERQXPNVFRMRLMGAEVIPV
16273336_Haemophilus_influ          TGAGQHGVATALACAMLDMPCRVYMGAKDVERQSPNVFRMRLMGAEVIPV
15612264_Helicobacter_pylori        TGAGQHGVATAIACALLNLKCVIFMGGKDIKRQEMNVFRMRLLGAEVREV
23473438_Desulfovibrio_desulf       TGAGQHGVATATAAAALGLECVVFMGAVDVERQALNVERMRLLGAEVVPV
23473141_Desulfovibrio_desulf       TGAGQHGVATAATAALMGMECTIYMGAEDVERQKLNVFRMQMMGARVVPA
19703662_Fusobacterium_nuclea       TGAGQHGVATAAAAAKFGMQCDIYMGALDVERQRLNVFRMEILGATVHAV
29840324_Chlamydophila_caviae       TGAGQHGVATATACGCLGLECVIYMGAKDIERQKPNVDKMNLLGAEVISV
29840318_Chlamydophila_caviae       TGAGQHGVALAAAAAYLGMECVIFMGETDINRQKPNVDRIRVLGAEVVSV
14520795_Pyrococcus_abyssi          TGAGQWGTALSLAGALLGLKVRVYMARASYQQKPYRKTLMRIYGAEVFPS
14520795_Pyrococcus_b2              TGAGQWGTALSLAGALLGLKVRVYMARASYQQKPYRKTLMRIYGAEVFPS
18313363_Pyrobaculum                TGAGQWGMAVSLAAALFGLRAVVFMTRSSYNSKRQRLTFMRAYGAVVYPS
15606594_Aquifex_trpB2              TGAGQWGSALSFATQFFDLQCRVYMVRVSYNQKPYRRILMETWKGEVIPS
11498839_Archaeoglobus_b1           TGAGQWGSALCFATKLFEMACTVYMVKVSFMQKPYRRVMMETWGGEVIPS
15643305_Thermotoga_maritima        TGAGQWGSALSYAGAKFGLEVKVFMVKVSYQQKPMRKYMMNLFGGKVTPS
23053561_Geobacter_b2               TGAGQWGSSLALACSMFGLECTVYMVKVSCTQKPYRKSMMQLWGANVIPS
21673033_Chlorobium_tepid           TGAGQWGSALAMSCKLIGIECKVFMVRISFDQKPFRKIMMNTWGAECIPS
29350072_Bacteroides                TGAGQWGAALSYAAKVFGLEAAVYQVKISYEQKPYRRSIMQTFGAQVTPS
                                    ****  * : .       :   :.    .   :  .   :.

34557127_Wolinella_succin           SFGERTLKEAVDSAFEAYLKDPANAIYAIGSVVGPHPFPKMVRDFQSVVG
222_2_Cryptosporidium_parvum        EFGDKTLKEAVDSAFEAYIKDIDNTFYAIGSVVGPHPFPKMVRDFQSIVG
22958844_Rhodobacter_sphaer         GFGGRSLKEAVDSCFQSYLAQADTALFAIGSVVGPHPFPMMVRDFQHVVG
28900440_Vibrio_paraha              THGRKTLKEAVDAAFEAYLKDPETQLYAIGSVVGPHPFPKMVRDFQSIIG
33456993_Cycloclasticus_sp          SRGTRTLKDAVDSAFEEYLKDPVNFIYAIGSVVGPHPFPMMVRDFQSIIG
15614226_Bacillus_halod             TSGSKTLKDATNEAIRYWVTHADDTFYLIGSVVGPHPYPQMVRDFQRIIG
16079321_Bacillus_subti             TSGNGTLKDATNEAIRYWVQHCEDHFYMIGSVVGPHPYPQVVREFQKMIG
14520675_Pyrococcus_abyssi          HTGSKTLKDAINEALRDWVATFEYSHYLIGSVVGPHPYPIIVRDFQSVIG
11499193_Archaeoglobus_b2           ESGSRTLKDAINEALRDWVESFEHTHYLIGSVVGPHPFPTIVRDFQAVIG
15606106_Aquifex_trpB1              KSGSRTLKDAINEALRDWVTNVESTHYVIGSVVGPHPFPMIVRDFQSVIG
20808007_Thermoanaerobacter_te      TSGTRTLKDAVNEAIRDWVTNIETTFYVIGSVVGPHPYPTMVRDFQRVIG
15673445_Lactococcus_lactis         TDGSRVLKDAVNAALRAWVAQVEDTHYVMGSVLGPHPFPQIVRDYQAVIG
32417954_Neurospora_crassa          EAGSRTLRDAVNEALRYWVVNLADTHYIIGSAIGPHPFPTIVRTFQSVIG
24215988_Leptospira_interr          SSGTATLKDATSEAMRDWALNVSNTHYIVGSSIGPHPFPTIVRDFQSVIG
2822116_Coprinopsis_cinerea         HSGSCTLKDAVNEAMRDWVTNLSTTHYLVGSAIGPHPFPTIVRDFQKVIG
15642912_Thermotoga_maritima        KSGSRTLKDAINEALRDWITNLQTTYYVIGSVVGPHPYPIIVRNFQKVIG
29345943_Bacteroides                TSGNMTLKDATNEAIRDWCCHPADTYYIIGSTVGPHPYPDMVARLQSVIS
34557707_Wolinella_succin           ESGSKTLKDAMNEAIRHWVTHARDTFYIIGTVAGPHPYPMMVRDFQSVIS
15963781_Sinorhizobium_melilot      TAGNGTLKDAMNEALRDWVTNVDSTYYLIGTAAGPHPYPEMVRDFQAVIG
23502958_Brucella                   SAGNGTLKDAMNEALRDWVTNVEDTYYLIGTAAGPHPYPELVRDFQSVIG
22957700_Rhodobacter_sphaer         TSGRGTLKDAMNDALRDWVTNVRDTFYCIGTVAGPHPYPAMVRDFQSIIG
464937_Pseudomonas_syringae         VAGTGTLKDAMNEALRDWVTNVEDTFYLIGTVAGPHPYPAMVRDFQAVIG
```

```
23053570_Geobacter_b1               TAGTATLKDAMNEAMRNWVTYIHNTFYVIGTVAGPHPYPVMVRDFQSVIG
17546702_Ralstonia_solana           ESGSRTLKDALNEAMRDWVTNVADTFYIIGTVAGPHPYPMMVRDFQAVIG
Leishmania_sanger_major             ESGSKTLKDALNEAMRDWVTNVENTFYIIGTVAG----------------
1174780_Zea_cp2                     HSGTATLKDATSEAIRDWVTNVETTHYILGSVAGPHPYPMMVREFHKVIG
1174778_Zea_cp1                     HSGTATLKDATSEAIRDWVTNVETTHYILGSVAGPHPYPMMVREFHKVIG
7437014_Oryza_sativa                HSGTATLKDATSEAIRDWVTNVENTHYILGSVAGPHPYPMMVREFHKVIG
15236977_Arabidopsis_b2             HSGTATLKDATSEAIRDWVTNVETTHYILGSVAGPHPYPMMVRDFHAVIG
15239755_Arabidopsis_b1             HSGTATLKDATSEAIRDWVTNVETTHYILGSVAGPHPYPMMVRDFHAVIG
3915165_Camptotheca_cp_b2           HSGTATLKDATSEAIRDWVTNVESTHYILGSVAGPHPYPMMVREFHAVIG
17231286_Nostoc                     AAGTGTLKDATSEAIRDWVTNVETTHYILGSVAGPHPYPMMVRDFHAVIG
17227906_Nostoc_sp                  EAGTGTLKDATSEAIRDWVTNVETTHYILGSVAGPHPYPMIVRDFHAIIG
33239640_Prochlorococcus            TAGTATLKDATSEAIRDWVTNVETTHYILGSVAGPHPYPMLVRDFHAVIG
7437011_Chlamydomonas               NSGTATLKDATSEAIRDWVTNVETTHYILGSAAGPHPYPMMVREFQSVIG
4325276_Gracilaria_cp               TSGSATLTNALSEAIRDWVTYPIETHYIVGSVAGPHPYPMIVRDFQACIG
136282_Thermus_thermo               AAGSRTLKDATNEAIRDWITNVRTTFYILGSVVGPHPYPMMVRDFQSVIG
15805965_Deinococcus_radio          TSGTATLKDATNEAIREWVTNVRDTFYILGSVVGPHPYPAMVRDFQSVIG
29832719_Streptomyces_avermi        KSGSRTLKDAINEAFRDWVANVDRTHYLFGTVAGPHPFPAMVRDFHRVIG
21673356_Chlorobium_tepid           TAGSRTLKDATSEAIRDWMNNPEETFYIVGSVIGMHPYPMMVRDFQSVIG
22974734_Chloroflexus_aurant        SSGSRTLKDAINEAMRDWVTNP-DSYYLLGSALGPHPYPTMVRDFQRVIG
28898734_Vibrio_paraha              HSGSATLKDACNEALRDWSGSYEDAHYLLGTAAGPHPFPTIVREFQRMIG
924784_E_coli                       HSGSATLKDACNEALRDWSGSYETAHYMLGTAAGPHPYPTIVREFQRMIG
16273336_Haemophilus_influ          QKGSCSLKDACCEAMRDWSANYETTHYLLGTAAGPHPFPTIVREFQKMIG
15612264_Helicobacter_pylori        NSGSATLKDAVNEALRDWASSYKDTHYLLGTSAGPHPYPTMVKTFQKMIG
23473438_Desulfovibrio_desulf       QSGTRTLKDAINEAMRFWIANIATHHYCFGTAAGPHPFPTLVRDLQSVIG
23473141_Desulfovibrio_desulf       TSGQQTLKEAVDEALAAWIREADTTFYLLGSAVGPHPFPLMVREFQSVIG
19703662_Fusobacterium_nuclea       EKGERTLKEAVDAAFKAWINNINDTFYVLGSAVGPHPYPSMVKDFQRVIS
29840324_Chlamydophila_caviae       NQGAQTLKDAVNEALRDWSKNYESTHYCLGSALGPSPYPEMVRNFQSVIS
29840318_Chlamydophila_caviae       KRGNSGLKEAVDAAIEDFIFKHDHTHFCIGSALGPYPYPKIVRDFQSVIS
14520795_Pyrococcus_abyssi          PSGKRFLKEDPNHPGSLGIAISEAIEDVLKARYSLGSVLNHVLMHQTVIG
14520795_Pyrococcus_b2              PSGKRFLKEDPNHPGSLGIAISEAIEDVLKARYSLGSVLNHVLMHQTVIG
18313363_Pyrobaculum                PSGRRHYR--PDHPGSLGIAISEAVEYVLSRKYLPGSVMEFVLLHQTVIG
15606594_Aquifex_trpB2              PSGRKYYEENPEHPGSLGIAISEAIEEAASTKYSLGSVLNHVLLHQTVIG
11498839_Archaeoglobus_b1           PSGRKILAENPDTPGSLGIAISEAIEDAAKTKYSLGSVLNHVLLHQTVIG
15643305_Thermotoga_maritima        PSGRKILSEDPDNPGSLGIAISEALEVAVSTKYSLGSVLNHVLLHQTVIG
23053561_Geobacter_b2               PSGRAILAHDPDSNGSLGIAISEAVEDAVSTNYALGSVLNHVCLHQTVIG
21673033_Chlorobium_tepid           PSGRRILEEDPDTPGSLGIAISEAIEQAVETRYALGSVLNHVMLHQTIIG
29350072_Bacteroides                PSGKDILTAHPTYQGSLGTAISEAIELAQMCKYTLGSVLSHVTLHQTIIG
                                      *

34557127_Wolinella_succin           AEAKEQFLEMLPDHIVACVGGGSNAMGIFSAFIVELWGVEPLGK-GKSGE
222_2_Cryptosporidium_parvum        IESREQFLEMLPDIVTACVGGGSNAMGIFSGFIVELICVEPLGK-GNKGE
22958844_Rhodobacter_sphaer         VEAREQFLEMLPDMVAACVGGGSNAMGIFSGFLVALYGIEPCGT-SSKGD
28900440_Vibrio_paraha              NEARVQFKEMLPNNLVACVGGGSNAMGLFSAFLVAIHGVEPAGRSLDKGE
33456993_Cycloclasticus_sp          KEARAQFLEELPDVITACVGGGSNAIGLFTAFLVDIVGVEPAGRGLETGE
15614226_Bacillus_halod             DEARAQFQEILPTEVIACVGGGSNAIGTFYPFLVKLIGVEAAGKGVETSQ
16079321_Bacillus_subti             EEAKDQLKRIMPDKVVACVGGGSNAMGMFQAFLVELIGAEAAGKGIDTPL
14520675_Pyrococcus_abyssi          REAREQILEALPDVIVACVGGGSNAMGIFYPFVVRLIGVEAGGKGIESGK
11499193_Archaeoglobus_b2           KEARRQIIEAMPDAIIACVGGGSNAMGIFHPFLVRLIGVEAGGEGIESGR
15606106_Aquifex_trpB1              RETKEQILQKLPDAIVACVGGGSNAMGIFYPFVVQLIGVEAGGYGLETGQ
20808007_Thermoanaerobacter_te      DETKEQILEKLPDYVVACVGGGSNAMGIFYPFIVKLVGVEAAGEGIETGK
15673445_Lactococcus_lactis         QEARAQFLEKLPDALVACVGGGSNSMGLFYPFVVAMYGVEAAGLGIDTPH
32417954_Neurospora_crassa          NETKQQMLEKLPDAVVACVGGGSNAVGMFYPFSVKLLGVEAGGDGVDTPR
24215988_Leptospira_interr          IESRKQFKKVLPNAVIACVGGGSNAIGMFYGFIVKLFGVEAGGYSSDPGS
2822116_Coprinopsis_cinerea         EEIKAQLKEVLPDVVVACVGGGSNAIGTFYDFIVRLVGVEAGGEGIDGHK
15642912_Thermotoga_maritima        EETKKQILEKLPDYIVACVGGGSNAAGIFYPFIVKLIGVEAGGEGLETGK
29345943_Bacteroides                EEIKKQLMEKHPDYLIACVGGGSNAAGTIYHYIVGIILAEAGGKGIETGM
34557707_Wolinella_succin           FEAKKQILEKLPDYVIACIGGGSNAAGMFARFLVRCIGIEAGGLGIESHH
15963781_Sinorhizobium_melilot      EEAKQQILEALPDLVVAAVGGGSNAIGIFHSFLVRIVGVEAGGKGLDGDE
23502958_Brucella                   TEARQQILEQLPDVIVAAVGGGSNAIGLFHPFLVKIVGVEAGGRGLEGEE
22957700_Rhodobacter_sphaer         REVRWQLAEQLPDTLVAAIGGGSNAMGLFHPFLVRIVGVEAGGKGVDDME
464937_Pseudomonas_syringae         KETRDQLQAQLPDSLVACIGGGSNAMGLFHPFLVEIIGVEAAGHGIETGK
23053570_Geobacter_b1               REAKAQHKKILPDVLVACVGGGSNALGLFYPFLVRMVGVEAAGHGIASGA
17546702_Ralstonia_solana           EECKVQMPELQPDAVIACVGGGSNAMGIFYPYIVQLIGVEAAGEGLESGR
Leishmania_sanger_major             --------------------------------------------------
1174780_Zea_cp2                     KETRRQAMDKKPDVLVACVGGGSNAMGLFHEFVVRLVGLEAAGHGVDTDK
1174778_Zea_cp1                     KETRRQAMHKKPDVLVACVGGGSNAMGLFHEFVVRLIGVEAAGHGVDTDK
7437014_Oryza_sativa                KETRRQALEKKPDVLVACVGGGSNAMGLFHEFVVRMIGVEAAGFGVDSVK
15236977_Arabidopsis_b2             KETRKQAMEKKPDVLVACVGGGSNAMGLFHEFVVRMIGVEAAGFGLDSGK
15239755_Arabidopsis_b1             KETRKQALEKKPDVLVACVGGGSNAMGLFHEFVVRMIGVEAAGFGLDSGK
3915165_Camptotheca_cp_b2           KETRKQALEKKPDVLVACVGGGSNAMGLFHEFVVRMIGVEAAGFGLDSGK
17231286_Nostoc                     QETRAQALEKLPDILLACVGGGSNAMGLFYEFVIKLIGVEAAGEGVNTEK
17227906_Nostoc_sp                  KETRVQCQEKLPDILLACVGGGSNAIGLFHEFIIRLIGVEAAGEGVDTDK
33239640_Prochlorococcus            EEAKKQCLEASPDVLLACVGGGSNAMGLFHPFIVRMIGVEAAGDGVHTGR
```

```
7437011_Chlamydomonas          RETKVQAQEKLPDIVMACVGGGSNAIGIFNEFIVRLIGVEAGGEGVNTTK
4325276_Gracilaria_cp          RETREQALEKKPDILLACVGG-----------------------------
136282_Thermus_thermo          EEVKRQSLELLPDALIAAVGGGSNAIGLFAPFAPKLIGVEAAGEGLSTGR
15805965_Deinococcus_radio     EEVKVQLQEKVPDAIVACVGGGSNAIGIFAPFAPRLIGTEAAGHGVDTGM
29832719_Streptomyces_avermi   VEARRQILERLPDAAVACVGGGSNAIGLFHAFIVRLIGCEPAGHGLETGE
21673356_Chlorobium_tepid      RETRQQVLDQLPDVIVACVGGGSNAIGMFYEFLVELIGVEAAGEGLEG-K
22974734_Chloroflexus_aurant   LEAREQILAELPDVVVACVGGGSNAIGIFHPFLVALRGVEAGGKGERLGE
28898734_Vibrio_paraha         EETKNQILARLPDAVIACVGGGSNAIGMFADFIVRLIGVEPAGKGIDTDQ
924784_E_coli                  EETKAQILERLPDAVIACVGGGSNAIGMFADFIVGLIGVEPGGHGIETGE
16273336_Haemophilus_influ     EETKRQILERLPDTVIAAVGGGSNAIGMFADFIVRLIGVEPAGKGIETGE
15612264_Helicobacter_pylori   DEVKSQILEKLPDYVIACVGGGSNAIGIFSAFLVKLIGVEPAGLGLETNK
23473438_Desulfovibrio_desulf  REARRQILEKLPHMVVACVGGGSNAIGAFHAFIVQLVGVEAAGSGEPGCY
23473141_Desulfovibrio_desulf  RELRAQMLEALPDYCIACVGGGSNAIGMFSGFIVRLVGVEPAGRGLEYGD
19703662_Fusobacterium_nuclea  QEARRQILEKLPDMIIACVGGGSNAIGAFAEFIVKLIGVEAAGKGLNTDR
29840324_Chlamydophila_caviae  LEVKSQLQEISPDLLIACVGGGSNAIGFFHHFIVKLVGVEAGGLGIESGH
29840318_Chlamydophila_caviae  LEVKSQIKEYDPDILIACVGGGSNAIGFFHHFIVKLVGVEGGGLGVESGK
14520795_Pyrococcus_abyssi     LEAKEQMEEFEPDVIIGCVGGGSNFAGLAYPFVYEFIAVEPKAAPTMTGV
14520795_Pyrococcus_b2         LEAKEQMEEFEPDVIIGCVGGGSNFAGLAYPFVYEFIAVEPKAAPTMTGV
18313363_Pyrobaculum           LEAVRQLPE-EPDVAVACVGGGSNFAGFTYPMITRFVAVESEAAPKLTGE
15606594_Aquifex_trpB2         LEAKKQMEEAYPDVIIGAVGGGSNFAGLSFPFLLKVLAVEPEACPTLTGE
11498839_Archaeoglobus_b1      LETKAQLEKVKPDVLIGCVGGGSNFAGLTYPFVLEIIAVEPAACPTLTGE
15643305_Thermotoga_maritima   LEIKKQLELIKPDILLGCHGGGSNFGGTILPFVIRFVACEPAACPSLTGN
23053561_Geobacter_b2          QEAKEQLALAYPDVVIACCGGGSNFAGTAFPFLVRCLAVEPASCPTLTGV
21673033_Chlorobium_tepid      LEARKQFDKIYPDIVIGCAGGGSNFAGISFPFLVQVIATEPEACPTLTAP
29350072_Bacteroides           LEAEKQMEMAYPDVVIGCFGGGSNFGGISFPFMTRFVAAEPASCPKLTGK


34557127_Wolinella_succin      HAASLSYGKEGSIMLQNEDGSPASVHSVASGLDYPSVGPEHAYLHEIGRT
222_2_Cryptosporidium_parvum    HAASITYGSEGSIMLKDEEGNPSKVHSIASGLDYPSVGPEIAYLNSIGRT
22958844_Rhodobacter_sphaer    HAATIAYGEDGTMVLKDAEGNPAPVHTVASGLDYPGVGPEHAFLHRSGRV
28900440_Vibrio_paraha         HAATLTLGEPGSYMLKDEQGEPQEVHSVASGLDYPSVGPQHSYLKDIGRV
33456993_Cycloclasticus_sp     HAATMSKGVKGCYNLQDQSGEPLPVYSIASGLDYPGVGPQHCLLKDLGRA
15614226_Bacillus_halod        HAATITKGRVGTYLLQDDAGQITEPYSISAGLDYPGVGPEHAYLASTGRV
16079321_Bacillus_subti        HAATISKGTVGTYLIQDEFGQIIEPYSISAGLDYPGIGPEHAYLHKSGRV
14520675_Pyrococcus_abyssi     HSASLNAGEIGSYFLQDEEGQIRTTHSIAPGLDYPGVGPEHAYLKESGRA
11499193_Archaeoglobus_b2      HSASLTAGSKGSYFLQDEEGMMLDTHSVSAGLDYPGVGPEHAYLKETGRC
15606106_Aquifex_trpB1         HAASICGGSVGSYFLQDEEGQIQPTHSISAGLDYPGVGPEHALFHEIKRA
20808007_Thermoanaerobacter_te HASAMAKGSVGTYLLQDEEGKILPVHSISAGLDYPGVGPEHAYLKEIGRA
15673445_Lactococcus_lactis    HAATITKGRPGMDVLQDENGQMLEAFSISAGLDYPGIGPEHSYFNAVGRA
32417954_Neurospora_crassa     HSATLTAGSKGTYILQNQYGQIEDTHSISAGLDYPGVGPELSNWKDTERA
24215988_Leptospira_interr     HSATIQFGRTGTLVIQDDFGQIVPAHSVSAGLDYPGVGPEHAYFHKSGRV
2822116_Coprinopsis_cinerea    HSATLSMGQPGTYILQDKAGQIIETHSISAGLDYPGVGPEHAWLKDSGRA
15642912_Thermotoga_maritima   HAASLLKGKIGTFVLQDDWGQVQVTHSVSAGLDYSGVGPEHAYWRETGKV
29345943_Bacteroides           TAATIQLGKMGTYVIQNEDGQIEEPYSISAGLDYPGIGPIHANLAAQRRA
34557707_Wolinella_succin      HGASLAKGSPGSYLLQDSEGQIEEAYSISAGLDYPGIGPEHAYLFESGAA
15963781_Sinorhizobium_melilot HCASLTAGSPGTYLLQDGDGQIKEGHSISAGLDYPGIGPEHAWLNDIGRV
23502958_Brucella              HCASMSAGRPGTYLLQNADGQILEGHSVSAGLDYPGVGPEHSWLKDSGRV
22957700_Rhodobacter_sphaer    HCASLTGGRPGTYLLQDADGQILEGFSISAGLDYPGIGPEHAWLHDTGRA
464937_Pseudomonas_syringae    HAASLNGGVPGTFLLQDDDGQIIDAHSISAGLDYPGIGPEHAWLHDIGRV
23053570_Geobacter_b1          HAAPLCAGTVGTYLLQDEFGQIANAHSVSAGLDYPGVGPEHAWLKDSRRA
17546702_Ralstonia_solana      HAASLTGGSPGTYLLQDEDGQIIETHSISAGLDYPGVGPEHAWLKDAGRA
Leishmania_sanger_major        --------------------------------------------------
1174780_Zea_cp2                HAATLTKGQVGSYLLQDDDGQVIEPHSISAGLDYPGVGPEHSFLKDIGRA
1174778_Zea_cp1                HAATLTKGQVGSYLLQDDDGQVIEPHSISAGLDYPGVGPEHSFLKDIGRA
7437014_Oryza_sativa           HAATLTKGDVGSYLLQDDDGQVIEPHSISAGLDYPGVGPEHSFVKDMGRA
15236977_Arabidopsis_b2        HAATLTKGDVGSYLLQDDDGQIIEPHSISAGLDYPGVGPEHSFLKDVGRA
15239755_Arabidopsis_b1        HAATLTKGDVGSYLLQDDDGQIIEPHSISAGLDYPGVGPEHSFFKDMGRA
3915165_Camptotheca_cp_b2      HAATLTKGEVGSYLLQDDDGQIIEPHSISAGLDYPGVGPEHSFLKDIGRA
17231286_Nostoc                HAATLTKGRVGSYLLQDEDGQVIEAHSISAGLDYPGVGPEHSYLKDVGRA
17227906_Nostoc_sp             HAATLTLGRVGSYLLQDEDGQVIEAHSISAGLDYPGVGPEHSYLKDIRRA
33239640_Prochlorococcus       HAATITEGRIGSLLLQDSNGQVQEAHSISAGLDYPGVGPEHSYLKEIGRA
7437011_Chlamydomonas          HAATLTMGTPGSYLLQDDDGQIIDPHSISAGLDYPGIGPEHSFLKDVKRA
4325276_Gracilaria_cp          --------------------------------------------------
136282_Thermus_thermo          HAASIGAGKRGMYLLYDHDGQITPAHSVSAGLDYPGVGPEHSYYADAGVA
15805965_Deinococcus_radio     HAASVAWGRVGMYLMNDDEGQIVPPHSISAGLDYPGIGPEHCYYSVMGMA
29832719_Streptomyces_avermi   HAATLTAGEPGSYVLQDDEGQITEPYSISAGLDYPGIGPEHAYLKDSGRG
21673356_Chlorobium_tepid      HAASLTKGEIGMKLLQDEYGQVQEAHSISAGLDYPGVGPEHCYLQKLGLV
22974734_Chloroflexus_aurant   HAARFRAATPGSYVLQDEAGQIALTHSVSAGLDYASIGPEHAWLHDSGRA
28898734_Vibrio_paraha         HGAPLKHGKTGAPLMQDENGQVEESYSVSAGLDFPSVGPQHAHLNAIGRA
924784_E_coli                  HGAPLKHGRVGAPMMQTEDGQIEESYSISAGLDFPSVGPQHAYLNSTGRA
16273336_Haemophilus_influ     HGAPLKHGTTGSPIMQDKDGQIEESYSISAGLDFPSVGPQHAYLNEIGRA
15612264_Helicobacter_pylori   HGATLNKGRVGTYLLQDDEGQIAESHSISAGLDYPGVGPEHSYLKEIGRA
23473438_Desulfovibrio_desulf  NSAPINLGTPGTMLLQSADGQILPSHSVAAGLDYPGVGPEHAALGRSGRA
```

```
23473141_Desulfovibrio_desulf       HAATLCLGEPGSYMLKDQAGEPAAVYSISAGLDYPGVGPEHSHLKDLGRA
19703662_Fusobacterium_nuclea       HAATLTLGTVDTYALFNEDGSVKPVYSISPGLDYPGIGPEHAFLRDSKRA
29840324_Chlamydophila_caviae       HAARFATGRPGSYLLQDNEGQVLPTHSISAGLDYPAVGPDHAVLYESGRA
29840318_Chlamydophila_caviae       HAARFATGKPGSYVLQDEDGNCADTYSISAGLDYVSVGPTHAEMHESGRA
14520795_Pyrococcus_abyssi          YTYDYGDSAGLKLKMHTLGHRYYVPPIHAGGLRYHGLAPTLSVLINHGIV
14520795_Pyrococcus_b2              YTYDYGDSAGLKLKMHTLGHRYYVPPIHAGGLRYHGLAPTLSVLINHGIV
18313363_Pyrobaculum                YKYDFPDAVGVMLKMYTLGHDYVPPAIHAAGLRYHGAAPSLSLLRKLGIV
15606594_Aquifex_trpB2              YKYDFGDSVGLLIKMYTLGHDFVPSPIHAGGLRYHGDAPLVCKLYNLGYI
11498839_Archaeoglobus_b1           YKYDFGDVAGLLLKMYTLGHDFIPPPIHAGGLRYHGDAPTLCMLVKHGVI
15643305_Thermotoga_maritima        YDYDFGDTAGLLLKMYTLGKDFIPPKIHAGGLRYHGSAPIIARLVKEGLV
23053561_Geobacter_b2               YAFDYGDTAKMIAMMYTLGHDFMPPGIHAGGLRYHGESALVSQLHHAGLI
21673033_Chlorobium_tepid           YAYDSGDVAMMLLPMHSLGHTFIPPAIHAGGLRYHGMAPLVSHTKQLGLI
29350072_Bacteroides                FQYDFGDEAGYLLPMFTLGHNFAPAHIHAGGLRYHGAGVIVSQLLKDNLM


34557127_Wolinella_succin           HSVGVSDEEAIKNFFALSRLEGIIPAIESAHAIAYGMKLAKERLGKILIN
222_2_Cryptosporidium_parvum        KTVCITDQEAINGFFELSRTEGIIPAIESSHAIGYVLKIAKEMKGKILIN
22958844_Rhodobacter_sphaer         TYTGADDREALAAFFALSRHEGIIPALESAHAVAFAMREAKAHPGSILVN
28900440_Vibrio_paraha              NYGSINDDEAIDAFFELSREEGIIPAIESSHAVAYAIKLAQQGESSILVN
33456993_Cycloclasticus_sp          KYVAIDDQECIDAFMTLSRVEGIIPALESAHAVAYAMKIAKKMSSTILVN
15614226_Bacillus_halod             KYDAVTDKEALDALKQLSVHEGIIPAVESAHALAKAFERAKQLSQSILVC
16079321_Bacillus_subti             TYDSITDEEAVDALKLLSEKEGILPAIESAHALAKAFKLAKGMDRLILVC
14520675_Pyrococcus_abyssi          EYVTVTDEEALRAFHELSRTEGIIPALESAHAVAYAIKLAREMSRVIIVN
11499193_Archaeoglobus_b2           EYVTVNDEEALRAFKTLSKLEGIIPALESAHAIAYAMKMAEEMQRVLVVN
15606106_Aquifex_trpB1              KYTTATDEEALEGFKLLARTEGIIPALESAHAVIKAVEVARELGKIVVIN
20808007_Thermoanaerobacter_te      EYVYATDEEALAAFMDLSRTEGIIPALESAHALAYAMKLAKKLDEIIVVN
15673445_Lactococcus_lactis         KYVDITDEEALEGFKILSRTEGIIPALESSHAIAYAVKLAKELGASMIVC
32417954_Neurospora_crassa          KFVAATDAQAFEGFRLMSQLEGIIPALESSHGIWGALELAKTMKPDVVIC
24215988_Leptospira_interr          EYVNVDDEGALDAFIEVCRIEGIIPALETAHAFRYAKDLARTMSKNILIC
2822116_Coprinopsis_cinerea         DYVVCTDEDALRGFRMLTQKEGIIPALESSHAIWEGVKIAKSLPKDIVIC
15642912_Thermotoga_maritima        LYDAVTDEEALDAFIELSRLEGIIPALESSHALAYLKKINIKG--VVVVN
29345943_Bacteroides                TVLAVNDDEAIEAAYELTKLEGIIPALESAHALGALKKLKFKPE-VVVLT
34557707_Wolinella_succin           EYDHVTDAEALEAFVWLSQKEGIIPAFESAHAIAYLKKARERFKDVVIVS
15963781_Sinorhizobium_melilot      EYVPIMDHEALEAFQTLTRLEGIIPALEPSHALAEVIKRAPKMGKIILMN
23502958_Brucella                   DYVPILDNEALDAFQLCTRTEGIIPALESAHAIAQAVKMAPTMGKVMIVN
22957700_Rhodobacter_sphaer         EYVSITDAEALEAFQLCCALEGIIPALEPSHALAHVIKIAPTLPRIIVMN
464937_Pseudomonas_syringae         QYTSVTDDEALDAFHKCCRLEGIIPALESAHALAEVFKRAPTLPKLMVVN
23053570_Geobacter_b1               EYVSVTDDEALEAFTLLTREEGIMPALESSHAIAHVVKLAPTLPRTIVAC
17546702_Ralstonia_solana           QYVGITDKEALQAFHDLCRMEGIIPALESSHALAYACKLAPTLPKILLVN
Leishmania_sanger_major             --------------------------------------------------
1174780_Zea_cp2                     EYDSVTDQEALDAFKRVSRLEGIIPALETSHALAYLEKLCPTLADRVVVN
1174778_Zea_cp1                     EYDSVTDQEALDAFKRVSRLEGIIPALETSHALAYLEKLCPTLPDRVVLN
7437014_Oryza_sativa                EYDSATDEEALAGFKRVSRLEGIIPALETSHALAYLEKLCPTLFDRVVFN
15236977_Arabidopsis_b2             EYFSVTDEEALEAFKRVSRLEGIIPALETSHALAHLEKLCPTLPDRVVLN
15239755_Arabidopsis_b1             EYYSITDEEALEAFKRVSRLEGIIPALETSHALAYLEKLCPTLSDRVVLN
3915165_Camptotheca_cp_b2           EYYCCTDEEALEAFKRLSRLEGIIPALETSHALAFLEKLCPTLPNKVVLN
17231286_Nostoc                     EYYSVTDEEALAAFQRLSRLEGIIPALETAHAIAYLETLCPQLDGRIVIN
17227906_Nostoc_sp                  EYYSVTDKQALDGFQQLSRLEGIIPALETSHAIAYLETLCPQLNGRIVIN
33239640_Prochlorococcus            EYAAVTDQEALSALQLLSEKEGIIPALETAHALAFLEKLCPTLSSEIVIN
7437011_Chlamydomonas               EYYAVTDAEALEGFQLLSKLEGIIPALETSHAIAYLEKLIPTLKSRVVIN
4325276_Gracilaria_cp               --------------------------------------------------
136282_Thermus_thermo               EYASVTDEEALEGFKLLARLEGIIPALESAHAIAYAAKVVPEMDKVVVIN
15805965_Deinococcus_radio          EYVPVTDAQALEGLQLLTRLEGIIPALESAHAISYAVQLARQMKPIVVVN
29832719_Streptomyces_avermi        EYRAVTDDAAMQALRLLSRTEGIIPAIESAHALAGALEIGKELGKLILVN
21673356_Chlorobium_tepid           CYTSTTDKEALAALDALAKTEGIICALESAHAVHYAMKRAAEMPKIIVVN
22974734_Chloroflexus_aurant        TYTAASDDEALAAFQLLARLEGIIPALESAHAVAEAIRLAPTMRPIILVN
28898734_Vibrio_paraha              EYDNVTDDEALEAFQELARSEGIIPALESSHALAHALRMARENPELLVVN
924784_E_coli                       DYVSITDDEALEAFKTLCLHEGIIPALESSHALAHALKMMRENPDLLVVN
16273336_Haemophilus_influ          DYVSITDEEALNAFQELAKHEGIIPALESSHALAYALKLIKQNPELLVVN
15612264_Helicobacter_pylori        VYESASDIEALEAFRLLCQKEGIIPALESSHALAYALKLAQKCAQIIVVN
23473438_Desulfovibrio_desulf       RYGMVNDAQALHAFKTLSRMEGIIPALESSHAVAWVLENRDGIPRDVVVN
23473141_Desulfovibrio_desulf       EYVNASDAEALSAFFDLSRTEGIIPALESAHALAYAMRLAPTLSSIIAVN
19703662_Fusobacterium_nuclea       EYVSATDDEAVNALLLLTKKEGIIPAIESSHALAEVIKRAPKLNKIIIVN
29840324_Chlamydophila_caviae       SYTVATDKAALEAFFLLSRLEGIIPALESSHALAELINIAPTLPKVVVVN
29840318_Chlamydophila_caviae       QYTYATDDEALEAFRLLSKTEGIIPALESAHALAHMIKIAPSLDKIAIVN
14520795_Pyrococcus_abyssi          KPIAYHQTEVFEAAVLFAKAEGIVPAPESAHAVKAVIDKALEARREMVIL
14520795_Pyrococcus_b2              KPIAYHQTEVFEAAVLFAKAEGIVPAPESAHAVKAVIDKALEARREMVIL
18313363_Pyrobaculum                EAVAYPQEEVMRAALLFARTEGIVPAPESAHAIKAAVDLAKKLPR--VIA
15606594_Aquifex_trpB2              DAVAYKQTEVFEAAVTFARTEGIVPAPESAHAIKAAIDEALKCKEEKVIL
11498839_Archaeoglobus_b1           KARAVKQLPTFEAGLLFARTEGIIPAPETNHAVRAAIDEAIKAREEKVIV
15643305_Thermotoga_maritima        EAQAFDQDETFEAAKIFAKLEGIIPAPESAHAIAGAIREAKKAKEERVIV
23053561_Geobacter_b2               EAKSYRQNACFEAAHLFARSEGIVPAPESSHAVRAAIDEAVLAKEEKTIL
21673033_Chlorobium_tepid           EATALPQTECYEAALLFAHTEGFIPAPETSHAIAQTIREAKQAKEEKVIL
```

```
29350072_Bacteroides                EAVDIQQLESFEAGCLFAQAEGIIPAPESSHAIAAAIREAKACKEEKVIL


34557127_Wolinella_succin           LSGRGDKDID
222_2_Cryptosporidium_parvum        LSGRGDKDLD
22958844_Rhodobacter_sphaer         LSGRGDKDID
28900440_Vibrio_paraha              LSGRGDKDID
33456993_Cycloclasticus_sp          LSGRGDKDAD
15614226_Bacillus_halod             LSGRGDKDVH
16079321_Bacillus_subti             LSGRGDKDVN
14520675_Pyrococcus_abyssi          LSGRGDKDLD
11499193_Archaeoglobus_b2           LSGRGDKDMD
15606106_Aquifex_trpB1              LSGRGDKDMA
20808007_Thermoanaerobacter_te      LSGRGDKDVN
15673445_Lactococcus_lactis         LSGRGDKDVV
32417954_Neurospora_crassa          LSGRGDKDVQ
24215988_Leptospira_interr          LSGRGDKDVA
2822116_Coprinopsis_cinerea         LSGRGDKDVE
15642912_Thermotoga_maritima        LSGRGDKDLE
29345943_Bacteroides                VSGRGDKDIE
34557707_Wolinella_succin           LSGRGDKDMI
15963781_Sinorhizobium_melilot      LSGRGDKDIF
23502958_Brucella                   LSGRGDKDVH
22957700_Rhodobacter_sphaer         MCGRGDKDIF
464937_Pseudomonas_syringae         LSGRGDKDMQ
23053570_Geobacter_b1               LSGRGDKDIH
17546702_Ralstonia_solana           LSGRGDKDMH
Leishmania_sanger_major             ----------
1174780_Zea_cp2                     CSGRGDKDVH
1174778_Zea_cp1                     CSGRGDKDVH
7437014_Oryza_sativa                FSGRGDKDVD
15236977_Arabidopsis_b2             FSGRGDKDVQ
15239755_Arabidopsis_b1             FSGRGDKDVQ
3915165_Camptotheca_cp_b2           CSGRGDKDVH
17231286_Nostoc                     CSGRGDKDVQ
17227906_Nostoc_sp                  CSGRGDKDVQ
33239640_Prochlorococcus            CSGRGDKDVN
7437011_Chlamydomonas               CSGRGDKDVN
4325276_Gracilaria_cp               ----------
136282_Thermus_thermo               LSGRGDKDVT
15805965_Deinococcus_radio          LSGRGDKDVT
29832719_Streptomyces_avermi        LSGRGDKDMD
21673356_Chlorobium_tepid           LSGRGDKDMG
22974734_Chloroflexus_aurant        LSGRVTKNIL
28898734_Vibrio_paraha              LSGRGDKDIF
924784_E_coli                       LSGRGDKDIF
16273336_Haemophilus_influ          LSGRGDKDIF
15612264_Helicobacter_pylori        LSGRGDKDLS
23473438_Desulfovibrio_desulf       LSGRGDKDMG
23473141_Desulfovibrio_desulf       LSGRGDKDVA
19703662_Fusobacterium_nuclea       ISGRGDKDVA
29840324_Chlamydophila_caviae       LSGRGDKDLE
29840318_Chlamydophila_caviae       LSGRGDKDLS
14520795_Pyrococcus_abyssi          FNLSGHGLLD
14520795_Pyrococcus_b2              FNLSGHGLLD
18313363_Pyrobaculum                FNLSGHGLLD
15606594_Aquifex_trpB2              FNLSGHGYFD
11498839_Archaeoglobus_b1           FGFSGHGLLD
15643305_Thermotoga_maritima        FTLSGHGLLD
23053561_Geobacter_b2               FCLSGHGQLD
21673033_Chlorobium_tepid           MNWSGHGLMD
29350072_Bacteroides                FNLSGHGLID
```

9) 1,4-alpha-glucan branching enzyme (Figure 5)

CLUSTAL X (1.81) multiple sequence alignment

The *Toxoplasmsa gondii* sequences were obtained from http://ToxoDB.org release 3.0.

```
45548447_Rubrobacter_xylano         MAIYEVHLGSWRRRTYRELAEELVPYVAGLGYTHVELLPPMEHPFGGSWG
45916434_Mesorhizobium_sp.          ISIYEVHLGSWRRHSYDELADRLLPYVKDLGFTHIECMPVSEHPFSGSWG
22968855_Rhodospirillum_rubrum      VSIYEVHLGSWRHGSYREMAEQLIPYVKEMGFTHIELLPVSEFPFDGSWG
22986676_Burkholderia_fungor        ISIYEVHAESWLRVDWEELAERMIPYVKSMGFTHVEFMPIAEHPFGGSWG
23468472_Pseudomonas_syring         LSIYELHAGSWQMENWRELADRLIPYVKELGFTHIELMPIMEHPFGGSWG
29829347_Streptomyces_avermi        FSVYEVHLPSWRPGTYRQLAEQLPAYVADLGFTHVELLPVAEHPFGGSWG
15806848_Deinococcus_radiod         ISIYECHVGSWARRNYRDLAHRLGEYVTYMGYTHVELLGVMEHPFDGSWG
22999765_Magnetococcus_sp.          ISIYEVHLGSWLDANYRELGKRLARYCSEMGFTHVELMPINEHPFDGSWG
23474508_Desulfovibrio_desulf       VSIYELHAGSWMRRNWDELAGTLIPYVRDAGFTHIELMPVAEHPLDQSWG
32472075_Pirellula_sp.              MNVYEVHLGSWQKGDYRDLAKRLVDYCHRMNFTHVELMPISEHPFTGSWG
22971471_Chloroflexus_aurant        ISIYEVHLGSWRRKNYRELAHELVEHVKDCGFTHVELLPVTEHPFYGSWG
15606119_Aquifex_aeolic             ISIYEVHVGSWRRVSYRELAEYLPYYVKEMGFTHVEFLPVMEHPFYGSWG
23021493_Clostridium_thermo         ISIYEVHLGSWKRVSYRELADMLVEYVKYMGYTHIELLPIAEHPFDGSWG
17228208_Nostoc_sp.                 ISVYEVHLGSWLHATYRELASRLIPYVKELGYTHIELLPIAEHPFDGSWG
45509261_Anabaena_variab            ISVYEVHLGSWLHATYRELASRLIPYVKELGYTHIELLPIAEHPFDGSWG
23126496_Nostoc_punctif             VSVYEVHLGSWLHATYRELADRLIPYVKELGYTHIEMLPIAEHPFDGSWG
15618386_Chlamydophila_pneumo       VTIYEVHLGSWQWQSYSEMAHRLASYCKEMHYTHVELLPITEHPLNESWG
18309045_Clostridium_perfri         INIYELHLGSWKRKSYDELSIILPKYIKEMGYTHVEFMPLNEHPLDASWG
16080150_Bacillus_subtil            VFIYELHLGSWKKHSYKELSQTLIPYIKKHGFTHIELLPVYEHPYDRSWG
19704191_Fusobacterium_nuclea       INIYEVHLGSWKKKNYKEIAELLVEYMLEMNYTHIEIMPIIEYPFDGSWG
32398905_Cryptosporidium_parvu      MSIYEVHLPSWMRRGYREIAERLVEHVKNLNFTHVEFLPLAQHPFEGSWG
T_gondii_TgTwinscan_5121            MAIYEVHLPSWMRGSYRELADRLVRHVKTMNFTHVEFLPLAHHPFEGSWG
23135626_Cytophaga_hutchi           PIIYEAHVGMAQEKTYREFADKIVPRIRQMGYNSIQLMAVMEHPYYGSFG
28829250_Dictyostelium_discoi       LRIYEAHVGMSSELTYSKFKDTVLPMVKELGYNCIQLMAVMEHAYYASFG
17554896_Caenorhabditis_elegan      LRIYEAHVGISSSETYREFADDVLPRIQKQGYNAIQLMAVMEHVYYASFG
32564391_Caenorhabditis_elegan      LRIYEAHVGISSSETYREFADDVLPRIQKQGYNAIQLMAVMEHVYYASFG
16416077_Neurospora_crassa          LRIYEAHVGISSPETYKEFTKTMLPRIKNLGYNAIQLMAIMEHAYYASFG
171569_Saccharomyces_cerev          LRIYEAHVGISSPETYKEFTEKVLPRIKYLGYDAIQLMAIMEHAYYASFG
28573410_Drosophila_melano          LRIYECHVGIASQESYDEFADRIVPRIKRQGYNCIQVMAIMEHAYYASFG
4557619_Homo_sapien                 LRIYESHVGISSHESYKHFTCNVLPRIKGLGYNCIQLMAIMEHAYYASFG
30680140_Arabidopsis_thalia         LRIYEAHVGMSSTETYANFRDDVLPRIKKLGYNAVQIMAIQEHSYYASFG
15227650_Arabidopsis_thalia         LRIYESHVGMSSTETYANFRDDVLPRIKKLGYNAVQIMAIQEHAYYASFG
1169912_Solanum_tuber               PRIYEAHVGMSSSESYREFADDVLPRIKANNYNTVQLMAIMEHSYYGSFG
2801805_Gracilaria_gracil           LRIYECHVGMGSNDSYREFADNVLPRIKETGYTALQIMAIMEHAYYASFG
29346181_Bacteroides_thetai         LLIYECHIGMAQQETYNEFREKTLPRIAQEGYNCIQIMAIQEHPYYGSFG
29245217_Giardia_lambli             HRIYECHVGMSSTETYREFADILLPIIKEKGYNVIQLMAIQEHSYYGSFG
32470978_Pirellula_sp.              LVIYEMHIGTFHRDGTFSDAVAKFKHLKELGVNAIQIMPVAEFAGDLSWG
45916029_Mesorhizobium_sp.          LVIYEMHIGTFHRPGTFDDATERLAHLKRLGINAVQIMPAMEFAGDISWG
45548449_Rubrobacter_xylano         AVVYELHVGTFSPETFRGACER-LDHLAGLGVTAVELMPLADFPGSRNWG
22968918_Rhodospirillum_rubrum      TVLYELHLGTFSRKTYEGARAR-LGDLVDLGVSAIELMPLADFVGQRNWG
22986678_Burkholderia_fungor        TVLYELHVGALGG--YAGVQKR-LPELAALGVTAIELMPLNDFPGKHNWG
23471845_Pseudomonas_syring         AVIYELHVGAMGG--YAGVEKH-LPRLARLGVTAIELMPLAQFPGDRNWG
22971470_Chloroflexus_aurant        LVIYELHVGTFTPETFEAIIPH-LPLLRDLGITAIELMPVAHFPGVRNWG
23124761_Nostoc_punctif             MIFYELHVGTFTPETFTAIISR-LPELRELGINAIEIMPIAQFPGDRNWG
23475793_Desulfovibrio_desulf       AVIYEIHTGTFTPETLDAAAGR-LPRIAELGVTAVELMPLTETTGRWNWG
22968087_Rhodospirillum_rubrum      MVLYEMHIGTFTPETWAAAMAH-LERLRDLGVTCLEIMPIAEFSGRFGWG
46135186_Anabaena_variab            LVIYELHVGDFSGGKYKHVIEK-LDYLCELGINAIELLPVKEYPGDYSWG
23130645_Nostoc_punctif             LVIYEMHVADFTGDKYLGIIAK-LDYLCELGINAIELMPVNEYPGDYSWG
32398951_Cryptosporidium_parvu      LIIYELHVESYIRRFFRQIADYGLDNIKDLNFNCLELMPIVEYCG--EWG
T_gondii_TgTwinscan4106             LIIYELHPGTFS--VFETLVDK-LDHIRSLNFNAVEFLPVQEFGG--EWG
23123689_Nostoc_punctif             LVIYELHVGALGYGGNFEDAIALLPYLVQLGVNAVELLPMSEFRDEVNWG
23050680_Methanosarcina_barker      IVLYELMVNEFADD-----TIDHLDYLADLGINCIELMPLSNIEKRVDWG
                                      .**                             :: :   .      :*

45548447_Rubrobacter_xylano         YQPVGLFAPTSRFGPPEDFKHLVDAFHRAGVGVIADWVPAHFPEDRKGRH
45916434_Mesorhizobium_sp.          YQPVGLYAPTSRFGTPEDFARFVDRCHHEGIGVIIDWVPAHFPSDRLGFH
22968855_Rhodospirillum_rubrum      YQPIGLFAPTSRFGEPDDFRHFVDRCHQEGVGVILDWVAGHFPEDRQGRH
22986676_Burkholderia_fungor        YQPLGQFAPSARFGKPEQFARFVDKAHEAGLGVILDWVPAHFPNDREGYH
23468472_Pseudomonas_syring         YQLLAQFAPTARYGSPEDFAFFVDACHRAEIGVILDWVPAHFPTDKEGFH
29829347_Streptomyces_avermi        YQVTGFYAPTARLGTPDDFKYLVDALHRAGVGVLMDWVPAHFPRDLRAAH
15806848_Deinococcus_radiod         YQVTGYYAPTSRLGSPEDFKYLVNHLHSLGIGVLLDWVPGHFPTDRKGYH
22999765_Magnetococcus_sp.          YQSTGYFAVTSRFGDGDDFRAMVDQLHGANIGVILDWVPAHFPKDRLGEH
23474508_Desulfovibrio_desulf       YQTGAYYAPTSRFGTPDDLRRFVDLCHQQGIGVILDWVPAHFPKDRLGEH
32472075_Pirellula_sp.              YQSVGYYAPTSRHGSPEDFMFFVDHMHQNGIGVLIDWVPAHFPKDRQGEH
22971471_Chloroflexus_aurant        YQSTGMFAPTSRYGTPQDFMYFVDYLHQNGIGVILDWVPSHFPTDRKGYH
```

```
15606119_Aquifex_aeolic              YQITGYFAPTSRYGTPQDFMYLIDKLHQEGIGVILDWVPSHFPTDRKRWH
23021493_Clostridium_thermo          YQVTGYYAATSRYGQPKDFMYFVDKCHQNGIGVIIDWVPAHFPKDKQGEH
17228208_Nostoc_sp.                  YQVTGYYAPTSRFGTPEDFMYFVDQCHQNNIGVLVDWVPGHFPKDRKGEH
45509261_Anabaena_variab             YQVTGYYAPTSRFGTPEDFMYFVDQCHQNNIGVIVDWVPGHFPKDRKGEH
23126496_Nostoc_punctif              YQVTGYYAPTSRFGSPEDFMYFVDKCHQNDIGVIVDWVPGHFPKDRKGEH
15618386_Chlamydophila_pneumo        YQVTGYYAPTSRYGTLQEFQYFVDYLHKENIGIILDWVPGHFPVDSQALH
18309045_Clostridium_perfri          YQVTGYYSITSRYGDIKGLKRLINALHKEDIGVILDWVPGHFCKDWKADN
16080150_Bacillus_subtil             YQGTGYYSPTSRFGPPHDLMKFVDECHQQNIGVILDWVPGHFCKDRDREN
19704191_Fusobacterium_nuclea        YQGTGYYSVTSRYGTPDDFMYFVNYFHKNNLGVILDWVPGHFCKDSLGEN
32398905_Cryptosporidium_parvu       YQVTGQYAPYSRLGTPDDFKYLVNELHKAKIGVFIDFVPAHFCKDREGEH
T_gondii_TgTwinscan_5121             YQVTGLYAPYSRLGSPDDFKYLVDKLHQSGIGVFLDFVPAHFCKDREGEH
23135626_Cytophaga_hutchi            YHVSNFFAPSSRFGTPEDLKYLVDRAHKFGLAVIMDVVHSHAVKNDRGYH
28829250_Dictyostelium_discoi        YQVTNFFAISSRFGTPEELKEMIDKAHEMGLLVFLDVVHSHASKNGRGNH
17554896_Caenorhabditis_elegan       YQVSNFFAVSSRCGTPEDLKYLVDKAHSLGIFMLLDVVHSHASKNARGYH
32564391_Caenorhabditis_elegan       YQVSNFFAVSSRCGTPEDLKYLVDKAHSLGIFMLLDVVHSHASKNARGYH
16416077_Neurospora_crassa           YQVNSFFAASSRYGPPEDLKELVDTAHAMGIAVLLDVVHSHASKNGRGKH
171569_Saccharomyces_cerev           YQVTNFFAASSRFGTPEELKELIDTAHSMGILVLLDVVHSHASKNGRGEH
28573410_Drosophila_melano           YQVTSFYAASSRYGNPEQLKRMIDVAHSHGLFVLLDVVHSHASKNARGEH
4557619_Homo_sapien                  YQITSFFAASSRYGTPEELQELVDTAHSMGIIVLLDVVHSHASKNPRGTH
30680140_Arabidopsis_thalia          YHVTNFFAPSSRCGTPEELKSLIDRAHELGLVVLMDIVHSHASKNPRGYH
15227650_Arabidopsis_thalia          YHVTNFFAPSSRFGTPDDLKSLIDKAHELGLVVLMDIVHSHASKNSRGYH
1169912_Solanum_tuber                YHVTNFFAVSSRYGNPEDLKYLIDKAHSLGLQVLVDVVHSHASNNERGYH
2801805_Gracilaria_gracil            YHVTNFFAISSRCGIPEDLKYLIDKAHQLGLYVFMDVVHSHASSNERGRH
29346181_Bacteroides_thetai          YHVSSFFAASSRFGTPDELKALIDAAHEMGIAVIMDIVHSHAVKNPRREH
29245217_Giardia_lambli              YQVTSFFAPSSRFGTPDDLKYLVDKAHEAGIAVLLDLVHSHASKN-KEDH
32470978_Pirellula_sp.               YNPAHIFAVEQAYGGPDALKAFVDAAHEAGFAVIIDVVYNHFGPSDHRSN
45916029_Mesorhizobium_sp.           YNPAHIFAIESSYGGPAAFKAFINAAHKQGIAVILDVVYNHFGPSDWRSD
45548449_Rubrobacter_xylano          YDGVLPYAPDSSYGTPEELKELVQAAHGRGIMVLLDVVYNHFGPETERHA
22968918_Rhodospirillum_rubrum       YDGVLPFAPDSVYGTPEQLKALIDAAHGLGLMVFLDVVYNHFGPETQAHQ
22986678_Burkholderia_fungor         YDGVLPYAPDSAYGRPEDLKALIDAAHGLGLMVFLDVVYNHFGPDREGTH
23471845_Pseudomonas_syring          YDGVLHYAPQSSYGTPEQLKHLIDTAHGLGLMVILDVVYNHFGPDRSDLH
22971470_Chloroflexus_aurant         YDGVYLYAPHTAYGGVTGLKRLVDAAHAHGIAVLLDVVYNHFGPETNRYQ
23124761_Nostoc_punctif              YDGVYPYAVQNSYGSPAELKQLVNACHQNNIAVVLDVVYNHFGPETKTYK
23475793_Desulfovibrio_desulf        YDGVFPYSIHHRAGGPDALRRFVDTAHGLGLSVYGDVVLNHLGPESRRHH
22968087_Rhodospirillum_rubrum       YDGVNLFAPTRLYGQPDDMRRFVDRAHALGLAVILDVVYNHLGPDTDRYK
46135186_Anabaena_variab             YNPRYFFATESSYGSTADLKKLVDECHQRGIRIIMDGIYNHSEASPRDPD
23130645_Nostoc_punctif              YKVRHFFATESSYGSTEDLKRLIDECHGRGIRVFMDGIYNHTDEEPEDPA
32398951_Cryptosporidium_parvu       YNPRLLLSINSHLGSVDDFIYLVQKVHEKDLFIVVDLVLHHGASKEGGGD
T_gondii_TgTwinscan4106               YNPRLLLATHGKYGTPDQLRKLVDECHRKGLAVIFDLVLNHGSAKEGGGE
23123689_Nostoc_punctif              YETSHYFALEYSAGGRDQLKHFIRECHCYGIAVILDVVYNHFNPDSGDYF
23050680_Methanosarcina_barker       FAPIGYFGVDERFGNRKDMQELIDKAHQKDIAVIVDSVYAHTSHLGPFAE
                                     :      .     *    :  ::   *   . :  * :  *

45548447_Rubrobacter_xylano          PDWGTLIYNYGRNEVRNFLISNALFWLDEYHIDGLRVDAVASMLYAFTVA
45916434_Mesorhizobium_sp.           RDWNTLIYNFGRREVANFLTANALFWLDRYHVDGLRVDAVASMLYTTSIA
22968855_Rhodospirillum_rubrum       MDWGTYIFNYGRNEVRNFLLANALFWLEQFHIDGLRVDAVASMLYAVTIA
22986676_Burkholderia_fungor         QDWNTMIYNLGRNEVSAFLIASGLAWLKRYHVDGLRVDAVASMLYAITIA
23468472_Pseudomonas_syring          QDWNTLIYNLGRTEVHGFMLASALHWLKHYHIDGLRVDAVASMLYTMVIA
29829347_Streptomyces_avermi         PDWGTLEFDYGRREVRNFLVANAVYWCEEFHIDGLRVDAVASMLYVVTIA
15806848_Deinococcus_radiod          YDWNTYIFDYGRNEVVMFLIGSALKWVQDYHVDGLRVDAVASMLYCLMIA
22999765_Magnetococcus_sp.           QDWDTLIFNFGRLEVKSFLISSALHMLTRYHLDGLRVDAVASMLYVLMIA
23474508_Desulfovibrio_desulf        PDWGTYVFNYGRHEVRNFLFANALYWFKEFHVDGLRIDAVASMLYVMMIA
32472075_Pirellula_sp.               PDWGTMIFNFGRNEVKNFLIANALFWLDKYHIDGLRVDAVASMLYVITAA
22971471_Chloroflexus_aurant         PDWGSYIYNYGRNEVRSFLISSALCWLDKFHIDGIRVDAVASMLYVQTIA
15606119_Aquifex_aeolic              PDWNSFVFDYGKPEVRSFLLSSAHFWLDKYHADGLRVDAVASMLYVQTIA
23021493_Clostridium_thermo          PDWGTHIFNYGRNEVKNFLIANAMFWFDKYHIDGLRVDAVASMLYVMMIA
17228208_Nostoc_sp.                  KEWGTLVFNYSRNEVRNFLVANALFWFDKYHIDGIRVDAVASMLYVLSIA
45509261_Anabaena_variab             KEWGTLVFNYSRNEVRNFLVANALFWFDKYHIDGIRVDAVASMLYVLSIA
23126496_Nostoc_punctif              KGWGTLVFNYGRHEVSNFLAANALFWFDKYHIDGIRVDAVASMLYILSIA
15618386_Chlamydophila_pneumo        PHWNTFTFDYSRHEVTNFLLGSALFWLDKMHIDGLRVDAVASMLYVLTFA
18309045_Clostridium_perfri          KGWGTFNFDLGKPEVKSFLISNAFYFINEFHIDGLRVDAVSNMLYVITIA
16080150_Bacillus_subtil             WLWGTANFDLGKPEVHSFLISNALYWAEFYHIDGFRVDAVANILYVMMIA
19704191_Fusobacterium_nuclea        E-WGSANFNVSRNEVRSFLLSNLYFWIKEFHIDGIRMDAVSNMLYVMLIA
32398905_Cryptosporidium_parvu       KQWGTAVFNFRRNEVRSFLLGAAYHWLRRYHIDGLRIDAVSSMLYVFTMA
T_gondii_TgTwinscan_5121             KMWGTLVFNFRRSEVRSFLLGAAYHWLRRYHIDGLRIDAVSSMLYVFTMA
```

```
23135626_Cytophaga_hutchi           TGWDSKLFDYGKLEVKRFLLSNLRYWLEEYHFDGFRFDGVTSMLYSITIA
28829250_Dictyostelium_discoi       ELWDSRLFNYGNWEVMRFLLSNLRFYVDEYHFDGFRFDGVTSMIYIVTIA
17554896_Caenorhabditis_elegan      NLWDSRLFDYTQTETLRFLLSNVRWWVEEYGFDGFRFDGVSSMIYMITIA
32564391_Caenorhabditis_elegan      NLWDSRLFDYTQTE-LRFLLSNVRWWVEEYGFDGFRFDGVSSMIYMITIA
16416077_Neurospora_crassa          DLWDSRLFNYGHHEVMRFLLSNLRFWMDEYAFDGFRFDGVTSMLYVITVA
171569_Saccharomyces_cerev          PLWDSRLFNYGKFEVQRFLLANLAFYVDVYQFDGFRFDGVTSMLYAVTVA
28573410_Drosophila_melano          SLWDSRLFNYVEYEVLRFLLSNLRWWHDEYNFDGYRFDGVTSMLYIITIA
4557619_Homo_sapien                 DLWDSRLFAYSSWEVLRFLLSNIRWWLEEYRFDGFRFDGVTSMLYSITIA
30680140_Arabidopsis_thalia         WMWDSRLFNYGSWEVLRYLLSNARWWLEEYKFDGFRFDGVTSMMYAITVG
15227650_Arabidopsis_thalia         WMWDSRLFNYGSWEVLRYLLSNARWWLEEYKFDGFRFDGVTSMMYAIVVG
1169912_Solanum_tuber               KLWDSRLFNYANWEVLRFLLSNLRWWLEEYNFDGFRFDGITSMLYATVIA
2801805_Gracilaria_gracil           SLWDSRLFNYGHWEVLRFLLSNLRWYMEEYHFDGFRFDGVTSMLYAVTIA
29346181_Bacteroides_thetai         PAWDSLCFDYGKNEVIHFLLSNCKYWLEEYKFDGFRFDGVTSMLYAISIA
29245217_Giardia_lambli             PLWDSKIFNYKNPETLRFLLQNVRWWLQEFKIDGFRFDGVMSLMYILTIA
32470978_Pirellula_sp.              TPWGDTRPDYGRGEVRQFIHDNAMMWMREYHADGLRYDMTAYIRTCYLIA
45916029_Mesorhizobium_sp.          TPWGDTRPVYGRGEVRQFIRDNALFWLEEYHLDGLRFDMTLYIRNRITIA
45548449_Rubrobacter_xylano         TPWGAAVNFDDAGSVREFFVHNALYWIEEYGFDGLRLDAVHAIRDVHLVL
22968918_Rhodospirillum_rubrum      TPWGAAIDFERP--VRDFFVANALQWLEEYRFDGLRFDAVHAILDVHLVL
22986678_Burkholderia_fungor        TPWGPAIDFERS--VSDFFTDNAVYWINEYRIDGLRFDAVHAIDNVHLVL
23471845_Pseudomonas_syring         TPWGDAIDFRRP--VRDFFVDNALMWLLEYRFDGLRMDAVHAIRDVWLNL
22971470_Chloroflexus_aurant        TPWGAAINYDGPESVRWLIIENALEWLREYHIDGLRLDATHAIFDAYLFA
23124761_Nostoc_punctif             TPWGEALNFDDAHSVRNYFIENALYWLREFHIDALRLDAIQAIYDRHLIA
23475793_Desulfovibrio_desulf       TPWGACPSFDGASAVRRYFLDCCLRLMAHFHMDGLRLDATPYVQDLHIMA
22968087_Rhodospirillum_rubrum      NDWGVAINFDGPGSVRAFYLANAGYWIEEFHLDGLRLDATQQIYDTFVVA
46135186_Anabaena_variab            NNWGPEFNYEHYDEARKFIGDTVRYWVGEYHLDGIRYDAARQIANFYNVA
23130645_Nostoc_punctif             NYWGPEFNYDFYDKAWEYIGDVVRFWIQEYHIDGIRFDAVRQLANFYNIA
32398951_Cryptosporidium_parvu      TGWGAKFNFHKK--VQDMLYEACRVLLGEYGVDGIRFDSVHNMPNRFLIA
T_gondii_TgTwinscan4106              SGWGRKFSFHKR--VRDMILAAALCFIEEYGADGLRLDSVHSMPWKILIA
23123689_Nostoc_punctif             YPDGRRFPEGGYLDVRKMFISSAVTLMEEFHVDGFRVDQTTSIHSAILIA
23050680_Methanosarcina_barker      NAFGISTDFTYKFT-RNFFLTVNNHWLDSYHIDGFRYDYVPGYWLLIQCA
                                       .                            *. * *

45548447_Rubrobacter_xylano         EESTAWPMVSRMGGLGFGYKWNMGWMHDTLQYMKEDPVHR
45916434_Mesorhizobium_sp.          EESTAFPGVSRAGGLGFGFKWNMGWMHDTLDYMQHEPIHR
22968855_Rhodospirillum_rubrum      EESTAWPMVSRLGGLGFGYKWNMGWMNDTLSYMSQDPIYR
22986676_Burkholderia_fungor        EESTAWPGVTADGGLGFQFKWNMGWMHDTLHYMEEDPVYR
23468472_Pseudomonas_syring         EESTAWPGVSEQGGLGFNYKWNMGWMHDSLQYMEEDPINR
29829347_Streptomyces_avermi        EESTAWEGVTRDNGLGFGLKWNMGWMHDSLGYVQHEPVHR
15806848_Deinococcus_radiod         EESTSFPGVTTEG-LGFDYKWAMGWMNDSLAYFEQDPIYR
22999765_Magnetococcus_sp.          EESTAWPQVTRLGGLGFNMKWNMGWMNDTLSYFSKDPIHR
23474508_Desulfovibrio_desulf       EESTSWPGVSRTGGLGFTFKWNMGWMHDTLNYMRQDPVFR
32472075_Pirellula_sp.              EESTAWPGVSRDGGLGFTYKWNMGWMNDTLSYMRNEPIHR
22971471_Chloroflexus_aurant        EESTAWPMVSRVGGLGFGFKWDMGWMHDTLQYFRRDPIYR
15606119_Aquifex_aeolic             EESTAWPMVSRVGGLGFGMKWNMGWMNDTLFYFSKDPIYR
23021493_Clostridium_thermo         EESTAWALVTKTGGLGFSYKWNMGWMNDFLRYMSMDSVYR
17228208_Nostoc_sp.                 EESTDWPMVSWTGGLGFNLKWNMGWMHDMLDYFSMDPWFR
45509261_Anabaena_variab            EESTDWPMVSWTGGLGFNLKWNMGWMHDMLDYFSMDPWFR
23126496_Nostoc_punctif             EESTSWPMVSWTGGLGFNLKWNMGWMHDMLDYFSMDPWFR
15618386_Chlamydophila_pneumo       EESTAFPGVTKQGGLGFDYKWNLGWMHDTFHYFMKDPMYR
18309045_Clostridium_perfri         EESTSWPNVTNYGGLGFDFKWNMGWMNDTLEYNELDPIYR
16080150_Bacillus_subtil            EDSTEWPQVTGEGGLGFHYKWNMGWMNDVLKYMETPPEER
19704191_Fusobacterium_nuclea       EDSSAWPLVTKDGGLGFDFKWNMGWMNDTLKYMEQDPFFR
32398905_Cryptosporidium_parvu      EESTAWQGVTH--GLGFDAKWDLGWMNDTLSYLYTPPDNK
T_gondii_TgTwinscan_5121            EESTSWQGVTDFKGLGFDAKWDLGWMNDTLSYLCLPLYKR
23135626_Cytophaga_hutchi           EDVSGMPGLSRDGGVGFDYRLAMGIPDFWIKYLKHLQDEQ
28829250_Dictyostelium_discoi       EEVTGLATLCREGGGDFDYRLAMGIPDKWIELVKEKKDED
17554896_Caenorhabditis_elegan      EEVSGMPGICREGGQGFDYRLAMALPDMWIKILKHTSDED
32564391_Caenorhabditis_elegan      EEVSGMPGICREGGQGFDYRLAMALPDMWIKILKHTSDED
16416077_Neurospora_crassa          EDVSGMPALCLLGGVGFDYRLAMAIPDMWIKILKEKKDEE
171569_Saccharomyces_cerev          EDVSGYPTLCLIGGTGFDYRLAMALPDMWIKLIKEKKDDE
28573410_Drosophila_melano          EDVSGMPTLCREGGIGFDYRLGMAIPDKWIELLKEQSDDE
4557619_Homo_sapien                 EDVSGMPALCSQGGGGFDYRLAMAIPDKWIQLLKEFKDED
30680140_Arabidopsis_thalia         EDVSGMPTFCIDGGVGFDYRLHMAIADKWIEMLK-KRDED
15227650_Arabidopsis_thalia         EDVSGMPAFCVDGGVGFDYRLHMAVADKWIELLKKRDEDW
1169912_Solanum_tuber               EDVSGMPGLGREGGIGFDYRLAMAIPDKWIDYLKNKNDED
```

```
2801805_Gracilaria_gracil          EDVSGMPTLCVRGGLGFDYRLAMAIPDMWIEVLEKEKDEN
29346181_Bacteroides_thetai        EEVSGMPGLAADGGYGFDYRMAMNIPDYWIKTIKEKIDED
29245217_Giardia_lambli            EDVSGYPCMATDGGIGFDYRFQMAVPDLWINMMKHGFDTG
32470978_Pirellula_sp.             EDLQTNNWLTKQGGAGFTTQWDAAFVHPIRSAVQEIDDAH
45916029_Mesorhizobium_sp.         EDLRNKAEITGEGGAGFGAQWDSEFVHPIRETLIAPDDES
45548449_Rubrobacter_xylano        ENEENEASLLREGRSLYDAQWNDDLHHALHVALTGEDAAY
22968918_Rhodospirillum_rubrum     ENDANEARRLIAVRGRYDAQWNDDIHHAFHVVATGETGGY
22986678_Burkholderia_fungor       ENEHNSANLLE---THFDAQWNDDAHNTLHVLLTGETEGY
23471845_Pseudomonas_syring        ENEFNQASLLE---KGFDAQWDDDGHNTLHVLLTGETDAY
22971470_Chloroflexus_aurant       EHPLNDPRFARLGGYGLSGVWSDDFHHALHTLLTGEQRGY
23124761_Nostoc_punctif            ESDLNNPQIIRLGGYGLDAQWSDDFHHSLHALLTGDRQGY
23475793_Desulfovibrio_desulf      ESTRNEIHLTTAGGYGMAAQWSDDFHHAVHARLTGESCGY
22968087_Rhodospirillum_rubrum     ENEPQEVRLIEAQGYGLDALWNDDLHHAAMVALTGRREAY
46135186_Anabaena_variab           EHIPETTSITN-LDGPMDGCWHDSFYHTIKAHICGDT---
23130645_Nostoc_punctif            EHIPDTNTVVK-PDGPLDACWHESFRYFLVPYICGKS---
32398951_Cryptosporidium_parvu     EVVPENPNYLK--ECGFDSCWIHSSYYDIISQFRNQK---
T_gondii_TgTwinscan4106            EVTPENPQICN--SAGFDSCWIHSAYYDAIKVTRGQD---
23123689_Nostoc_punctif            EDHSDWEPVTEVGGLGFDAAWYANFYHHLIGDARQGTEYA
23050680_Methanosarcina_barker     EQLQKPVEILNCTWQNRTLNTARGIYGNCEKLTELGSLFG
                                   *
```
